# Selenium-Substituted Monomethine Cyanine Dyes as Selective G-Quadruplex Spectroscopic Probes with Theranostic Potential

**DOI:** 10.3390/biom13010128

**Published:** 2023-01-07

**Authors:** Ivana Fabijanić, Atanas Kurutos, Ana Tomašić Paić, Vanja Tadić, Fadhil S. Kamounah, Lucija Horvat, Anamaria Brozovic, Ivo Crnolatac, Marijana Radić Stojković

**Affiliations:** 1 Division of Organic Chemistry and Biochemistry, Ruđer Bošković Institute, Bijenička Cesta 54, 10000 Zagreb, Croatia; 2 Institute of Organic Chemistry with Centre of Phytochemistry, Bulgarian Academy of Sciences, Acad. G. Bonchev Str., Bl. 9, 1113 Sofia, Bulgaria; 3 Division of Molecular Biology, Ruđer Bošković Institute, Bijenička Cesta 54, 10000 Zagreb, Croatia; 4 Department of Chemistry, University of Copenhagen, Universitetsparken 5, DK-2100 Copenhagen, Denmark

**Keywords:** selenium-substituted cyanine dyes, DNA/RNA interaction, G-quadruplex selective recognition, Induced Circular Dichroism, mitochondrial accumulation, cell viability inhibitory effect

## Abstract

The binding interactions of six ligands, neutral and monocationic asymmetric monomethine cyanine dyes comprising benzoselenazolyl moiety with duplex DNA and RNA and G-quadruplex structures were evaluated using fluorescence, UV/Vis (thermal melting) and circular dichroism (CD) spectroscopy. The main objective was to assess the impact of different substituents (methyl vs. sulfopropyl vs. thiopropyl/thioethyl) on the nitrogen atom of the benzothiazolyl chromophore on various nucleic acid structures. The monomethine cyanine dyes with methyl substituents showed a 100-fold selectivity for G-quadruplex versus duplex DNA. Study results indicate that cyanines bind with G-quadruplex via end π-π stacking interactions and possible additional interactions with nucleobases/phosphate backbone of grooves or loop bases. Cyanine with thioethyl substituent distinguishes duplex DNA and RNA and G-quadruplex structures by distinctly varying ICD signals. Furthermore, cell viability assay reveals the submicromolar activity of cyanines with methyl substituents against all tested human cancer cell lines. Confocal microscopy analysis shows preferential accumulation of cyanines with sulfopropyl and thioethyl substituents in mitochondria and indicates localization of cyanines with methyl in nucleus, particularly nucleolus. This confirms the potential of examined cyanines as theranostic agents, possessing both fluorescent properties and cell viability inhibitory effect.

## 1. Introduction

The design of small molecules that selectively bind to DNA and RNA (single-, double-stranded, triple-stranded or quadruplex nucleic acid structures) is an important area of current research due to the relevance of such recognition events in molecular biology and medicine [1,2].

Noncovalent recognition of DNA/RNA by small molecules includes intercalation (insertion between nucleobases), binding into the grooves, and electrostatic interactions of positively charged molecules with the phosphate backbone of DNA/RNA, or a combination of the binding modes [3].

Cyanine chromophores are often used as building blocks of fluorescent dyes due to their negligible fluorescence in free form and strong emission upon binding to DNA and RNA [4,5].

Detailed quantum mechanical and spectroscopic studies of the TO (Thiazole orange, cyanine dye) chromophore implied that small changes in the structure or environment of the chromophore during interaction with DNA can strongly affect the fluorescence response, the affinity, and the mode of binding [6,7]. Our recent work was focused on investigating the influence of steric and electronic properties of different substituents of cyanine dyes on DNA and RNA binding.

For example, cyanine dyes with phosphonium substituents showed unusual kinetic recognition between minor grooves of homo and alternating AT-DNA sequences while guanidiniocarbonyl-pyrrole and bromo TO derivative enabled selective recognition of double-stranded (ds-) RNA vs. ds-DNA [8,9,10]. Similar discrimination between ds-DNA and ds-RNA structures was shown by oxazole yellow homodimer (YOYO), whereas chlorinated TOTO (thiazole orange homodimer) demonstrated preferential binding to alternating DNA sequences compared to non-halogenated TOTO [11]. Furthermore, TO and TOTO with hydroxypropyl functionality could distinguish between GC- and AT-DNA by the binding affinity and the strong fluorescence signal [12]. Styryl dyes with *N*-Methylpiperazine and *N*-Phenylpiperazine functionalities demonstrated preferred binding to AT-DNA and G-quadruplex, respectively. Furthermore, the compounds exhibited submicromolar antiproliferative activity which in addition to strong fluorescence increase confirmed their potential as theranostic agents [13].

Recently, we reported on dicationic monomethine cyanine dyes (with halogen and halogen free) that induced the triplex formation of consecutive rA/dA-containing nucleic acid helices. Chlorination of cyanine dye resulted in selective stabilization of triplex helices vs. ds-nucleic acid structures and a strong tendency for H-aggregate formation in the presence of rA chain in single-stranded, double-stranded, or triple-stranded polynucleotides [14].

The introduction of the selenium into cyanine dyes resulted in fluorescent probes for detection of intracellular reactive oxygen and nitrogen species or development photosensitizers for NIR (Near-InfraRed) photodynamic therapy [15,16]. Indolium, quinolinium, pyridinium, benzothiazolium and benzoxazolium, which are positively charged nitrogen heterocycles, are typical building blocks for the development of cyanine platforms. Nonetheless, benzoselenazolium, the isosteric analog of benzoxazoles and benzothiazoles, has rarely been investigated. As sulfur homologues, selenium-containing compounds, especially benzoselenazoles, have recently found several uses in materials science, organic synthesis and medicinal chemistry [17,18,19,20,21]. The exchange of sulfur to a selenium atom could lead to the desired red shift in the absorption and emission spectra [22]. Even based on these limited reports, it is clear that benzoselenazolium-based cyanine dyes can identify nucleic acids in cells. In creating new fluorescent probes, benzoselenazolium-based dyes deserve additional modifications and extensive research.

Recently, a short study presented the synthesis of neutral and monocationic asymmetric monomethine cyanine dyes possessing a selenium heteroatom and their characterization in methanol [23]. In addition, their potential as fluorescent dyes for nucleic acid labeling are briefly demonstrated.

**Figure 1:**
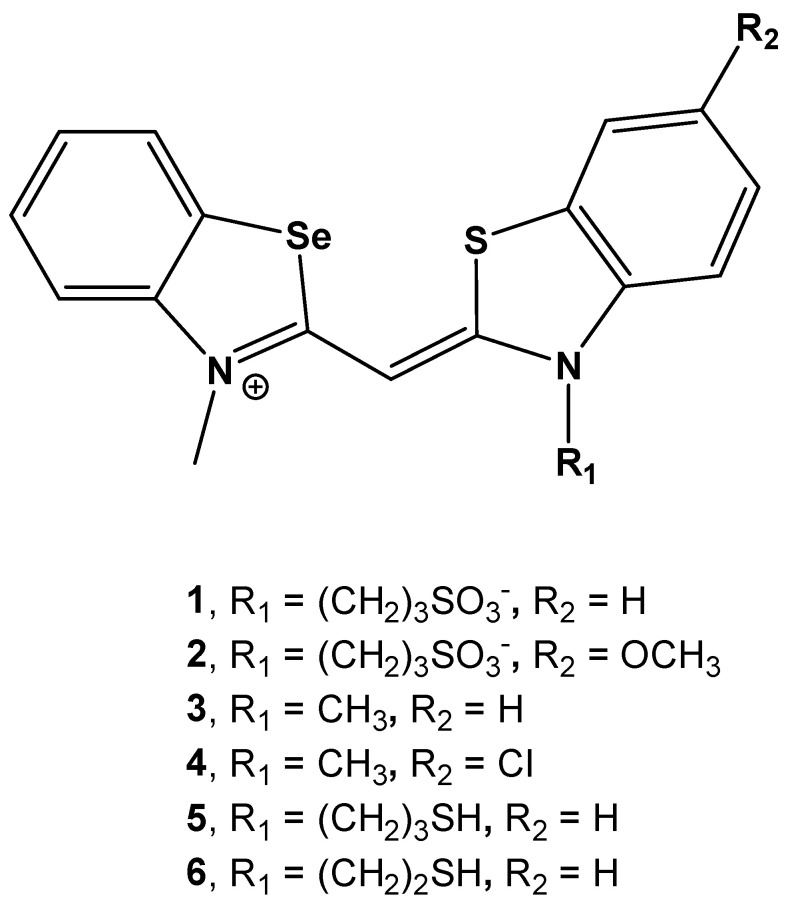
Structures of monomethine cyanine dyes **1–6** with benzoselenazolyl unit [23].

Here, we present a thorough characterization of the binding interaction between selenium-substituted cyanine dyes [23] with DNA and RNA (Figure 1). The objectives of this work included (1) studies of the impact of different substituents (methyl vs. sulfopropyl vs. thiopropyl/thioethyl) on the nitrogen atom of the benzothiazolyl chromophore on ds- and quadruplex nucleic acid structures (2) impact of compounds on cell viability and determination of their intracellular localization. We used UV/Vis and fluorescence spectroscopy, thermal melting experiments, and circular dichroism (CD) spectroscopy to determine the binding affinity, spectroscopic feedback and the binding mode. Further, MTT assay and confocal microscopy were utilized to explore the theranostic potential of here presented cyanine dyes.

## 2. Materials and Methods

### 2.1. Materials and Synthetic Methods

Using a combination of ^1^H-NMR, ^13^C-NMR, ^77^Se-NMR spectroscopy and high-resolution mass spectrometry (HRMS) in ESI mode, all dyes’ chemical identities were validated [23]. The ^1^H-NMR, ^13^C-NMR and ^77^Se-NMR spectra were acquired using 5 mm tubes on a Bruker Ultrashield Plus 500 spectrometer in DMSO-d_6_ at 25 °C. Their respective operating frequencies were 500.13 MHz and 125.77 MHz. Chemical shifts are given with an accuracy of 0.01 parts per million (ppm). The coupling constants (*J*) are determined to an accuracy of 0.1 Hz. The ^1^H-NMR spin multiplicity was described using the following acronyms: s = singlet, d = doublet, t = triplet, q = quartet, dd = doublet of doublets and m = multiplet. The HRMS spectra were acquired on a 40 °C maintained Dionex Acclaim RSLC 120C18 2.2 mm 120 2.1 50 mm column. The measurements were carried out with a Bruker MicrOTOF-QII system and an ESI source with a nebulizer under the following conditions: 1.2 bar, dry gas 8.0 L min^−1^, dry temperature 200 °C, capillary 4500 V and plate offset 500 V.


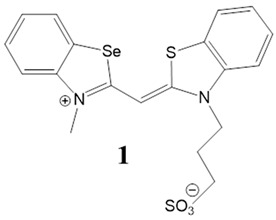


3-(2-((3-methylbenzo[d][1,3]selenazol-3-ium-2-yl)methylene)benzo[d]thiazol-3(2H)-yl)propane-1-sulfonate (compound **1**): Yield of crude product = 29%; m.p. > 300 °C; R_f_ = 0.51 (chloroform: methanol: acetic acid, 86:13:1); ^1^H NMR (500 MHz, DMSO-*d*_6_) δ 8.26 (dd, *J* = 8.0, 2.1 Hz, 1H), 8.22 (dd, *J* = 8.1, 2.0 Hz, 1H), 7.98 (d, *J* = 8.3 Hz, 1H), 7.86 (dd, *J* = 8.7, 2.6 Hz, 1H), 7.73–7.62 (m, 2H), 7.51 (td, *J* = 7.3, 6.4, 1.9 Hz, 1H), 7.43 (t, *J* = 7.7 Hz, 1H), 7.33 (s, 1H), 4.92–4.80 (m, 2H), 4.08 (s, 2H), 2.73–2.66 (m, 2H), 2.15 (d, *J* = 8.9 Hz, 2H); ^13^C NMR (126 MHz, DMSO-*d*_6_) δ 166.47, 162.65, 151.63, 142.35, 140.43, 128.65, 128.28, 126.31, 126.22, 124.93, 124.71, 124.58, 123.66, 115.36, 113.74, 87.30, 47.18, 45.33, 35.64, 23.09; ^77^Se NMR (95 MHz, DMSO-*d*_6_) δ 462.62. HRMS: m/z: Found 466.99977 [M] C_19_H_18_N_2_O_3_S_2_Se; Requires [M] 467.00002.


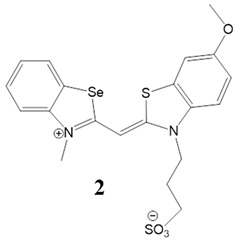


3-(6-methoxy-2-((3-methylbenzo[d][1,3]selenazol-3-ium-2-yl)methylene)benzo[d]thiazol-3(2H)-yl)propane-1-sulfonate (compound **2**): Yield of crude product = 21%; m.p. > 300 °C; R_f_ = 0.54(chloroform: methanol: acetic acid, 86:13:1); ^1^H NMR (400 MHz, DMSO-*d*_6_) δ 8.26 (dd, *J* = 8.0, 1.3 Hz, 1H), 8.10 (d, *J* = 8.9 Hz, 1H), 7.87 (d, *J* = 8.1 Hz, 1H), 7.69–7.60 (m, 2H), 7.46–7.38 (m, 1H), 7.27 (s, 1H), 7.12 (dd, *J* = 8.9, 2.3 Hz, 1H), 4.85 (t, *J* = 8.2 Hz, 2H), 4.07 (s, 3H), 3.93 (s, 3H), 2.67 (td, *J* = 4.3, 2.5 Hz, 2H), 2.13 (d, *J* = 8.6 Hz, 2H); ^77^Se NMR (95 MHz, DMSO-*d*_6_) δ 462.67. HRMS: m/z: Found 497.00998 [M] C_20_H_20_N_2_O_4_S_2_Se; Requires [M] 497.0108.


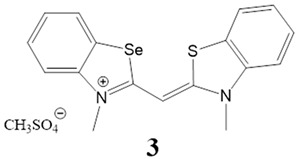


3-methyl-2-((3-methylbenzo[d]thiazol-2(3H)-ylidene)methyl)benzo[d][1,3]selenazol-3-ium methosulphate (compound **3**): Yield of crude product = 47%; m.p. > 300 °C; R_f_ = 0.16 (chloroform: methanol: acetic acid, 86:13:1); ^1^H NMR (500 MHz, DMSO-*d*_6_) δ 8.14 (dd, *J* = 8.1, 1.2 Hz, 1H), 8.11 (dd, *J* = 8.0, 1.1 Hz, 1H), 7.80 (d, *J* = 8.4 Hz, 1H), 7.75 (d, *J* = 8.4 Hz, 1H), 7.64 (ddd, *J* = 8.4, 7.3, 1.2 Hz, 1H), 7.60 (ddd, *J* = 8.4, 7.3, 1.2 Hz, 1H), 7.51–7.44 (m, 1H), 7.42–7.35 (m, 1H), 6.76 (s, 1H), 3.94 (s, 3H), 3.93 (s, 3H), 3.84 (s, 3H); ^13^C NMR (126 MHz, DMSO-*d*_6_) δ 166.43, 163.62, 142.64, 141.42, 129.36, 129.08, 126.70, 126.41, 125.77, 125.43, 124.83, 123.97, 115.74, 114.36, 87.43, 35.54, 34.61; ^77^Se NMR (95 MHz, DMSO-*d*_6_) δ 462.67. HRMS: m/z: Found 359.01149 [M+] C_17_H_15_N_2_SSe; Requires [M+] 359.0116.


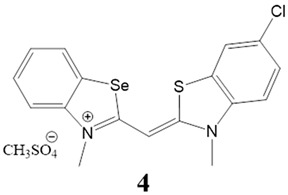


2-((6-chloro-3-methylbenzo[d]thiazol-2(3H)-ylidene)methyl)-3-methylbenzo[d][1,3]selenazol-3-ium methosulphate (compound **4**): Yield of crude product = 52%; m.p. > 300 °C; R_f_ = 0.14 (chloroform: methanol: acetic acid, 86:13:1); ^1^H NMR (500 MHz, DMSO-*d*_6_) δ 8.37 (d, *J* = 1.8 Hz, 1H), 8.27 (d, *J* = 7.9 Hz, 1H), 7.87 (dd, *J* = 12.5, 8.6 Hz, 2H), 7.76–7.70 (m, 1H), 7.66 (t, *J* = 7.8 Hz, 1H), 7.44 (t, *J* = 7.6 Hz, 1H), 6.85 (s, 1H), 4.01 (s, 3H), 3.99 (s, 3H); ^13^C NMR (126 MHz, DMSO-*d*_6_) δ 166.60, 163.17, 142.21, 140.20, 128.89, 128.73, 128.47, 126.49, 126.23, 126.02, 124.94, 123.22, 115.54, 115.18, 87.30, 35.31, 34.46; ^77^Se NMR (95 MHz, DMSO-*d*_6_) δ 462.60. HRMS: m/z: Found 392.97251 [M+] C_17_H_14_ClN_2_SSe; Requires [M+] 392.9726.


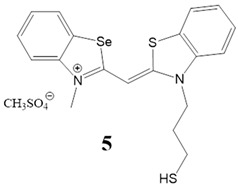


2-((3-(3-mercaptopropyl)benzo[d]thiazol-2(3H)-ylidene)methyl)-3-methylbenzo[d][1,3]selenazol-3-ium methosulphate (compound **5**): Yield of crude product = 63%; m.p. = 233–236 °C; R_f_ = 0.17 (chloroform: methanol: acetic acid, 86:13:1); ^1^H NMR (400 MHz, DMSO-*d*_6_) δ 8.26 (dd, *J* = 8.0, 1.3 Hz, 1H), 8.20 (dd, *J* = 8.0, 1.2 Hz, 1H), 7.87 (d, *J* = 8.3 Hz, 1H), 7.81 (dd, *J* = 8.5, 1.1 Hz, 1H), 7.65 (dddd, *J* = 8.5, 7.3, 4.9, 1.3 Hz, 2H), 7.47 (ddd, *J* = 8.2, 7.3, 0.9 Hz, 1H), 7.43 (ddd, *J* = 8.1, 7.2, 1.0 Hz, 1H), 6.84 (s, 1H), 4.66 (t, *J* = 7.4 Hz, 2H), 3.96 (s, 3H), 3.37 (s, 3H), 2.91 (t, *J* = 6.9 Hz, 2H), 2.16 (p, *J* = 7.2 Hz, 2H); ^13^C NMR (126 MHz, DMSO-*d*_6_) δ 166.67, 163.18, 142.41, 140.76, 129.52, 129.25, 126.73, 126.57, 125.95, 125.66, 124.91, 124.05, 115.78, 114.18, 86.92, 54.26, 45.64, 35.64, 35.32, 26.39; ^77^Se NMR (95 MHz, DMSO-*d*_6_) δ 462.68. HRMS: m/z: Found 418.00805 [M+] C_19_H_19_N_2_S_2_Se; Requires [M+] 418.0071.


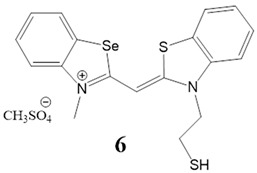


2-((3-(2-mercaptoethyl)benzo[d]thiazol-2(3H)-ylidene)methyl)-3-methylbenzo[d][1,3]selenazol-3-ium methosulphate (compound **6**): Yield of crude product = 51%; m.p. =245–247 °C; R_f_ = 0.11 (chloroform: methanol: acetic acid, 86:13:1); ^1^H NMR (500 MHz, DMSO-*d*_6_) δ 8.28 (dd, *J* = 8.0, 1.3 Hz, 1H), 8.20 (dd, *J* = 8.0, 1.2 Hz, 1H), 7.90 (d, *J* = 8.4 Hz, 1H), 7.83 (d, *J* = 8.4 Hz, 1H), 7.66 (tt, *J* = 8.4, 1.8 Hz, 2H), 7.49 (t, *J* = 7.7 Hz, 1H), 7.48–7.41 (m, 1H), 6.93 (s, 1H), 4.90 (t, *J* = 7.2 Hz, 2H), 3.99 (s, 3H); ^13^C NMR (126 MHz, DMSO-*d*_6_) δ 205.36, 201.54, 180.95, 179.19, 167.49, 167.28, 165.36, 165.12, 163.99, 163.71, 163.18, 162.60, 154.22, 152.67, 126.05, 91.64, 84.46, 74.14, 72.93; ^77^Se NMR (95 MHz, DMSO-*d*_6_) δ 462.56. HRMS: m/z: Found 403.99157 [M+] C_18_H_17_N_2_S_2_Se; Requires [M+] 403.9915.

### 2.2. Materials and Spectrophotometric Methods

The UV/vis spectra were recorded on a Varian Cary 100 Bio spectrophotometer (Agilent, Santa Clara, CA, USA), CD spectra on JASCO J815 spectrophotometer (ABL&E Handels GmbH, Wien, Austria) and fluorescence spectra on a Varian Cary Eclipse spectrophotometer (Agilent, Santa Clara, CA, USA) at 25 °C using appropriate 1 cm path quartz cuvettes.

Materials. Polynucleotides were purchased as noted: poly A–poly U, calf thymus ctDNA, poly(dAdT)_2_ and poly(dGdC)_2_ (Sigma-Aldrich, St. Louis, MI, USA). Polynucleotides were dissolved in Na-cacodylate buffer, *I* = 0.05 mol dm^−3^, pH = 7.0. The calf thymus ctDNA was additionally sonicated and filtered through a 0.45 mm filter [24]. Polynucleotide concentration was determined spectroscopically as the concentration of phosphates [25,26]. Spectrophotometric titrations were performed at pH = 7.0 (*I* = 0.05 mol dm^−3^, sodium cacodylate buffer) by adding aliquots of polynucleotide solution into the solution of the studied compound for fluorimetric experiments and CD experiments were done by adding aliquots of the compound stock solution into the solution of a polynucleotide. In fluorimetric experiments, an excitation wavelength of λ_exc_ = 425, 431, 423, 427, 406 and 407 nm, respectively, was used to avoid the inner filter effect caused due to increasing absorbance of the polynucleotide. Emission was collected in the range λ_em_ = 400–700 nm. Values for *K*_a_ obtained by processing titration data using the Scatchard equation [27], all have satisfactory correlation coefficients (≥0.98).

Thermal melting curves for DNA, RNA and their complexes with studied compounds were determined as previously described by following the absorption change at 260 nm as a function of temperature. The absorbance of the ligands was subtracted from every curve and the absorbance scale was normalized. *T*_m_ values are the midpoints of the transition curves determined from the maximum of the first derivative and checked graphically by the tangent method. The ∆*T*_m_ values were calculated by subtracting *T*_m_ of the free nucleic acid from *T*_m_ of the complex. Every ∆*T*_m_ value here reported was the average of two measurements. The error in ∆*T*_m_ is ±0.5 °C. 5′-AGGG(TTAGGG)_3_–3′(Tel22) was obtained from IDT (Integrated DNA Technologies, Coralville, IA, USA). Tel22 was dissolved in 0.1 M either in sodium cacodylate or potassium phosphate buffer. The starting Tel22 oligonucleotide solution was first heated up to 95 °C for 10 min, then slowly cooled to 10 °C at the cooling rate of 1 °C/min to allow DNA oligonucleotide to adopt a G-quadruplex structure. The G-quadruplex structure was confirmed by thermal melting and CD spectra [28]. The concentration of G quadruplex was expressed in terms of oligonucleotide structure. In fluorimetric titrations, aliquots of Tel 22 solution were added to the solution of ligands (c ~ 2 × 10^−7^ M, in SI).

### 2.3. Effect of Compounds on Cell Viability

#### 2.3.1. Cell Lines

Human cervical carcinoma HeLa (GIBCO BRL, Invitrogen, Waltham, MA, USA), human melanoma MDA-MB-435S and human ovarian adenocarcinoma SK-OV-3 (ATCC-LGC Standards GmbH, Wesel, Germany) cell lines were purchased from indicated cell culture banks. Normal human skin fibroblasts were isolated from the upper arm of a 7-year-old female donor at the Neurochemical Laboratory, Department of Chemistry and Biochemistry, School of Medicine, University of Zagreb. HeLa, MDA-MB-435S and fibroblasts were grown in Dulbecco’s Modified Eagle’s Medium (DMEM; Sigma-Aldrich, St. Louis, MI, USA) while the SK-OV-3 cell line was grown in McCoy’s 5A medium (Capricorn Scientific, Ebsdorfergrund, Germany). All media were supplemented with 10% fetal bovine serum (FBS; Sigma-Aldrich) and 50 I.U./mL penicillin and 50 μg/mL streptomycin. Cells were cultured in a humidified atmosphere of 5% CO_2_ at 37 °C and were sub-cultured every 3–4 days.

#### 2.3.2. Cytotoxicity Assay

HeLa, MDA-MB-435S, SK-OV-3 cells and fibroblasts were seeded in 96 well plates. Twenty-four hours later the cells were treated, in quadruplicate, with different concentrations of compounds (dissolved in DMSO, kept at 4 °C and protected from light). Following 72 h of incubation at 37 °C, the medium was aspired and modified 3-(4,5-dimethylthiazol-2-yl)-2,5-diphenyltetrazolium bromide (MTT) [29] assay was used to determine the cytotoxic effect of the tested compounds. Three hours later, formazan crystals were dissolved in DMSO (0.17 mL/well), the plates were mechanically agitated for 20 min and the optical density at 600 nm was determined on a microtiter plate reader (Awareness Technology Inc., Palm City, FL, USA). The experiments were repeated three times.

### 2.4. Co-Localization Assay

Live imaging of the cells treated with compounds was performed on the HeLa cell line. Cells were seeded in Ibidi imaging cell chambers (Ibidi^®^, Gräfelfing, Germany) in 500 μL of a medium, with the concentration of 7.5 × 10^4^ cells/well and left in the cell incubator for 48 h (37 °C, 5% CO_2_). After two days, the cells were treated with a 1 μM solution of each compound and left in the cell incubator for 60 min to allow the compound to enter the cells. After the incubation, the medium was changed, and 500 μL of 100 nM MitoTracker Deep Red solution (Invitrogen, Molecular Probes) was added to the chambers. Cells were incubated for 20 min (37 °C, 5% CO_2_), allowing MitoTracker to enter the cells. After incubation, the medium was replaced with 500 μL of fresh medium. Co-localization of compounds (λ_exc_ = 405 nm, λ_em_ = 470–670 nm) and mitochondria (MitoTracker λ_exc_ = 644 nm, λ_em_ = 665 nm) was then visualized and confirmed using Leica SP8 X confocal microscope (Leica Microsystems, Wetzlar, Germany). Control and untreated cells excluded the presence of foreign fluorescence.

## 3. Results and Discussion of Spectrophotometric Study

### 3.1. Characterization of Compounds in Aqueous Medium

Studied compounds, **1–6** were soluble (*c* = 8 × 10^−4^ mol dm^−3^) in DMSO. However, cyanine **2** was prone to aggregation after several weeks even in DMSO. Therefore, the stock solution of **2** was prepared fresh before use. The experiments were done in aqueous buffer (sodium cacodylate buffer, *I* = 0.05 mol dm^−3^; pH = 7.0). 

Absorption maxima and the corresponding molar extinction coefficients (ε) of investigated monomethine cyanine dyes are summarized in Table 1. 

Linear changes in the UV/Vis spectra of monomethine cyanines **1**, **3** and **4** in the range of concentrations (8 × 10^−7^–4 × 10^−6^ mol dm^−3^) imply that investigated compounds do not form aggregates by intermolecular stacking (Figures S1–S7 in Supporting Information (SI)). However, the shape of UV/Vis spectra of cyanine **2** changed with increasing concentration (calibration experiment in Figure S2 in SI). In addition to the dominant maximum at 432 nm (which most likely corresponds to the monomer form of the dye), the absorption band at 413 nm becomes more intense (Figure S2 in SI). Presumably, it corresponds to the aggregated form (H-aggregate) [4,11,30]. Based on the existence of an absorption band at 432 nm, only the monomer dye form is visible at high temperature (95 °C) (Figure S8b in SI). However, after cooling the solution to 25 °C, an additional band appears at 413 nm, which corresponds to the aggregated dye form. This suggests that dye **2** is present in the solution as a mixture of monomer and aggregated form at 25 °C. 

**Figure 2:**
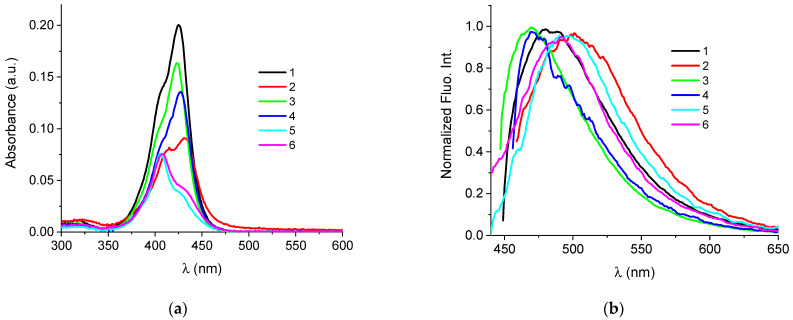
(**a**) UV Vis spectra of **1** to **6** at *c* = 2.39 × 10^−7^ mol dm^−3^, at pH = 7, sodium cacodylate buffer, *I* = 0.05 mol dm^−3^; (**b**) Normalized spectra of **1** (λ_exc_ = 425 nm), **2** (λ_exc_ = 431 nm), **3** (λ_exc_ = 423 nm), **4** (λ_exc_ = 427 nm), **5** (λ_exc_ = 406 nm) and **6** (λ_exc_ = 407 nm), at pH = 7.0, sodium cacodylate buffer, *I* = 0.05 mol dm^−3^.

In the absorption spectra of cyanines **5** and **6**, the dominant maximum was at 407 nm, while the shoulder at 426 nm had much lower intensity (Figure 2, Figures S5 and S6 in SI). Based on this, it can be assumed that cyanines **5** and **6** significantly aggregate in aqueous solutions. This is supported by the lowest molar extinction coefficients and the fact that even at higher temperatures, the aggregated form remains dominant (Figure 2, Table 1 and Figure S8 in SI). 

Buffered solutions of the cyanine dyes at *c* = 5 × 10^−7^ mol dm^−3^ possess minor intrinsic fluorescence when excited at their corresponding longest-wavelength absorbance maxima (Figure 2, Table 1). 

Therefore, the aggregation potential of **1–6** dyes depended on the nature of the substituent on the nitrogen atom of the thiazole moiety. Compounds with sulfopropyl and methyl groups (**1**, **3** and **4**) were present in aqueous solutions mainly in the form of monomers. Additional methoxy substituent on the N atom of the thiazole unit induced the aggregation of compound **2**. Furthermore, the presence of thiopropyl/thioethyl groups promoted the aggregation of dyes **5** and **6** in aqueous environments.

### 3.2. Study of Interactions of 1–6 with Nucleic Acids in Aqueous Medium

#### 3.2.1. Interactions with ds-DNA and ds-RNA

First, we investigated the binding of **1–6** cyanines with synthetic double-stranded polynucleotides, differing in base composition, grooves size and conformation. Alternating AT and GC polynucleotides were chosen as models for B-helix DNA and AU homopolynucleotide as a model for A-helical structure (RNA) [3,31,32]. As noted for most cyanine dyes [4,12,14], the addition of ds-DNA (AT-DNA, GC-DNA) or ds-RNA (AU-RNA) to solutions of compounds **1–6** led to an increase in their emission (see Figures S9–S26 in SI). Similarly, to recently reported styryl cyanine dyes [13], hypsochromic shifts (6–20 nm) were noticed in all titrations of **1–6** after adding aliquots of ds-DNA and ds-RNA.

Furthermore, linear changes were evident with increasing DNA or RNA concentration in most titrations. The exceptions to this were titrations of cyanine **3** with GC-DNA and titrations of dye **5** with ds-DNA and ds-RNA where sufficient data points in the non-linear part of the curve allowed estimation of binding affinity (*K*_a_ values were of the order 10^5^ mol^−1^ dm^−3^; Figures S16, S22 and S23 in SI). Titration data were collected at a high excess of polynucleotide binding sites (r[1–6]/[nucleotide phosphate] < 0.1; see Figures S9–S26 in SI) where each molecule can bind to a separate binding site. Binding affinities of dye-DNA/RNA complexes were calculated by non-linear fitting using the Scatchard equation [27,33]. The fluorescence changes of dye **5** in the titration with AT-DNA were too small to obtain an accurate binding constant (Figure S21 in SI).

Binding abilities of **1–6** compounds were further explored by thermal melting experiments [34]. The difference between the *T*_m_ value of free nucleic acid structure and the complex with a small molecule (∆*T*_m_ value) can indicate the mode of their interaction. For example, positive ∆*T*_m_ values (>4–5 °C) is characteristic for intercalators and classical minor groove binders [35]. On the other hand, lower ∆*T*_m_ values or lack of stabilization suggest either a partial intercalation or binding of aggregated molecules inside DNA/RNA grooves and/or along the polynucleotide backbone.

Mostly, the compounds **1–6** did not show a stabilizing effect on ds-DNA and ds-RNA at ratio, **r** [compound]/[nucleotide phosphate] = 0.1 (Figures S27–S29 in SI). Only compound **5** with thiopropyl substituent on the N atom of the benzothiazolyl chromophore exhibited the stabilization effect on AT-DNA (∆*T*_m_ value = 4 °C) [10,35,36]. Close derivative **6** with thioethyl substituent did not show any impact on DNA/RNA thermal stability.

Circular dichroism spectroscopy can provide valuable information on the interaction of small molecules with DNA or RNA. Even achiral molecules, such as dyes **1–6**, can induce CD signals upon binding to DNA and RNA. The most informative area in CD spectrum is the wavelength range higher than 300 nm where compounds absorb, and DNA/RNA do not. Different binding modes (intercalation, groove binding and binding of aggregated molecules to the polynucleotide backbone) cause the formation of induced CD signals (ICD) of varying intensity and sign [37]. For instance, classical minor groove binders induce strong positive ICD signals, whereas intercalation can either give a weak negative or weak positive ICD band depending on the arrangement of the small molecule with the base pairs [38,39].

**Figure 3:**
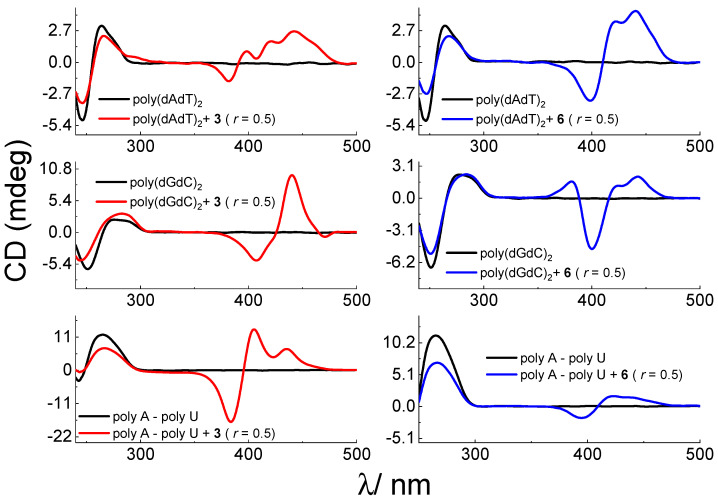
CD spectra of AT- and GC-DNA and AU-RNA (*c* = 3.0 × 10^−5^ mol dm^−3^) with dye **3** and **6** at molar ratios **r** = [compound]/[nucleotide phosphate] = 0.5 (pH = 7.0, buffer sodium cacodylate, *I* = 0.05 mol dm^−3^).

The addition of compounds **1–6** mostly caused a bigger (**4–6**) or smaller decrease (**1–2**) in CD band intensity of ds-DNA and ds-RNA (260–275 nm), suggesting a significant impact on polynucleotide secondary structure (Figure 3, Figure S30 in SI).

The addition of neutral dyes **1** and **2** with sulfopropyl substituents to DNA and RNA solutions resulted in negligible ICD bands which imply that cyanine units were not uniformly oriented in respect to the polynucleotide chiral axis. In contrast, strong ICD bands appeared in the absorption region between 300–480 nm in the case of **3–6** (Figure 3, Figure S30 in SI). ICD signals were of different intensity, shape and sign for DNA (B-type helix) and RNA (A-type helix). Similar differentiation of B- and A-helical structures were recently reported with cyanine monomethine dyes and Cl-TOTO/YOYO [11,14]. Moreover, cyanines **3–6** induced different ICD profiles with AT- and GC-DNA. Such changes arise from sensing diverse secondary structures with different base pair compositions, different dimensions, sterical hindrances and electrostatic potential of their grooves [3,31].

In the titration of AT-DNA with compounds **3** and **4**, the most intense was a positive ICD signal at 440 nm, which is most likely the result of monomer binding in the minor groove [39].

Even at the lowest ratio, **r** (**r** = 0.1) with GC-DNA, compounds **3** and **4** induced bisignate positive/negative ICD signals (+440/−410 nm), indicative of cyanine dimerization/aggregation in the polynucleotide grooves (Figure 3, Figure S30 in SI). The zero-crossing point of bisignate bands was around 430 nm that agrees with the dominant absorption maximum of examined compounds [38].

Similar bisignate ICD profiles were visible in titrations of **3** and **4** with RNA (rArU). Nevertheless, those signals were hypsochromically shifted (+405/−385 nm) compared to those observed with GC-DNA (B-helix), with zero-crossing point around 400 nm correlating with the absorption aggregate maximum (Figure 3, Figure S30 in SI).

Interestingly, ICD profiles of cyanine **5** (with thiopropyl substituent) were similar to those of **3** and **4** with AT- and GC-DNA (Figure 3, Figure S30 in SI). However, different changes were noticed in CD profile of RNA, which was only a weak negative signal at 415 nm, which could be indicative of partial intercalation or a groove binding [5,39]. This is additionally supported by the lack of thermal stabilization of ds-RNA, which is not characteristic for the classical intercalation [36,38].

In contrast to **5**, a close derivative with thioethyl substituent (cyanine **6**) caused dominant bisignate ICD (+440/−400 nm) profiles with AT- and GC-DNA, even with RNA and zero-crossing point in the range 408–416 nm, agreeing with the absorption aggregate maximum.

#### 3.2.2. Interactions with G-Quadruplex

In addition to ds-DNA/RNA, we have explored the binding of cyanines with DNA G-quadruplex, a target thought to be associated with essential biological processes, such as DNA replication, expression of some oncogenes, maintenance of telomeres stability and telomerase inhibition in cancer cells [40,41,42].

Structural characteristics of monomethine cyanines **1–6** such as large aromatic surfaces and positive charges could enable favorable interactions with G-quadruplex.

The interactions with G-quadruplex are investigated using oligonucleotide Tel22 (5′-AGGG(TTAGGG)3–3′). This basket-type, intramolecularly folded oligonucleotide Tel22 consists of three G-tetrads connected with one diagonal and two lateral (TTA) loops in Na^+^ solution [43]. There are several reported G-quadruplex structures in K^+^ solution depending on the variants of the human telomere sequence [44,45]. Circular dichroism spectroscopy revealed that in K^+^ solution Tel22 exists as a mixture of mixed-parallel/antiparallel and chair-type G-quadruplex [28].

**Figure 4:**
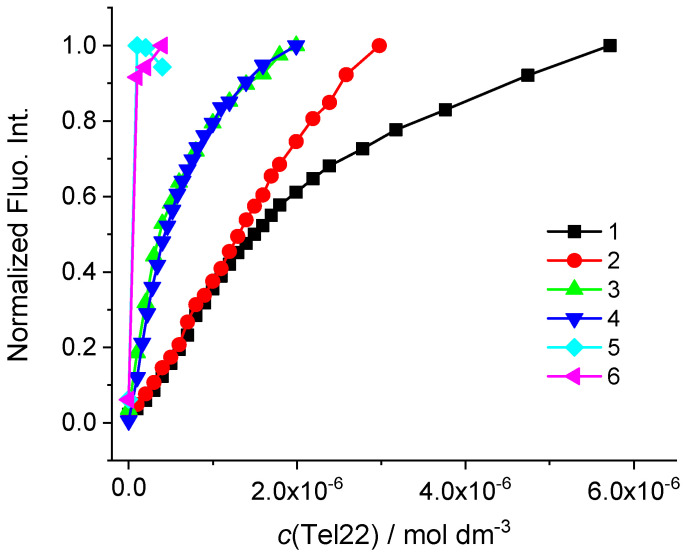
Changes of fluorescence emission of **1–6** (*c* = 2 × 10^−7^ mol dm^−3^) normalized to the last titration point (the highest emission intensity) upon the addition of Tel22 (sodium cacodylate buffer, *I* = 0.1 mol dm^−3^, pH = 7.0).

Initially, we did experiments with Tel22 in a sodium cacodylate buffer, *I* = 0.1 M, pH = 7.0. Monomethine cyanines **4–6** showed moderate stabilization effect of Tel22 structure (Table 2, Figure S37 in SI). Titration with Tel22 yielded significant fluorescence increase in all compounds (Figure 4).

Nevertheless, only cyanines **1**, **3** and **4** provoked such fluorimetric response that resulted in nonlinear curves, allowing the binding affinity calculation (Figure 4, Figures S31–S36 in SI).

Binding constants (log *K*_a_) and stoichiometry of complexes were calculated from the fluorimetric data using non-linear least-square program SPECFIT [46], in the concentration range that corresponded to 20–80% complex formed.

The experimental data of **1** and **3** was best fitted to a binding stoichiometry of ligand-Tel22 of 1:1 and 1:2. On the other hand, 1:1 stoichiometry was found for complex of Tel22 and **4** (Table 2).

**Figure 5:**
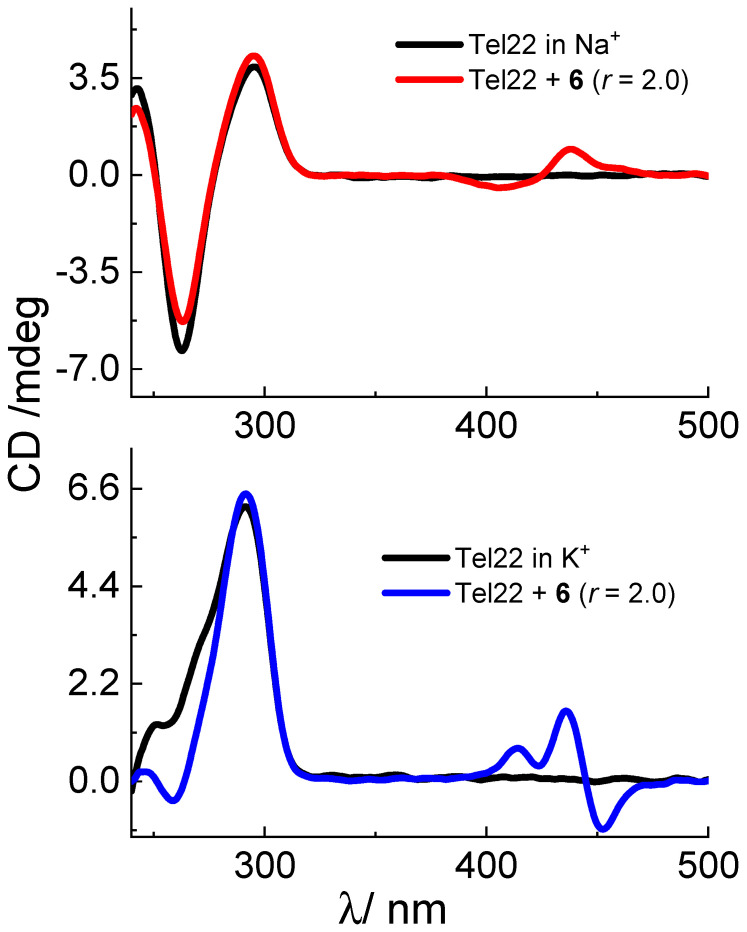
CD spectra of Tel22 (*c* = 1.5 × 10^−6^ mol dm^−3^) with dye **6** at molar ratios **r** = [compound]/[nucleotide phosphate] = 2.0 (pH = 7.0, *I* = 0.1 mol dm^−3^, buffer sodium cacodylate or potassium phosphate.

Compounds **1–4** displayed negligible changes on the characteristic CD peaks of Tel22 which suggest a slight perturbation of the DNA quadruplex secondary structure (Figure S38 in SI) [43]. Interestingly, compounds **5** and **6** displayed negative ICD signals at 413 and 440 nm and bisignate ICD band (+438/−408 nm; zero-crossing point around 425 nm), respectively (Figure 5, Figure S38 in SI). Such changes could imply binding of **5** and **6** in aggregated form with nucleobases/phosphate backbone of grooves or loops [47,48,49].

Considering the results, such as the binding affinities, 1:1 binding stoichiometry, stabilization effects of G-quadruplex and negligible changes of CD spectra of Tel22, obtained for **4**, we can assume that this compound most likely bind to the top or bottom quartets of Tel22 via end-stacking π-π interactions [49]. Unlike **4**, in the 1:2 established stoichometry with Tel22, compounds **1** and **3** most likely formed a sandwich-type complex, with the dye positioned between two quadruplex units (Table 2) [50].

Due to excellent binding to Tel22 in Na^+^ solution, we decided to examine the binding of compounds in K^+^ solution. G-quadruplex in K^+^ solution is considered to be biologically more relevant since the intracellular K^+^ concentration is greater than that of Na^+^ [28].

**Figure 6:**
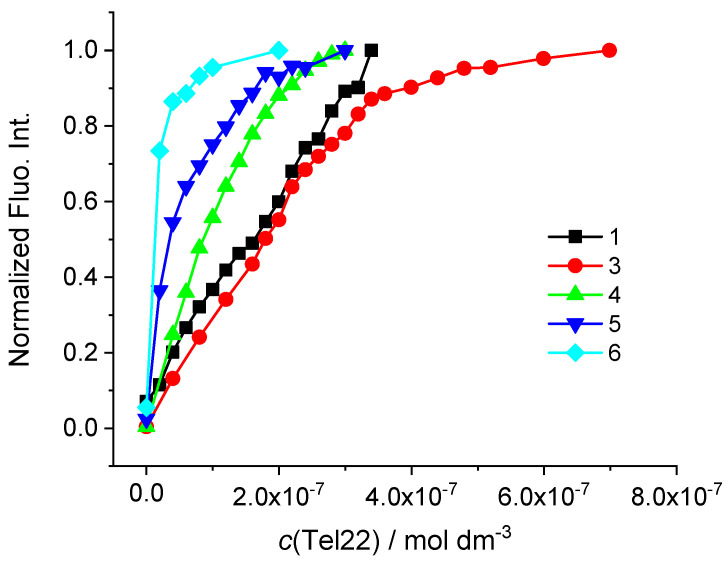
Changes of fluorescence emission of **1–6** (*c* = 2 × 10^−7^ mol dm^−3^) normalized to the last titration point (the highest emission intensity) upon addition of Tel22 (potassium phosphate buffer, *I* = 0.1 mol dm^−3^, pH = 7.0).

Similar to the Na^+^ solution, fluorescence of cyanines **1** and **3–6** in K^+^ solution increased after the addition of Tel22 (Figure 6, Figures S39–S43 in SI). In contrast to titrations in Na^+^ buffer, compounds **5** and **6** exhibited bigger emission increase with Tel22 in K^+^ solution. However, there were not enough data points in non-linear part of curves which enabled the calculation of binding affinities (Figure 6, Table 3, Figures S39–S43 in SI).

Nevertheless, the analysis of the fluorimetric data of **5** and **6** revealed a steeper slope of the titration curves indicating large stability constants (log *K*_a_ ≥ 7) comparable to those of compounds **3** and **4** (Figure 6). The best fit for titration data of **3** and **4** with Tel22 was obtained for 1:1 stochiometry. Compounds **1** and **3** exerted the strongest binding to Tel22 in the Na^+^ solution while the highest binding constant in the K^+^ solution was calculated for **4**-Tel22 complex. As in the Na^+^ solution, monomethine cyanines **4** and **5** showed moderate stabilization effect of Tel22 structure (Table 3, Figure S45 in SI). However, cyanine **6** with thioethyl substituent stabilized Tel22 structure significantly (Table 3).

Comparison of binding constants of compounds **3** and **4** with G-quadruplex (in the Na^+^ and K^+^ solution) and GC-DNA point to selective binding towards G-quadruplex with respect to duplex DNA (*K*_a_ values for Tel22 were 2 orders of magnitude higher than those estimated for duplex DNA, Table 2 and Table 3 and Figures S16, S22 and S23 in SI). Obtained binding affinities toward G-quadruplex is comparable or higher to those reported in the literature [13,47,48,50].

CD spectroscopy analysis with Tel22 in the K^+^ solution revealed small changes of intensities of a positive band at 290 nm and a shoulder at 255 nm upon the addition of studied compounds (Figure 5, Figure S44 in SI). However, significant induced CD signal bands appeared in the wavelength longer than 350 nm, in the compounds’ absorption area. Cyanines **3** and **4** induced positive ICD bands positioned at 410 and 435 nm (Figure 5, Figure S44 in SI). Furthermore, cyanines **5** and **6** caused bisignate ICD bands (−435/+413 nm for **5** and −450/+435 nm for **6**; zero-crossing points at 430 nm and 445 nm, respectively). Due to extended aromatic surfaces of examined cyanines, possible binding mode is π-π stacking with top or bottom G-tetrad. According to the ICD changes, additional binding interaction of **3–6** could be accomplished with nucleobases/phosphate backbone of grooves or loops in compound dominant monomer/aggregated form [47,48,49]. The preferred topology and interactions via groove/loop for G-quadruplex-ligand complex can be accurately determined with other methods (NMR, x-ray diffraction, molecular modelling) [51], however that goes beyond the scope of present work.

Intriguingly, compound **6** with Tel22 displayed bisignate ICD signals of opposite signs (negative/positive) compared to bisignate signals observed with duplex DNA and RNA (positive/negative). In particular, compound **6** could distinguish between Tel22 structures in the K^+^ and Na^+^ solutions by different signs of ICD bisignate bands, which were not previously reported.

## 4. Biological Activity

### 4.1. MTT Assay

**Figure 7:**
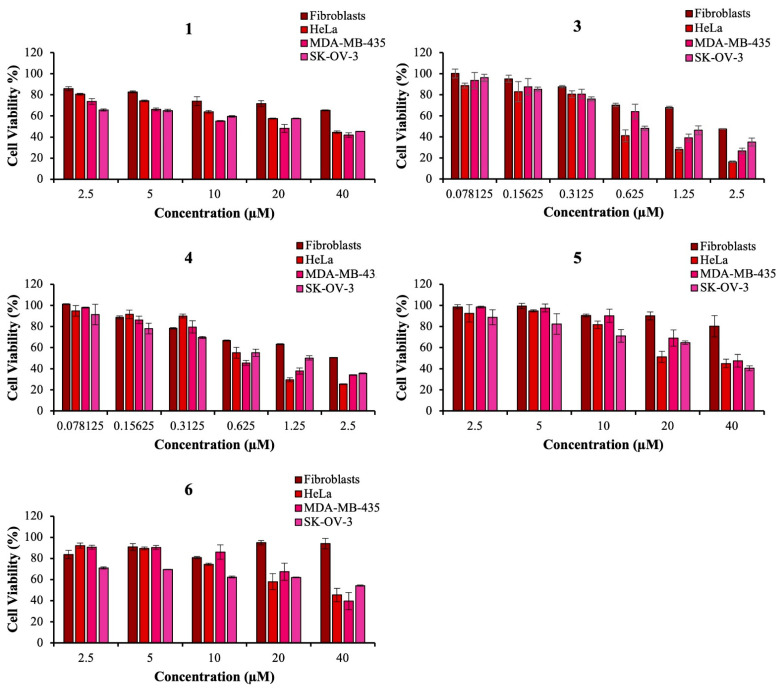
The effect of compounds **1**, **3**, **4**, **5** and **6** on different types of cancer cell lines and fibroblasts viability. HeLa, MDA-MB-435S and SK-OV-3 cell lines and fibroblasts were treated with indicated concentrations of analyzed compounds. The cell survival was measured 72 h after the treatment by MTT assay. The experiments were done in quadruplicate and repeated at least three times.

The effect of the compounds **1–6** on cell viability was analyzed by using different tumor cell lines (human cervical carcinoma HeLa, human melanoma MDA-MB-435S and human ovarian adenocarcinoma SK-OV-3) and 3-(4,5-dimethylthiazol-2-yl)-2,5-diphenyltetrazolium bromide (MTT) assay. Since one of the criteria for newly synthesized compounds to be considered as possible antitumor compounds is a low effect on normal cell viability and a non-selective effect on different tumor cell viability, we used the normal human fibroblasts to test the selectivity of the compounds toward tumor cell lines. The IC_50_ values obtained are indicated in Table 4. The data showed that the strongest effect on the viability of tested cell lines had compounds **3** and **4** (Figure 7). These compounds could be interesting candidates for further investigation. Compounds **5** and **6** showed moderate effect on the viability of all analyzed cell lines in a relevant concentration range. The IC_50_ values of all analyzed compounds for the normal human fibroblasts were above the IC_50_ values measured for tested tumor cell lines.

### 4.2. Confocal Microscopy

**Figure 8:**
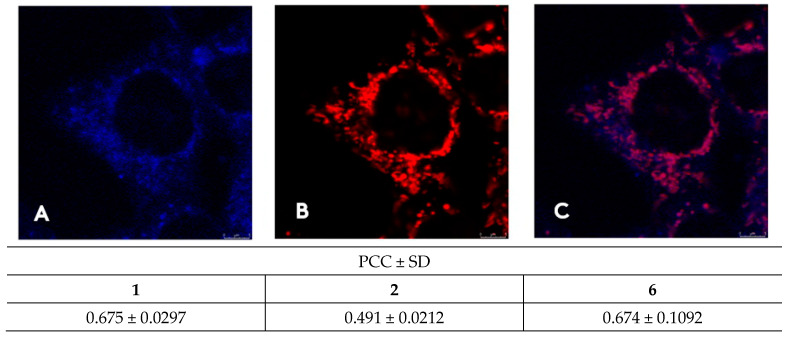
Intracellular distribution of dye **6** compared to MitoTracker. Confocal microscopy of live HeLa cells taken on Leica SP8 X confocal microscope, stained with 1 μM of compounds **6**; (**A**) channel showing emission of **6** (λ_exc_ = 405 nm, λ_em_ = 470–670 nm); (**B**) MitoTracker channel (λ_exc_ = 644 nm, λ_em_ = 665–700 nm); (**C**) overlay of the two channels. Colocalization correlation, PCC values corrected for noise by the RBNCC method for cyanines **1**, **2** and **6**.

Confocal microscopy facilitates the visualization and localization of fluorescent **1–6** inside the cell. All compounds enter the HeLa cells after 60 min incubation at 1 micromolar concentration. Neutral cyanines **1–2** and monocationic dye **6** primarily accumulate in mitochondria [52]. Mitochondrial localization was confirmed using a commercial mitochondrial probe, MitoTracker Deep Red (Figure 8, Figure S46 in SI).

Co-localization with mitotracker was assessed using Pearson’s correlation coefficient (PCC) [53] corrected for noise using replicate-based noise correction correlation (RBNCC) [54] (Figure 8). The results point to significant co-localization of cyanines **1**, **2** and **6** with the commercial mitochondrial tracker (Figure 8).

**Figure 9:**
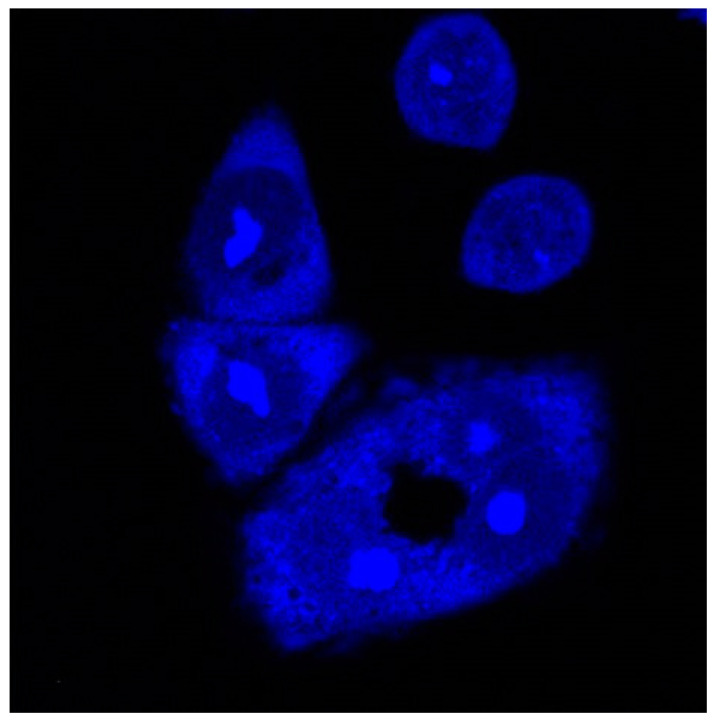
Intracellular distribution of dye **4**. Confocal microscopy of live HeLa cells taken on Leica SP8 X confocal microscope, stained with 1 μM of compounds **4** (λ_exc_ = 405 nm, λ_em_ = 470–670 nm).

The intense fluorescence of compounds **3–5** indicates their accumulation in the nucleus (nucleolus) and partially in the cytoplasm (Figure 9, Figure S47 in SI). This will be further proven in future studies with the appropriate fluorescent nucleus and nucleolus dyes. Recently, thioflavin was proved as a fluorescent marker for nucleolar G-quadruplexes in living cells [55]. The biological study confirms the potential of the tested compounds, especially compounds **3** and **4**, as theranostic agents, possessing both fluorescent properties and cell viability inhibitory effect.

## 5. Conclusions

A detailed characterization of the binding interaction between selenium-substituted cyanine dyes with double-stranded DNA and RNA and G-quadruplex structure is presented here.

Only in titrations of **3** with GC-DNA and **5** with GC-DNA and AU-RNA, sufficient data were collected in the non-linear part of the curve that allowed binding affinity calculation (log *K*_a_ = 5).

Cyanines **3–6** distinguish DNA and RNA helices and different DNA conformations (AT-DNA and GC-DNA), showing diverse ICD profiles. Closer analysis of CD data suggests minor groove binding of **3–6** (either in monomer or aggregated form) to AT-DNA and binding inside major grooves of GC-DNA and AU-RNA. The major groove is the apparent binding site since binding to GC minor groove is sterically hampered by protruding guanine amino groups while the minor groove of AU-RNA is broad and shallow and does not support small molecule binding [31,32].

Compounds **3** and **4** show a 100-fold selectivity for G-quadruplex over duplex DNA in both the Na^+^ and K^+^ solutions (two orders of magnitude higher binding affinities, Table 2 and Table 3, Figures S16, S22 and S23 in SI) [13,47,48]. This may be due to the shorter alkyl substituents on the nitrogen atom of the benzothiazolyl chromophore do not interfere with the dominant G-quadruplex interaction, such as the longer alkyl substituents of the other tested compounds.

According to the analysis of Tel22 measurements in Na^+^ solution, elongated aromatic surfaces of cyanines **1**, **3–4** (and most likely **5–6**), bind to either top/bottom G-tetrad (1:1 stochiometry) or between terminal tetrads of two Tel22 molecules in sandwich-like complex (1:2 stochiometry).

CD spectroscopy analysis of Tel22 in K^+^ solution (positive or bisignate ICD bands) indicates additional binding interaction of **3–6**, possibly with nucleobases/phosphate backbone of grooves or loop bases. Intriguingly, cyanine **6** with thioethyl substituent distinguishes duplex DNA and RNA and G-quadruplex structures by different ICD profiles, bisignate ICD bands of opposite signs. Moreover, it differentiates between Tel22 structures in the K^+^ and Na^+^ solutions by different signs of ICD bisignate bands, the feature that previously was not observed with G-quadruplex targeted probes.

Compounds **3** and **4** with methyl substituents on the benzothiazolyl chromophore show the high cytotoxic effect against all tested tumor cell lines. Colocalization experiments with mitochondrial probe show significant accumulation of cyanines **1**, **2** and **6** in mitochondrial space. Recently, it was reported that G4 structures can form in the mitochondrial genome and possibly affect mitochondrial replication, transcription and translation [56].

Particularly interesting for further research (with rDNA and rRNA G-quadruplex structures [55]) are compounds **3** and **4** showing the submicromolar binding affinity with biologically relevant Tel22 in the K^+^ solution, the localization in nucleus/nucleoli and the selectivity in cell viability inhibition of tumor vs. healthy cell lines. In addition, cyanine **6** with thioethyl substituent is worthy of further investigation due to its significant stabilization effect of Tel22 in K^+^ solution, distinguishing G-quadruplex in K^+^ and Na^+^ solutions, subcellular accumulation in mitochondria and its rather negligible effect on cell viability.

## Figures and Tables

**Table 1:** Electronic absorption data of **1–6** compounds (sodium cacodylate buffer, *I* = 0.05 mol dm^−3^, pH = 7.0).

Compd	UV/Vis λ_max_ (nm)	ε × 10^3^/mmol^−1^ cm^2^
**1**	425	76.7
**2**	431	37.6
**3**	423	69.7
**4**	427	55.2
**5**	406	42.0
**6**	407	30.6

**Table 2:** Binding constants (log*K*_a_) ^a^ for 1:1 and 1:1/1:2 complexes of **1–6**-Tel22 calculated from the fluorescence titrations and ∆*T*_m_^d^ values (°C) of Tel22 upon addition of ratio ^e^ ***r*** = 1.0 of **1–6** (sodium cacodylate buffer, pH = 7.0, *I* = 0.1 mol dm^−3^).

Compound	log*K*_a1_; log*K*_a2_ ^b^	∆*T*_m_/°C
**1**	6.93 ± 0.25; 5.69 ± 0.23	0
**2**	- ^c^	0
**3**	7.26 ± 0.41; 5.93 ± 0.47	0.5
**4**	6.19 ± 0.01	2.1
**5**	- ^c^	2.1
**6**	- ^c^	2.0

^a^ Stability constant log *K*_a_ and stoichiometry calculated by processing the titration data using SPECFIT program [46]. ^b^ *K*_a1_ and *K*_a2_ refer to the equilibria L + G ⇆ LG and LG + G ⇆ LG_2_ (L = ligand, G = G-quadruplex), respectively. ^c^ small fluorescence change/insufficient data points in non-linear part of curve disabled accurate calculation of association constant. ^d^ Error in ∆*T*_m_: ± 0.5 °C. ^e^ *r* = [compound]/[polynucleotide].

**Table 3:** Binding constants (log*K*_a_) ^a^ for 1:1 complexes of **1–6**-Tel22 calculated from the fluorescence titrations and ∆*T*_m_ ^e^ values (°C) of Tel22 upon addition of ratio ^f^ ***r*** = 1.0 of 1–6. (potassium phosphate buffer, pH = 7.0, I = 0.1 mol dm^−3^).

Compound	logK_a_ ^b^	∆*T*_m_/°C
**1**	- ^c^	- ^d^
**2**	- ^d^	- ^d^
**3**	6.88 ± 0.05	0.5
**4**	7.78 ± 0.18	3.2
**5**	- ^c^	5.1
**6**	- ^c^	6.9

^a^ Stability constant log Ka and stoichiometry calculated by processing the titration data SPECFIT program [46]. ^b^ Ka refer to the equilibrium L + G ⇆ LG (L = ligand, G = G-quadruplex). ^c^ small fluorescence change/insufficient data points in non-linear part of curve disabled accurate calculation of association constant. ^d^ not determined due to linear changes in titration with Tel22 in sodium cacodylate buffer or potassium phosphate buffer. ^e^ Error in ∆Tm: ±0.5 °C. ^f^ r = [compound]/[polynucleotide].

**Table 4:** The cytotoxic activity of analyzed compounds ^a^ towards HeLa, MDA-MB-435S, SK-OV-3 cells and fibroblasts. IC_50_ (IC_50_/µM ± SD) values were calculated upon 72 h of incubation with the compounds. The cytotoxicity was measured by MTT assay.

	1	3	4	5	6
HeLa	31.856 ± 1.142	1.025 ± 0.672	0.793 ± 0.157	27.917 ± 4.141	30.667 ± 4.072
MDA-MB-435S	18.525 ± 1.226	0.987 ± 0.177	0.6135 ± 0.0776	37.115 ± 3.021	32.767 ± 3.756
SK-OV-3	32.75 ± 1.09	0.548 ± 0.102	1.319 ± 0.125	29.5625 ± 5.724	>40
Fibroblasts	>40	2.375 ± 0.161	>2.5	>40	>40

^a^ IC_50_ for compound **2** was not determined due to aggregation.

## Data Availability

Not applicable.

## Supplementary Materials

The following supporting information can be downloaded at: https://www.mdpi.com/article/10.3390/biom13010128/s1.
