# Supplementary material for: Selenium-Substituted Monomethine Cyanine Dyes as Selective G-Quadruplex Spectroscopic Probes with Theranostic Potential
Source: Biomolecules. 2023 Jan 7;13(1):128. doi: 10.3390/biom13010128 (PMC9856044; doi:10.3390/biom13010128)
Supplement: Supplementary file 1 [file biomolecules-13-00128-s001.zip › biomolecules-2101007-supplementary.pdf]

Supporting Information

Selenium-substituted monomethine cyanine dyes as selective G-quadruplex spectroscopic probes with theranostic potential

Ivana Fabijanić,[1] Atanas Kurutos,[2] Ana Tomašić Paić,[1] Tadić Vanja,[3] Fadhil S. Kamounah,[4] Lucija Horvat,[3] Anamaria Brozovic,[3] Ivo Crnolatac,[1] Marijana Radić Stojković [1*]

[1] *Division of Organic Chemistry and Biochemistry, Ruđer Bošković Institute, Bijenička cesta 54, 10000 Zagreb, Croatia*

[2] *Institute of Organic Chemistry with Centre of Phytochemistry, Bulgarian Academy of Sciences Acad. G. Bonchev str., bl. 9, Sofia 1113, Bulgaria*

[3] *Division of Molecular Biology, Ruđer Bošković Institute, Bijenička cesta 54, 10000 Zagreb, Croatia*

[4] *University of Copenhagen, Department of Chemistry, Universitetsparken 5, DK-2100, Copenhagen, Denmark*

*Corresponding author

Division of Organic Chemistry and Biochemistry, Laboratory for Biomolecular Interactions and Spectroscopy, Ruđer Bošković Institute, Bijenička cesta 54, 10000 Zagreb, Croatia, Tel: +38514571220; Fax:+38514680195, email: mradic@irb.hr

Contents

1. Spectroscopic characterization of **1 – 6** compounds in aqueous solutions.
2. Interactions of **1 – 6** compounds with ds-polynucleotides in neutral medium (pH=7.0)
2.1. Fluorimetric titrations
2.2. Thermal melting experiments
2.3. Circular dichroism (CD) titrations
3. Interactions of **1 – 6** compounds with Tel22 in sodium cacodylate buffer (pH=7.0)
3.1. Fluorimetric titrations
3.2. Thermal melting experiments
3.3. Circular dichroism (CD) titrations
4. Interactions of **1** and **3 - 6** with Tel22 in potassium phosphate buffer (pH=7.0)
4.1. Fluorimetric titrations
4.2. Circular dichroism (CD) titrations
4.3. Thermal melting experiments
5. Confocal microscopy
6. HRMS and NMR spectra

1. Spectroscopic characterization of 1 – 6 compounds in aqueous solutions

All experiments were done by adding small aliquots of DMSO stock solutions of compounds **1-6** ($c = 8 \times 10^{-4}$ mol dm^{-3}) to buffer solutions (0.05 M Na cacodylate).

Absorption maxima and corresponding molar extinction coefficients (ε) are given in Table S1 and Figures S1 – S6.

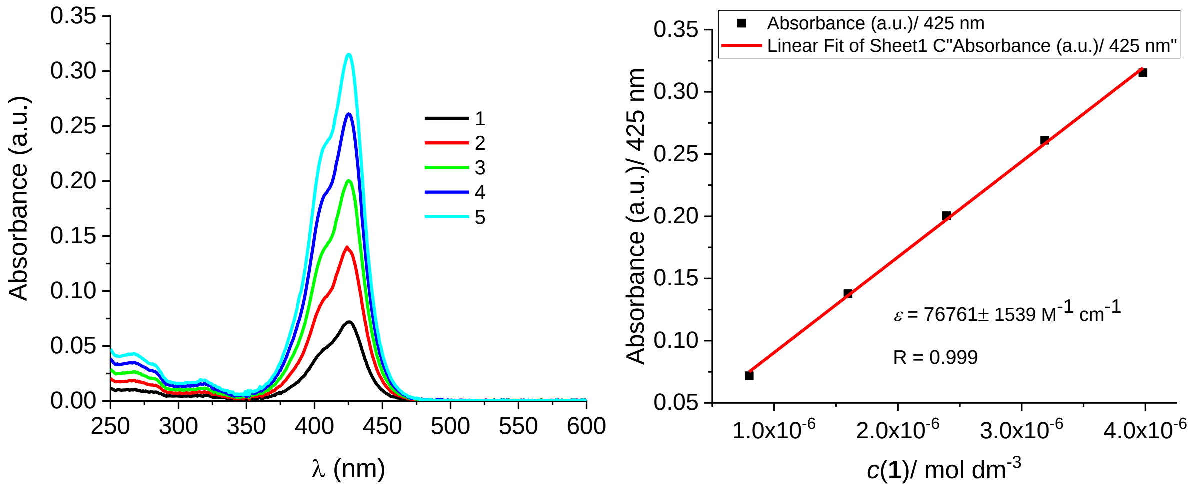

a) b)

Figure S1. a) UV/Vis spectra changes of **1** at different concentrations (concentration range from 7.99×10^{-7} to 3.98×10^{-6} mol dm^{-3}) at pH=7, sodium cacodylate buffer, I=0.05 M; b) with corresponding calibration line.

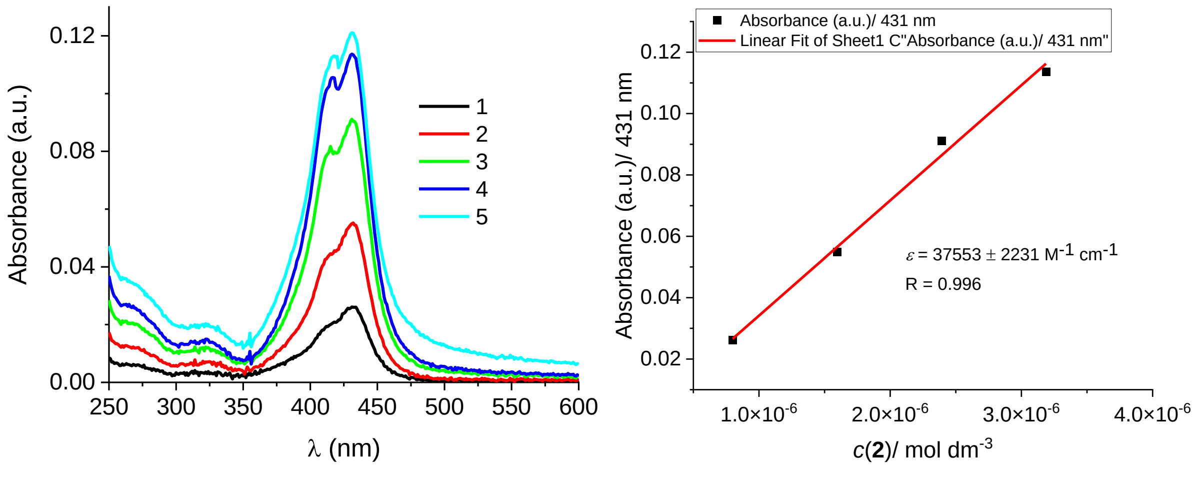

a) b)

Figure S2. UV/Vis spectra changes of **2** at different concentrations (concentration range from 7.99×10^{-7} to 3.19×10^{-6} mol dm^{-3}) at pH=7, sodium cacodylate buffer, I=0.05 M; b) with corresponding calibration line.

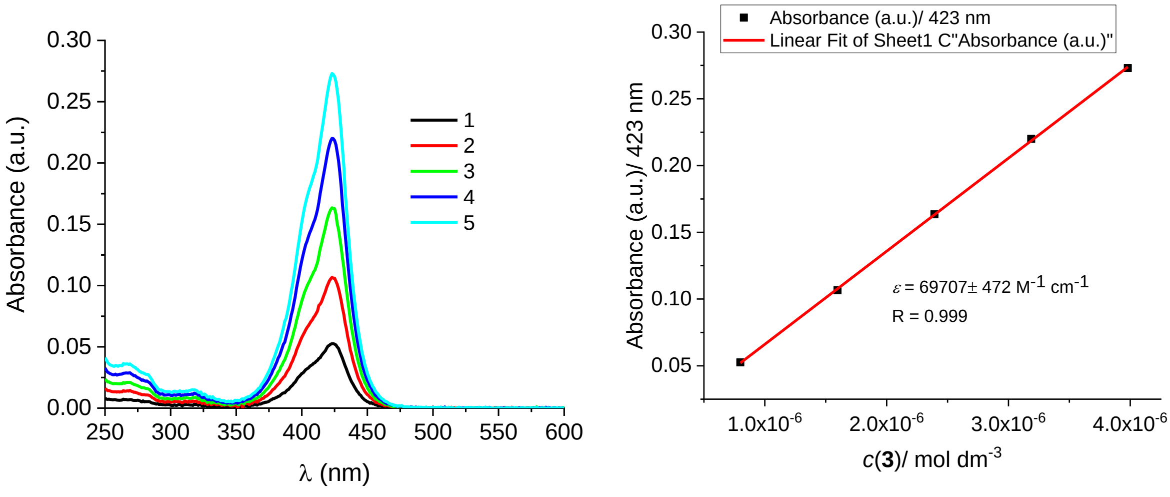

a) b)

Figure S3. UV/Vis spectra changes of **3** at different concentrations (concentration range from 7.99×10^{-7} to 3.98×10^{-6} mol dm^{-3}) at pH=7, sodium cacodylate buffer, I=0.05 M; b) with corresponding calibration line.

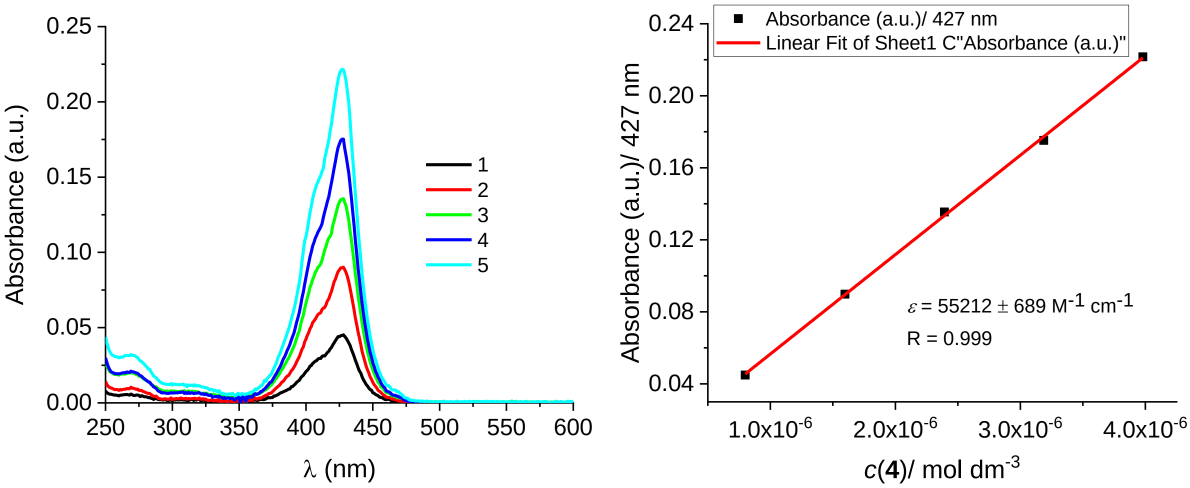

a) b)

Figure S4. UV/Vis spectra changes of **4** at different concentrations (concentration range from 7.99×10^{-7} to 3.98×10^{-6} mol dm^{-3}) at pH=7, sodium cacodylate buffer, I=0.05 M; b) with corresponding calibration line.

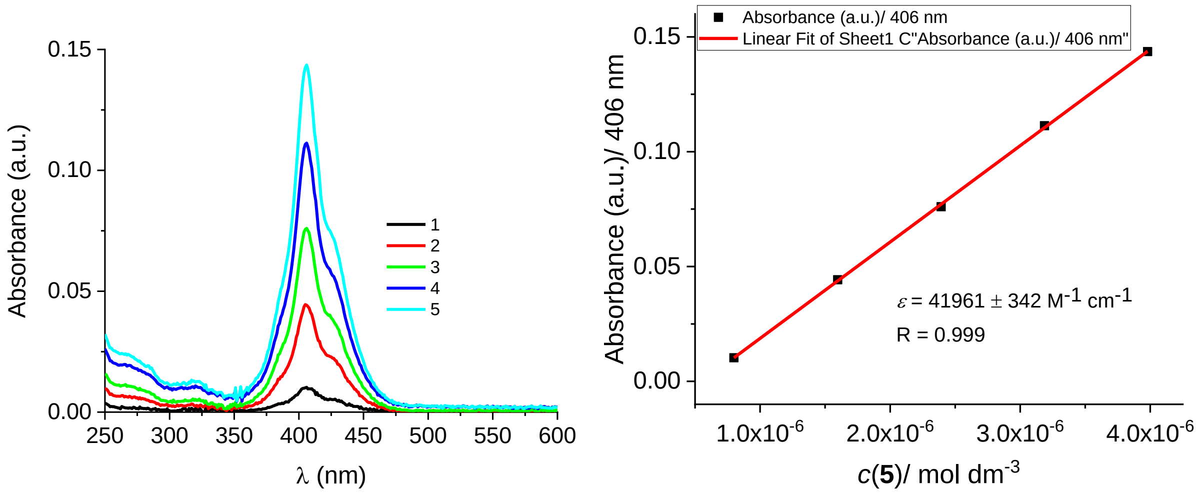

a) b)

Figure S5. UV/Vis spectra changes of **5** at different concentrations (concentration range from 7.99×10^{-7} to 3.98×10^{-6} mol dm^{-3}) at pH=7, sodium cacodylate buffer, I=0.05 M; b) with corresponding calibration line.

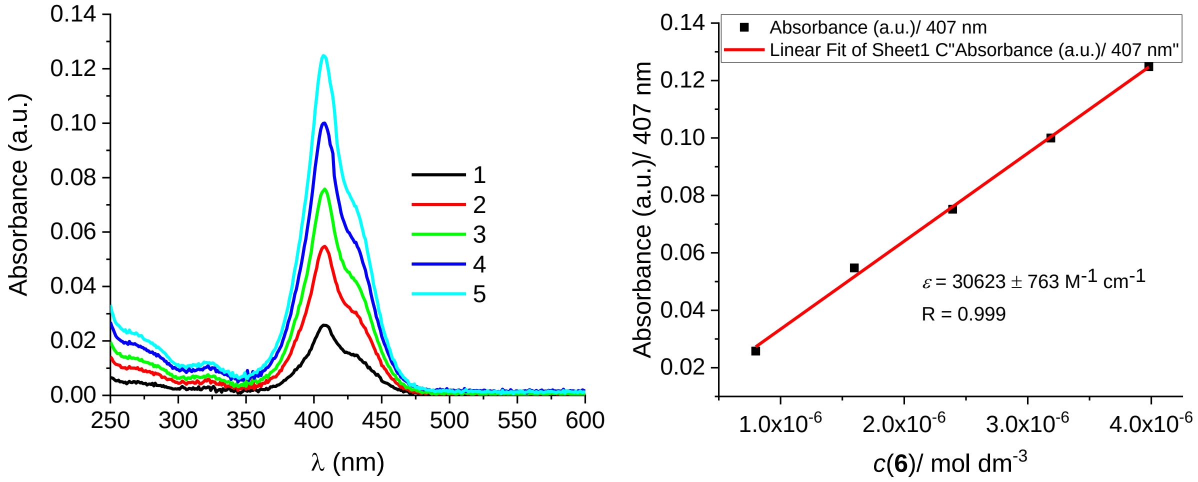

a) b)

Figure S6 UV/Vis spectra changes of **6** at different concentrations (concentration range from 7.99×10^{-7} to 3.98×10^{-6} mol dm^{-3}) at pH=7, sodium cacodylate buffer, I=0.05 M; b) with corresponding calibration line.

Table S1. Electronic absorption data of **1 – 6**.

	**pH = 7,0**[a]	
	**λ_{max} / nm**	**$\varepsilon \times 10^3$ / mol^{-1} cm^2**
**1**	425	76.7
**2**	431	37.6
**3**	423	69.7
**4**	427	55.2
**5**	406	42.0
**6**	407	30.6

[a] Sodium cacodylate buffer, I = 0,05 mol dm^{-3}, pH = 7,0.

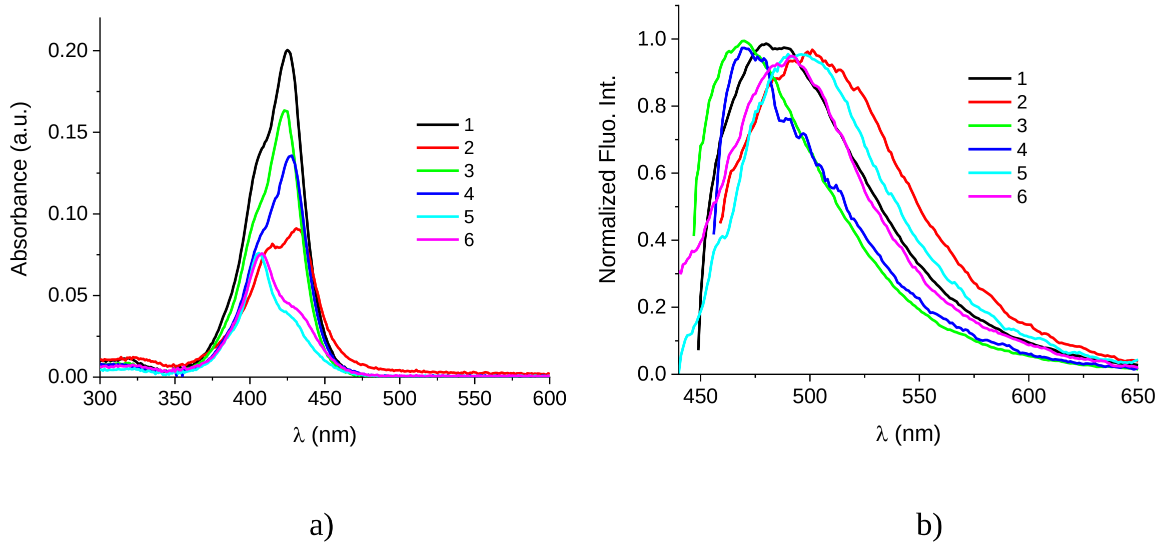

Figure S7. a) UV Vis spectra of 1 to 6 at c = 2.39× 10^{-7} mol dm^{-3}, at pH=7, Sodium cacodylate buffer, I=0.05 mol dm^{-3}; **b)** Normalized spectra of **1** (λ_{exc}= 425 nm), **2** (λ_{exc}= 431 nm), **3** (λ_{exc}= 423 nm), **4** (λ_{exc}= 427 nm), **5** (λ_{exc} = 406 nm) and **6** (λ_{exc}= 407 nm), S_{exc}=20, S_{em} = 20, at pH=7, sodium cacodylate buffer, I=0.05 mol dm^{-3}.

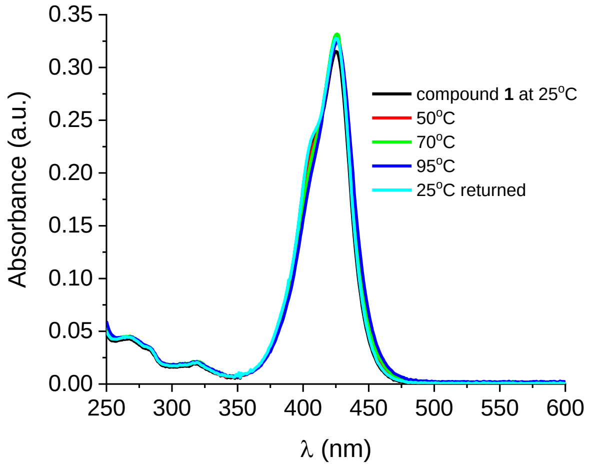

a)

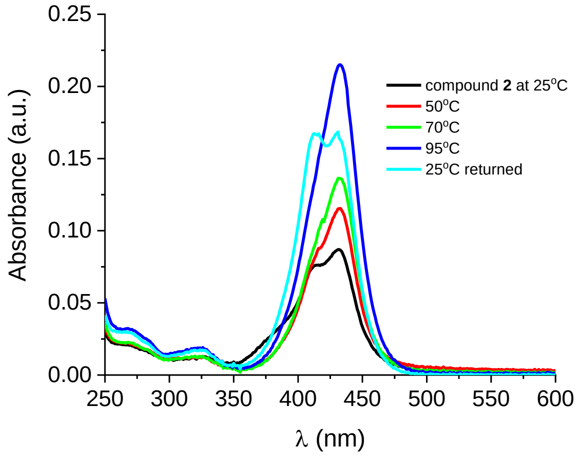

b)

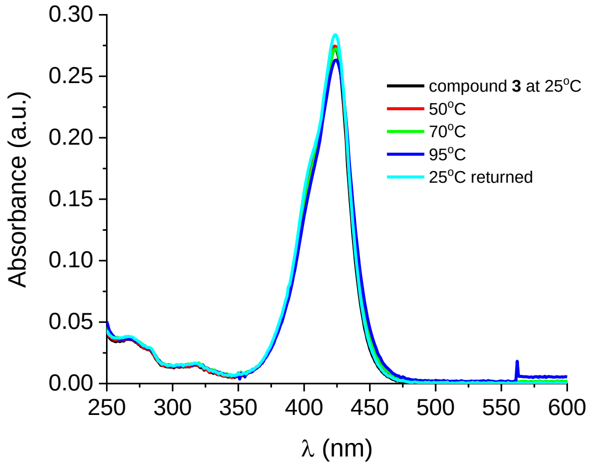

c)

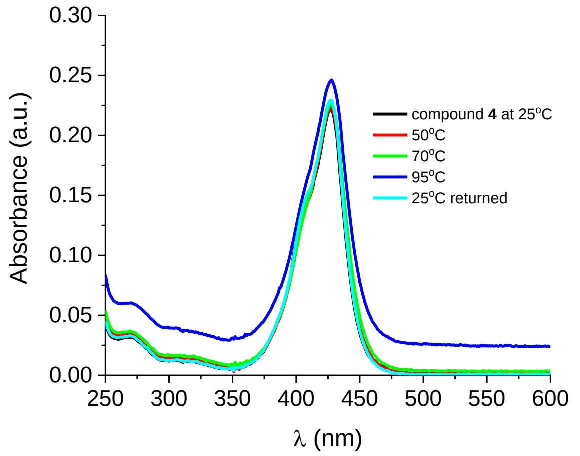

d)

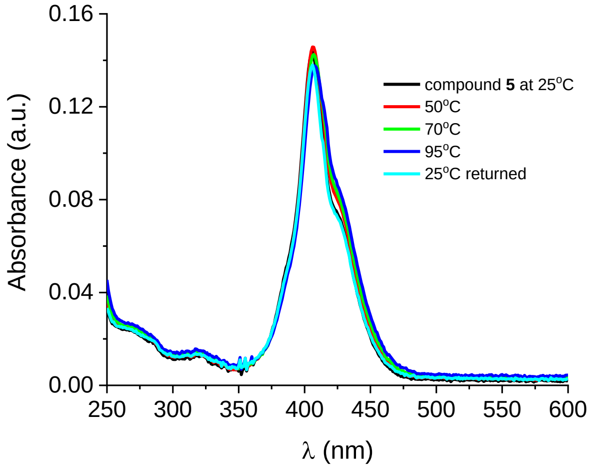

e)

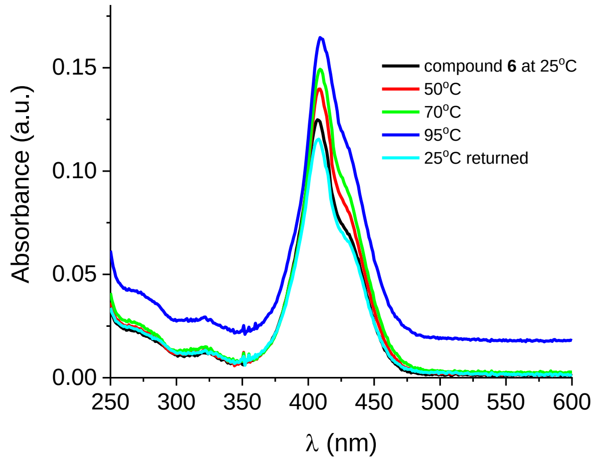

f)

Figure S8. Changes in UV Vis spectra upon temperature change of a) **1** (c = 3.98×10^{-6} mol dm^{-3}); b) **2** (c = 3.19×10^{-6} mol dm^{-3}); c) **3** (c = 3.98×10^{-6} mol dm^{-3}); d) **4** (c = 3.98×10^{-6} mol dm^{-3}); e) **5** (c = 3.98×10^{-6} mol dm^{-3}); f) **6** (c = 3.98×10^{-6} mol dm^{-3}). All measurements were done at pH=7 in sodium cacodylate buffer, I=0.05 mol dm^{-3}.

2. Interactions of 1 – 6 with ds-polynucleotides in neutral medium (pH=7.0)

2.1. Fluorimetric titrations

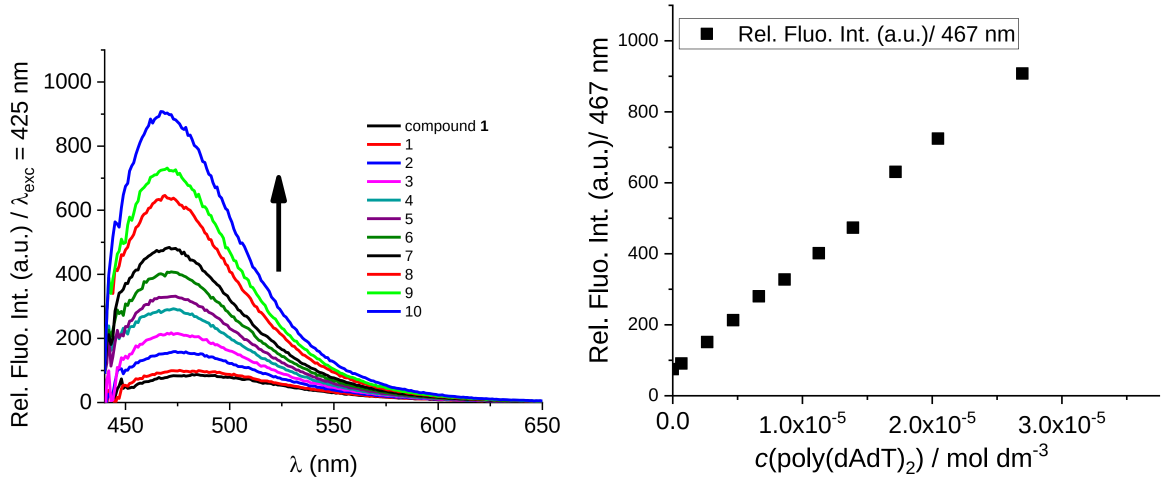

a) b)

Figure S9. a) Changes in fluorescence spectrum of **1** (c= 5.0 × 10^{-7} mol dm^{-3}, λ_{exc}=425 nm) upon titration with poly$(dAdT)_2$ (c = 6.66 × 10^{-6} –2.69 × 10^{-5} mol dm^{-3}), S_{exc} = 20, S_{em} = 20; b) Fluorescence intensities of **1** at λ_{max} = 467 nm upon addition of poly$(dAdT)_2$, at pH=7.0, sodium cacodylate buffer, I = 0.05 mol dm^{-3}.

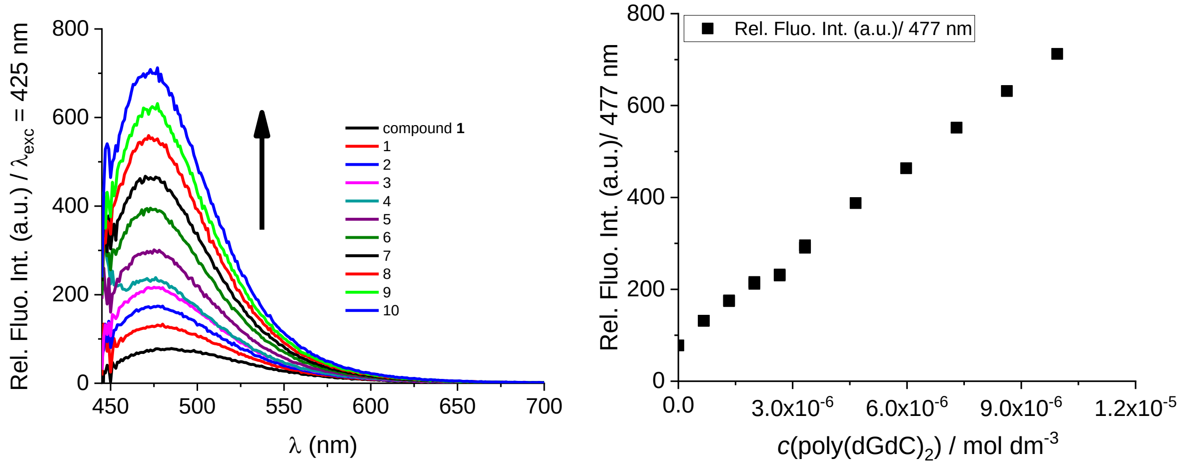

a) b)

Figure S10. a) Changes in fluorescence spectrum of **1** (c= 5.0 × 10^{-7} mol dm^{-3}, λ_{exc}=425 nm) upon titration with poly$(dGdC)_2$ (c = 6.66 × 10^{-6} –9.94 × 10^{-6} mol dm^{-3}), S_{exc} = 20, S_{em} = 20; b) Fluorescence intensities of **1** at λ_{max} = 467 nm upon addition of poly$(dGdC)_2$, at pH=7.0, sodium cacodylate buffer, I = 0.05 mol dm^{-3}.

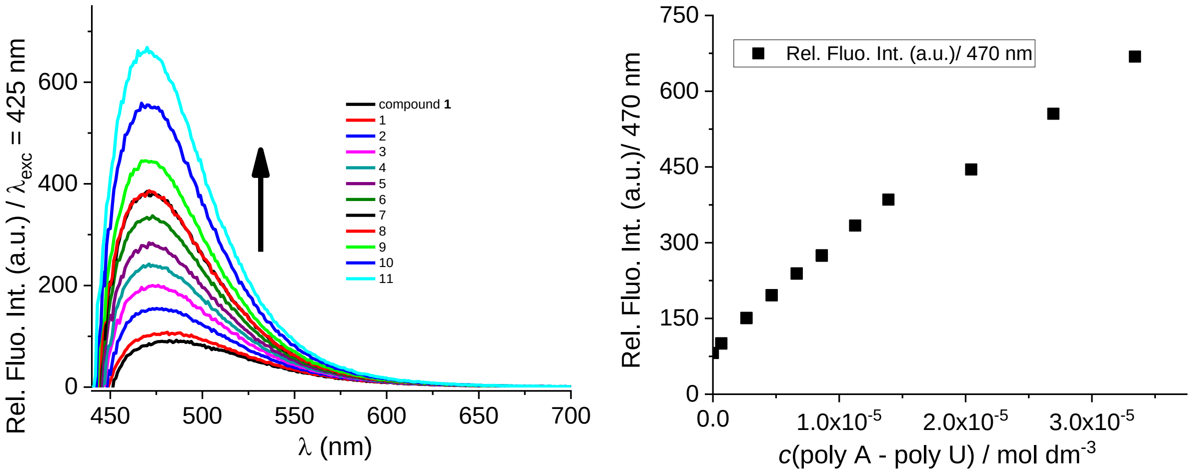

a) b)

Figure S11. a) Changes in fluorescence spectrum of **1** (c= 5.0 × 10^{-7} mol dm^{-3}, λ_{exc}=425 nm) upon titration with poly A – poly U (c = 6.66 × 10^{-6} –3.34 × 10^{-5} mol dm^{-3}), S_{exc} = 20, S_{em} = 20; b) Fluorescence intensities of **1** at λ_{max} = 467 nm upon addition of poly A – poly U, at pH=7.0, sodium cacodylate buffer, I = 0.05 mol dm^{-3}.

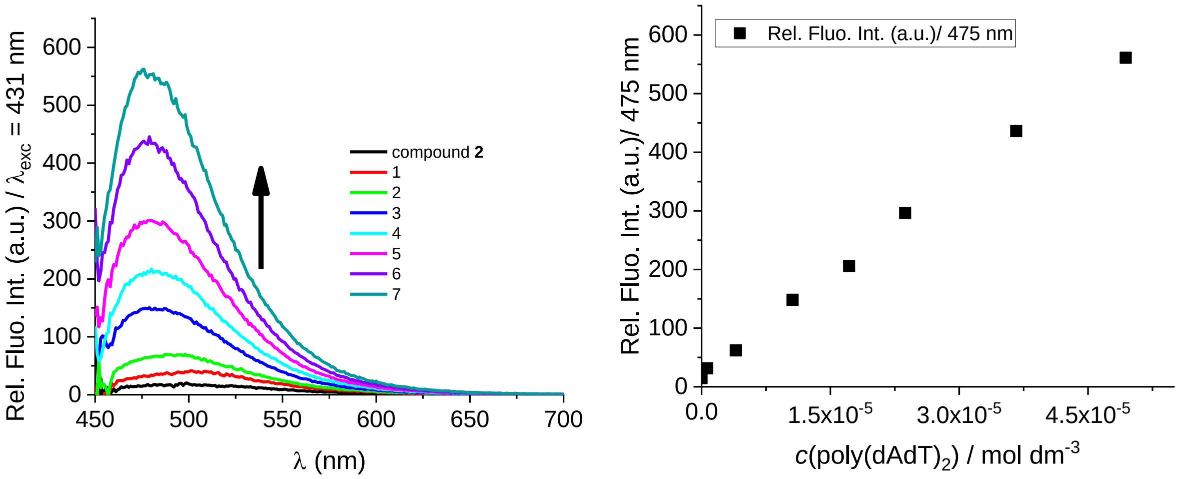

a) b)

Figure S12. a) Changes in fluorescence spectrum of **2** (c= 2.5 × 10^{-7} mol dm^{-3}, λ_{exc}= 431 nm) upon titration with poly(dAdT)$_2$ (c = 6.66 × 10^{-6} –4.94 × 10^{-5} mol dm^{-3}), S_{exc} = 20, S_{em} = 20; b) Fluorescence intensities of **2** at λ_{max} = 475 nm upon addition of poly(dAdT)$_2$, at pH=7.0, sodium cacodylate buffer, I = 0.05 mol dm^{-3}.

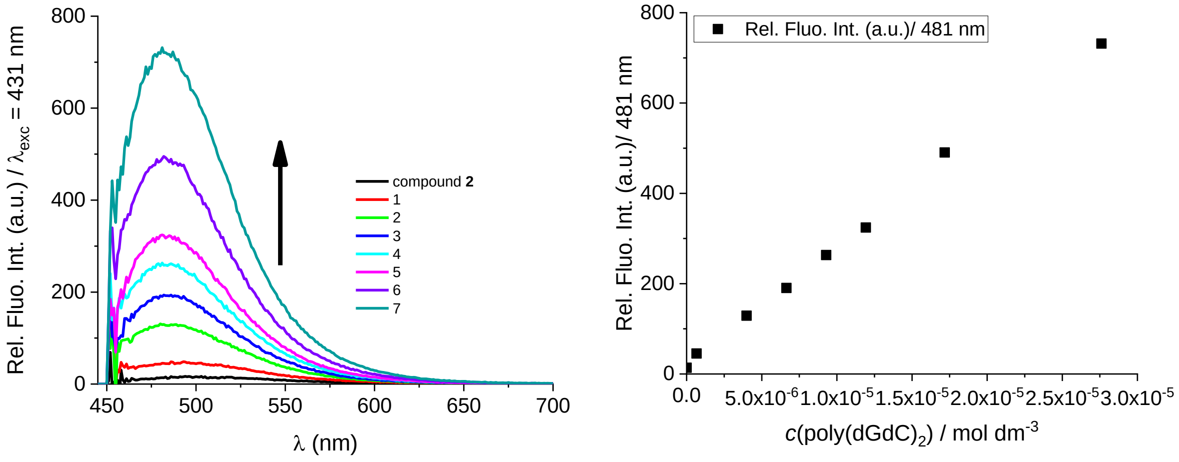

a) b)

Figure S13. a) Changes in fluorescence spectrum of **2** (c= 2.5 × 10^{-7} mol dm^{-3}, λ_{exc}=431 nm) upon titration with poly(dGdC)$_2$ (c = 6.66 × 10^{-6} – 2.76 × 10^{-5} mol dm^{-3}), S_{exc} = 20, S_{em} = 20; b) Fluorescence intensities of **2** at λ_{max} = 481 nm upon addition of poly(dGdC)$_2$, at pH=7.0, sodium cacodylate buffer, I = 0.05 mol dm^{-3}.

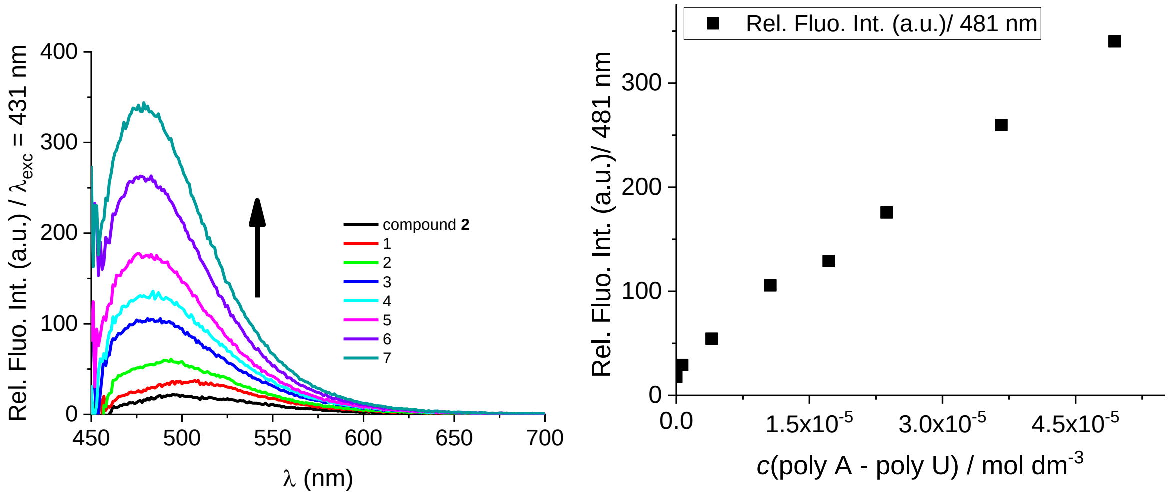

a) b)

Figure S14. a) Changes in fluorescence spectrum of **2** (c = 2.5 × 10^{-76} mol dm^{-3}, λ_{exc}=431 nm) upon titration with poly A – poly U (c = 6.66 × 10^{-6} –4.94 × 10^{-5} mol dm^{-3}), S_{exc} = 20, S_{em} = 20; b) Fluorescence intensities of **2** at λ_{max} = 481 nm upon addition of poly A – poly U, at pH=7.0, sodium cacodylate buffer, I = 0.05 mol dm^{-3}.

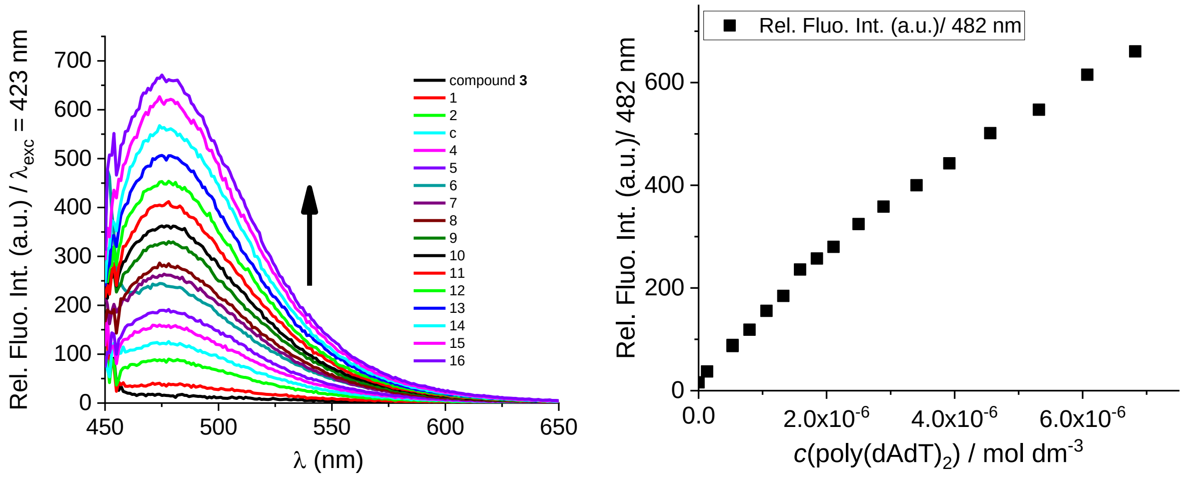

a) b)

Figure S15. a) Changes in fluorescence spectrum of **3** (c= 5.0 × 10^{-8} mol dm^{-3}, λ_{exc}=423 nm) upon titration with poly$(dAdT)_2$ (c= 1.33 × 10^{-7} – 6.82 × 10^{-6} mol dm^{-3}), S_{exc} = 20, S_{em} = 20; b) Fluorescence intensities of **3** at λ_{max} = 482 nm upon addition of poly$(dAdT)_2$, at pH=7.0, sodium cacodylate buffer, I = 0.05 mol dm^{-3}.

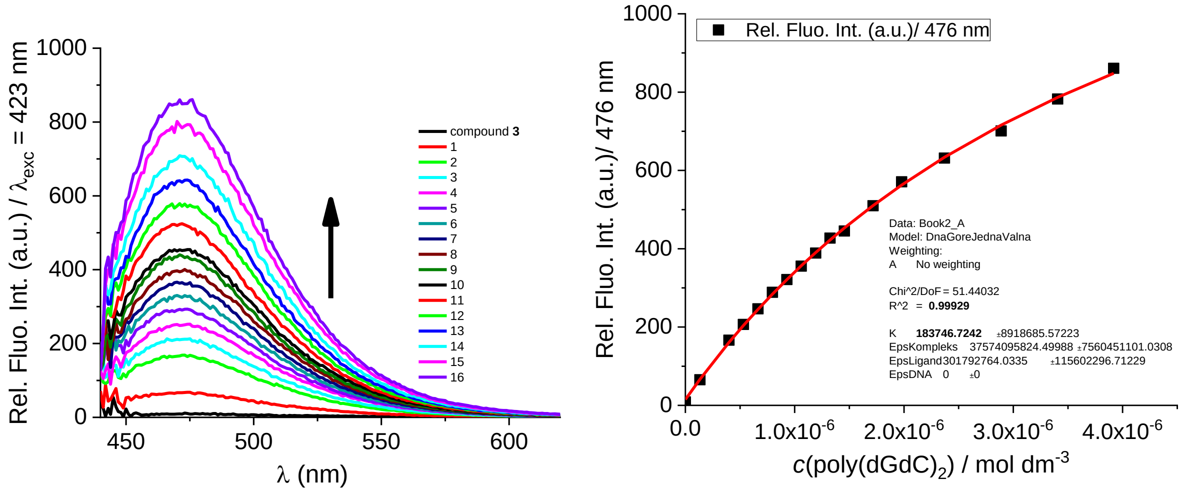

a) b)

Figure S16. a) Changes in fluorescence spectrum of **3** (c= 5.0 × 10^{-8} mol dm^{-3}, λ_{exc}=423 nm) upon titration with poly$(dGdC)_2$ (c= 1.33 × 10^{-7} – 3.92 × 10^{-6} mol dm^{-3}), S_{exc} = 20, S_{em} = 20; **b)** Experimental (■) and calculated (—) fluorescence intensities of **3** at λ_{max} = 476 nm upon addition of poly$(dGdC)_2$, at pH=7.0, sodium cacodylate buffer, I = 0.05 mol dm^{-3}.

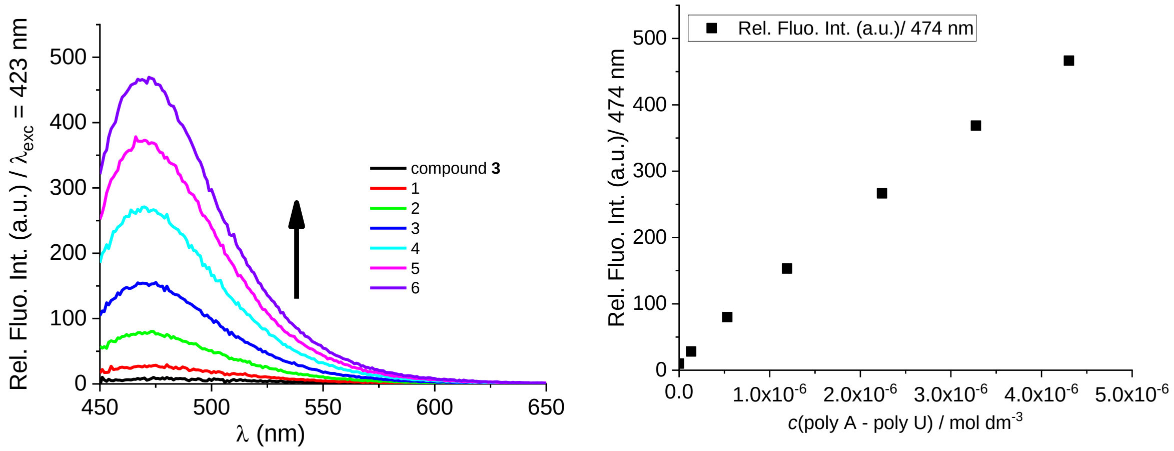

a) b)

Figure S17. a) Changes in fluorescence spectrum of **3** (c= 5.0 × 10^{-8} mol dm^{-3}, λ_{exc}=423 nm) upon titration with poly A – poly U (c= 1.33 × 10^{-7} – 4.30 × 10^{-6} mol dm^{-3}) S_{exc} = 20, S_{em} = 20; b) Fluorescence intensities of **3** at λ_{max} = 474 nm upon addition of poly A – poly U, at pH=7.0, sodium cacodylate buffer, I = 0.05 mol dm^{-3}.

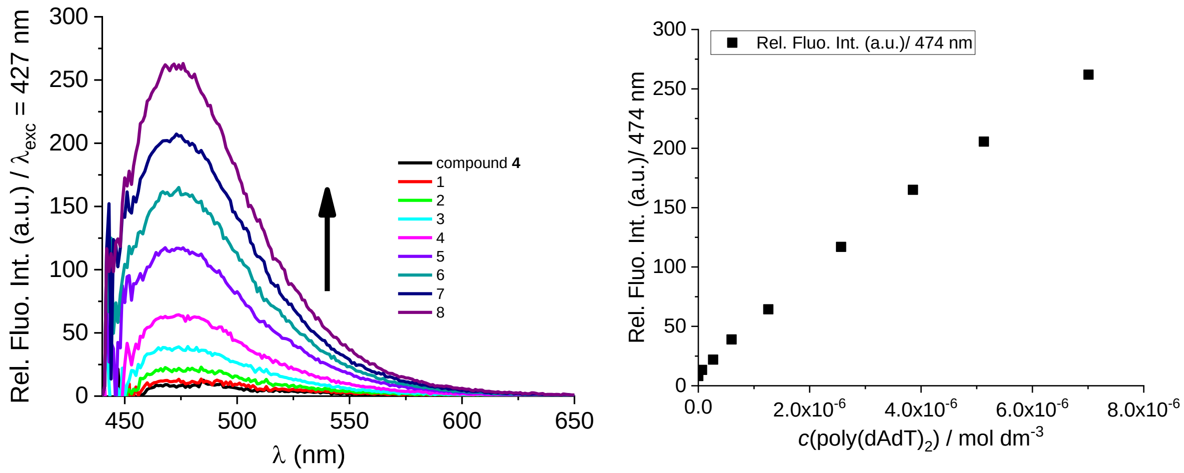

a) b)

Figure S18. a) Changes in fluorescence spectrum of **4** (c= 5.0 × 10^{-8} mol dm^{-3}, λ_{exc}= 427 nm) upon titration with poly(dAdT)$_2$ (c= 6.66 × 10^{-8} – 7.00 × 10^{-6} mol dm^{-3}), S_{exc} = 20, S_{em} = 20; b) Fluorescence intensities of **4** at λ_{max} = 474 nm upon addition of poly(dAdT)$_2$, at pH=7.0, sodium cacodylate buffer, I = 0.05 mol dm^{-3}.

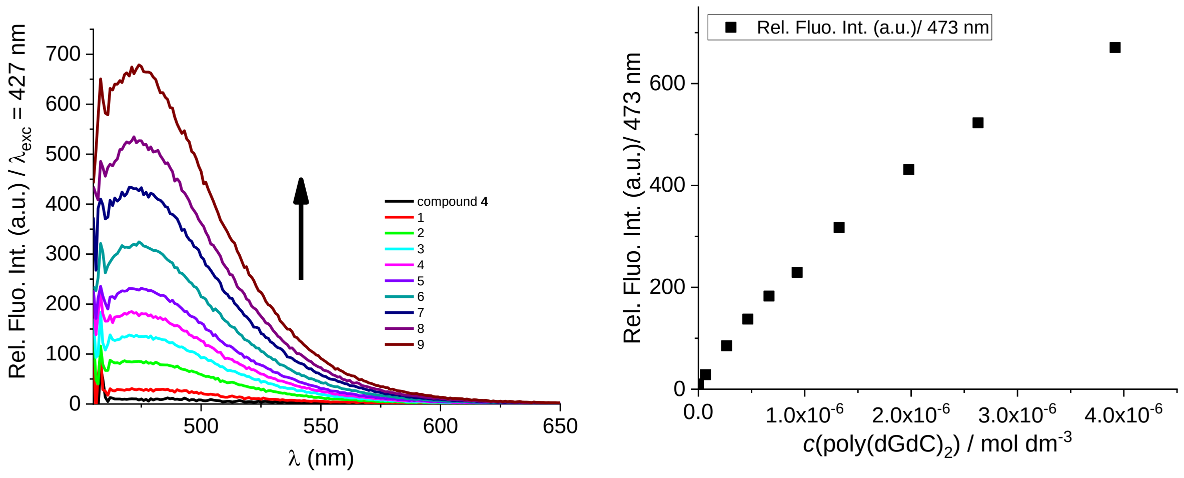

a) b)

Figure S19. a) Changes in fluorescence spectrum of **4** (c= 5.0 × 10^{-8} mol dm^{-3}, λ_{exc}= 427 nm) upon titration with poly(dGdC)$_2$ (c= 6.66 × 10^{-8} – 3.92 × 10^{-6} mol dm^{-3}), S_{exc} = 20, S_{em} = 20; b) Fluorescence intensities of **4** at λ_{max} = 473 nm upon addition of poly(dGdC)$_2$, at pH=7.0, sodium cacodylate buffer, I = 0.05 mol dm^{-3}.

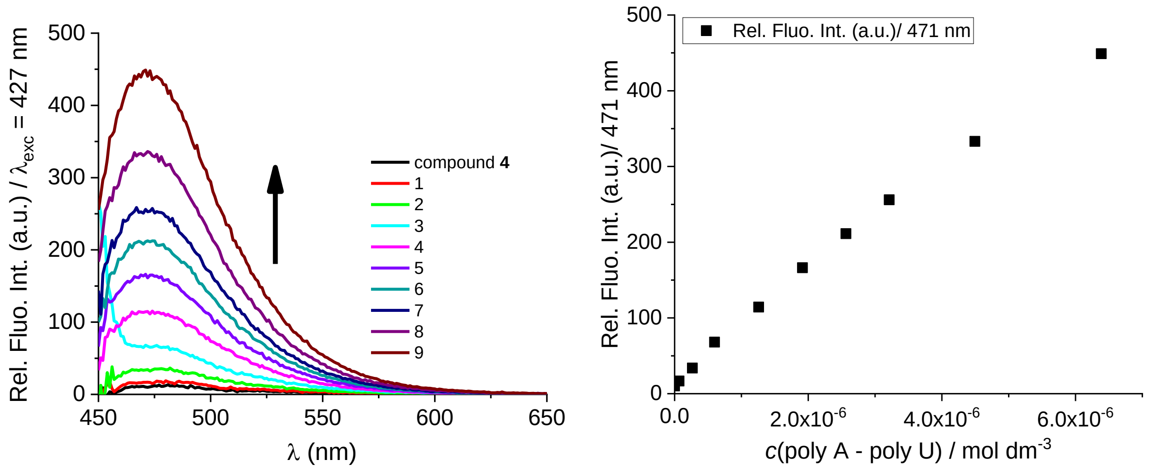

a) b)

Figure S20. a) Changes in fluorescence spectrum of **4** (c= 5.0 × 10^{-8} mol dm^{-3}, λ_{exc}= 427 nm) upon titration with poly A – poly U (c= 6.66 × 10^{-8} – 6.39 × 10^{-6} mol dm^{-3}) S_{exc} = 20, S_{em} = 20; b) Fluorescence intensities of **4** at λ_{max} = 471 nm upon addition of poly A – poly U, at pH=7.0, sodium cacodylate buffer, I = 0.05 mol dm^{-3}.

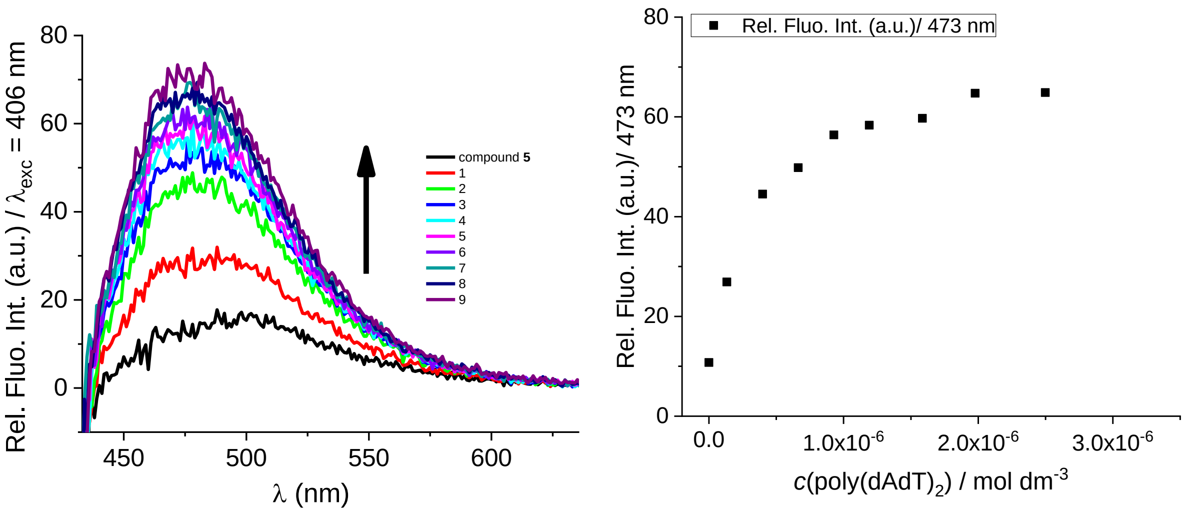

a) b)

Figure S21. a) Changes in fluorescence spectrum of **5** (c= 1.0 × 10^{-7} mol dm^{-3}, λ_{exc}= 406 nm) upon titration with $poly(dAdT)_2$ (c= 1.33 × 10^{-7} – 2.50 × 10^{-6} mol dm^{-3}), S_{exc} = 20, S_{em} = 20; b) Fluorescence intensities of **5** at λ_{max} = 473 nm upon addition of $poly(dAdT)_2$, at pH=7.0, sodium cacodylate buffer, I = 0.05 mol dm^{-3}.

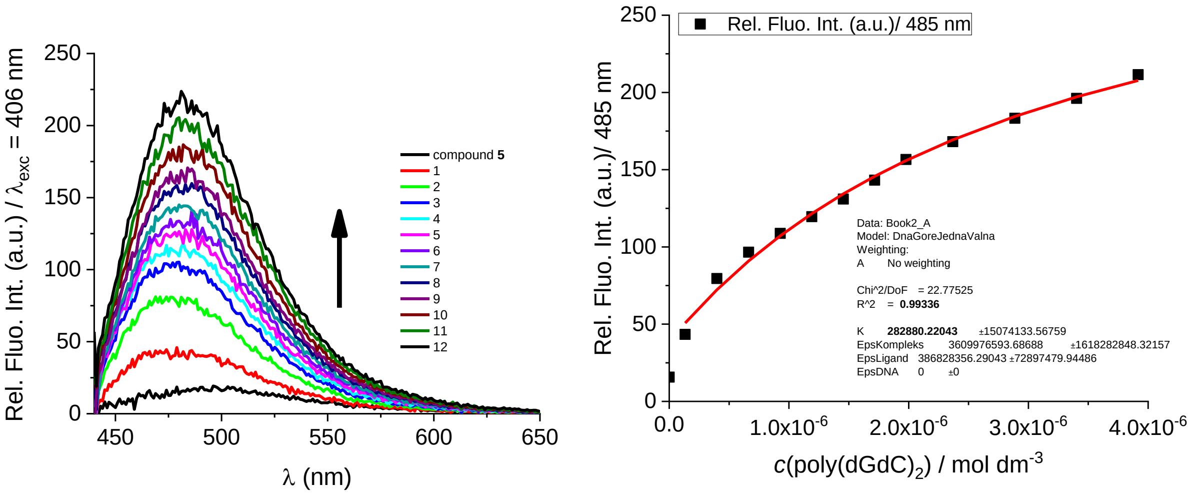

a) b)

Figure S22. a) Changes in fluorescence spectrum of **5** (c= 1.0 × 10^{-7} mol dm^{-3}, λ_{exc}= 406 nm) upon titration with $poly(dGdC)_2$ (c= 1.33 × 10^{-7} – 3.92 × 10^{-6} mol dm^{-3}), S_{exc} = 20, S_{em} = 20; b) Experimental (■) and calculated (—) fluorescence intensities of **5** at λ_{max} = 485 nm upon addition of $poly(dGdC)_2$, at pH=7.0, sodium cacodylate buffer, I = 0.05 mol dm^{-3}.

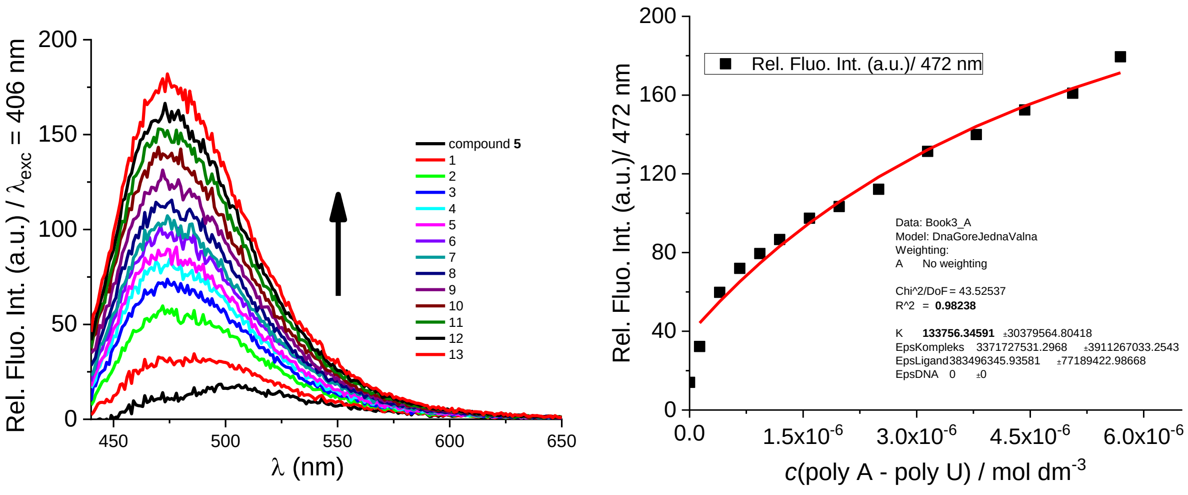

a) b)

Figure S23. a) Changes in fluorescence spectrum of **5** (c= 1.0 × 10^{-7} mol dm^{-3}, λ_{exc}= 406 nm) upon titration with poly A – poly U (c= 1.33 × 10^{-7} – 5.69 × 10^{-6} mol dm^{-3}), S_{exc} = 20, S_{em} = 20; b) Experimental (■) and calculated (—) fluorescence intensities of **5** at λ_{max} = 472 nm upon addition of poly A – poly U, at pH=7.0, sodium cacodylate buffer, I = 0.05 mol dm^{-3}.

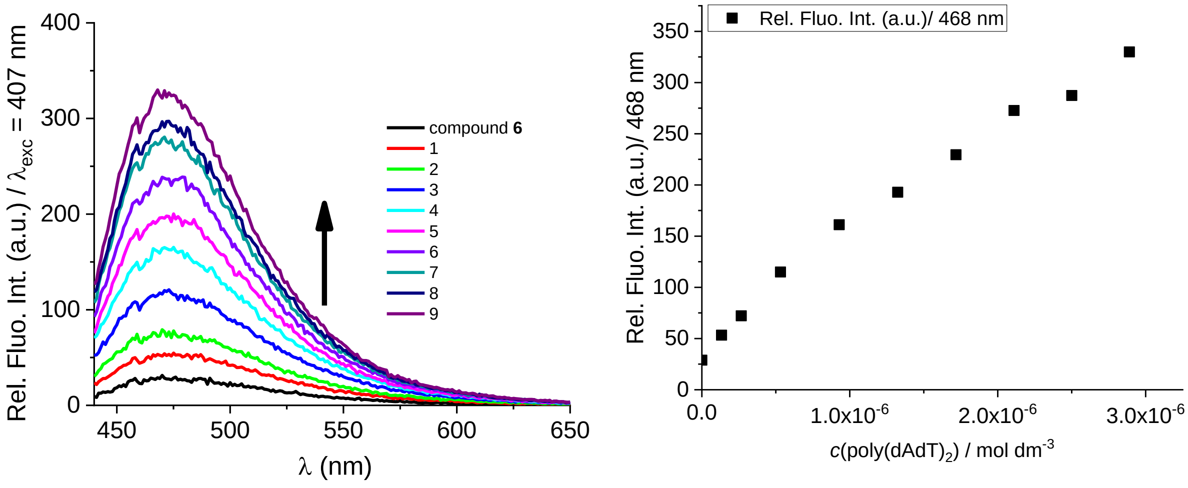

a) b)

Figure S24. a) Changes in fluorescence spectrum of **6** (c= 2.5 × 10^{-7} mol dm^{-3}, λ_{exc}= 407 nm) upon titration with poly(dAdT)$_2$ (c= 1.33 × 10^{-7} – 2.89 × 10^{-6} mol dm^{-3}), S_{exc} = 20, S_{em} = 20; b) Fluorescence intensities of **6** at λ_{max} = 468 nm upon addition of poly(dAdT)$_2$, at pH=7.0, sodium cacodylate buffer, I = 0.05 mol dm^{-3}.

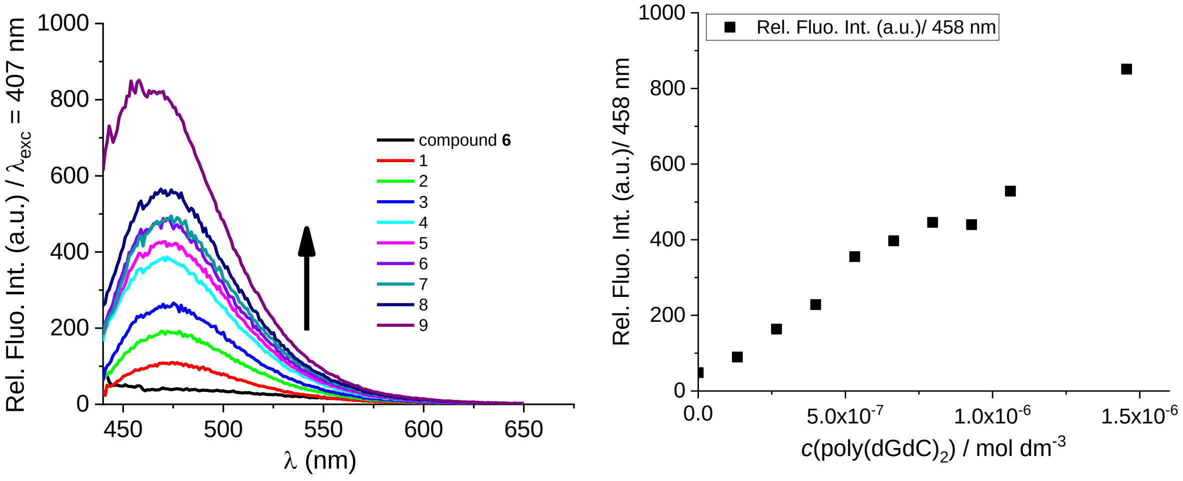

a) b)

Figure S25. a) Changes in fluorescence spectrum of **6** (c= 2.5 × 10^{-7} mol dm^{-3}, λ_{exc}= 407 nm) upon titration with poly(dGdC)$_2$ (c= 1.33 × 10^{-7} – 1.45 × 10^{-6} mol dm^{-3}), S_{exc} = 20, S_{em} = 20; b) Fluorescence intensities of **6** at λ_{max} = 458 nm upon addition of poly(dGdC)$_2$, at pH=7.0, sodium cacodylate buffer, I = 0.05 mol dm^{-3}.

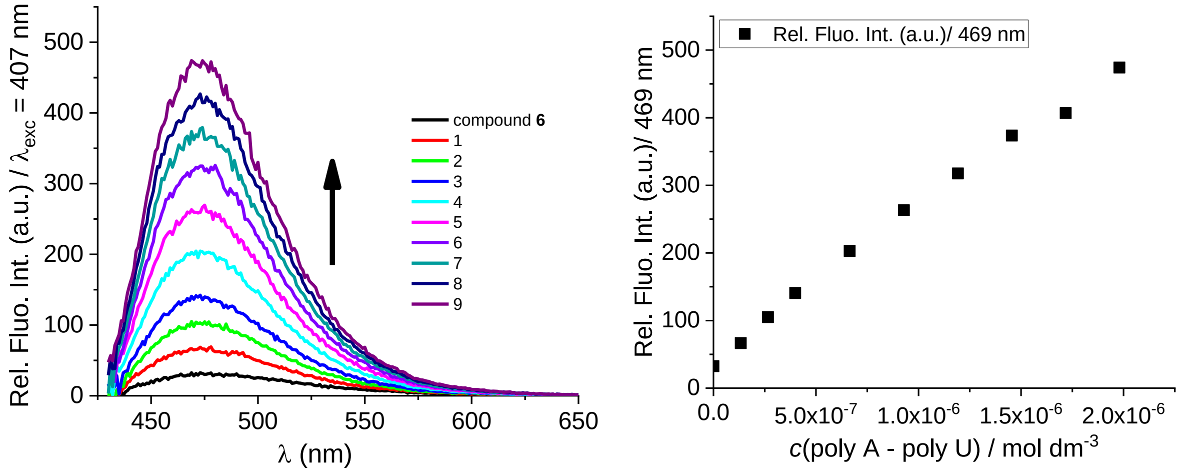

a) b)

Figure S26. a) Changes in fluorescence spectrum of **6** (c= 2.5 × 10^{-7} mol dm^{-3}, λ_{exc}= 407 nm) upon titration with poly A – poly U (c= 1.33 × 10^{-7} – 1.98 × 10^{-6} mol dm^{-3}), S_{exc} = 20, S_{em} = 20; b) Fluorescence intensities of **6** at λ_{max} = 469 nm upon addition of poly A – poly U, at pH=7.0, sodium cacodylate buffer, I = 0.05 mol dm^{-3}.

2.2. Thermal melting experiments

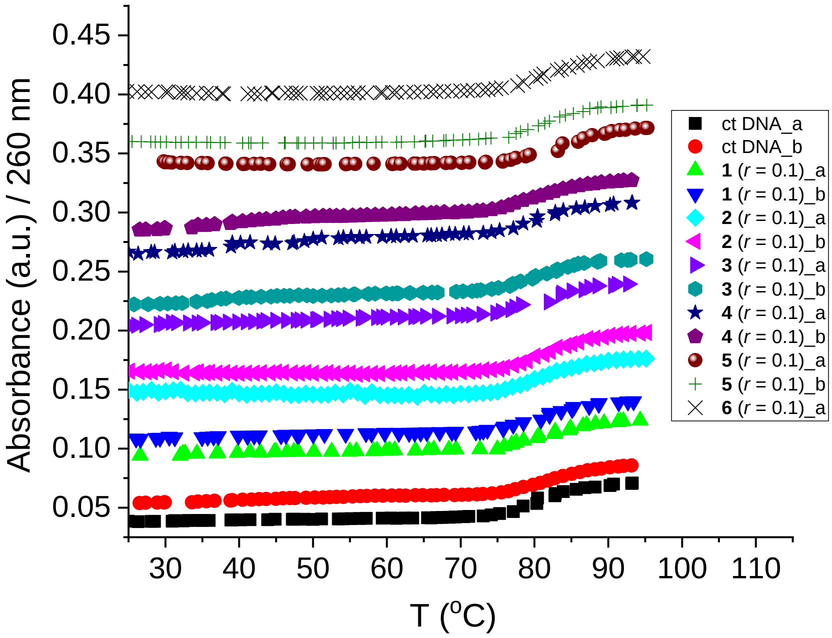

Figure S27. Melting curve of **ct DNA** upon addition of ratio, ***r*** ([compound/ [polynucleotide]) = 0.1 of **1** – **6** at pH = 7.0 (sodium cacodylate buffer, *I* = 0.05 mol dm^{-3}).

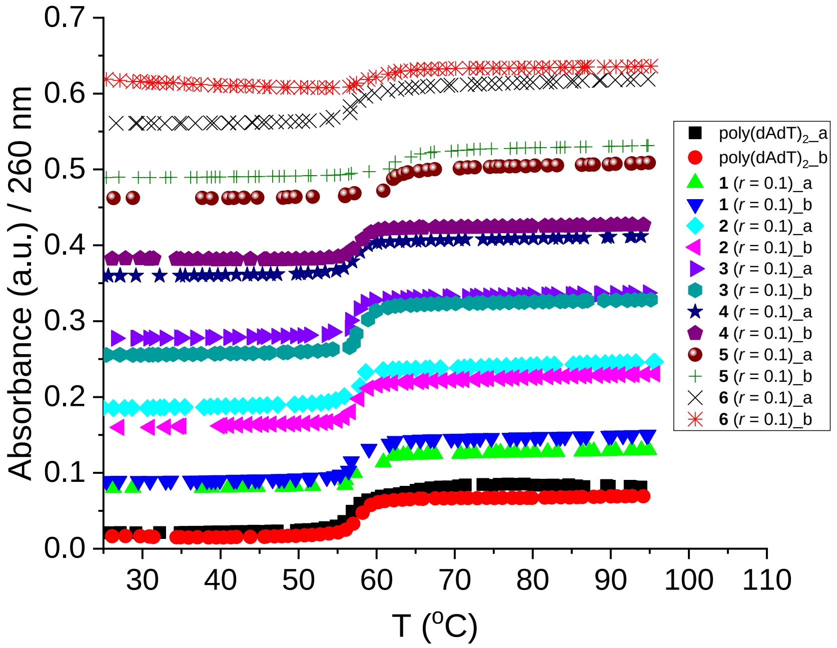

Figure S28. Melting curve of **poly(dAdT)$_2$** upon addition of ratio, ***r*** ([compound/ [polynucleotide]) = 0.1 of **1** – **6** at pH = 7.0 (sodium cacodylate buffer, *I* = 0.05 mol dm^{-3}).

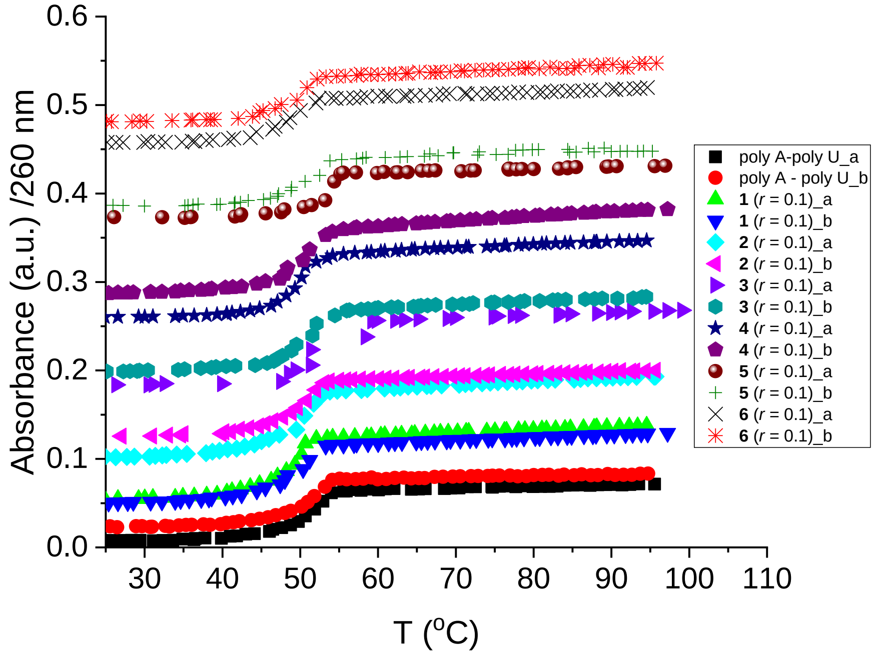

Figure S29. Melting curve of **poly A – poly U** upon addition of ratio, ***r*** ([compound/ [polynucleotide]) = 0.1 of **1 – 6** at pH = 7.0 (sodium cacodylate buffer, *I* = 0.05 mol dm^{-3}).

2.3. Circular dichroism (CD) titrations and corresponding UV Vis spectra

<table>
<tr><th></th><th></th><th>poly(dAdT)$_2$</th><th>poly(dGdC)$_2$</th><th>poly A – poly U</th></tr>
<tr><td>CD</td><td>1</td>
<td>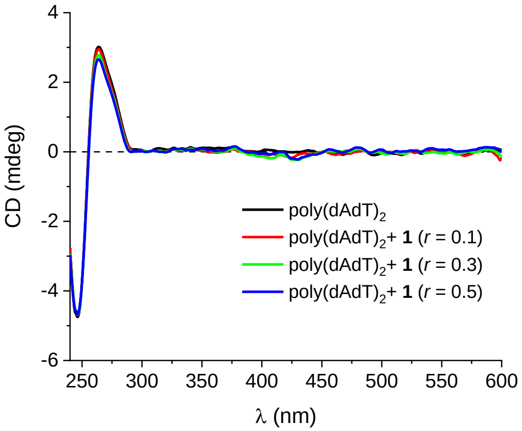
</td>
<td>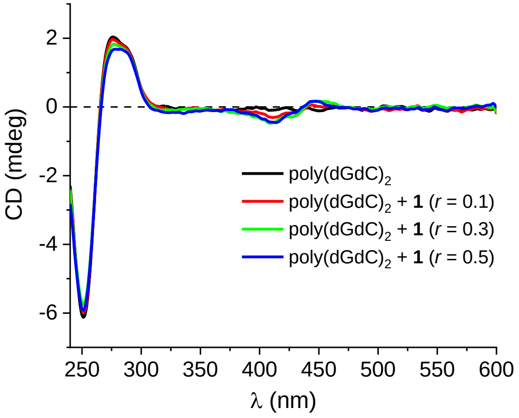
</td>
<td>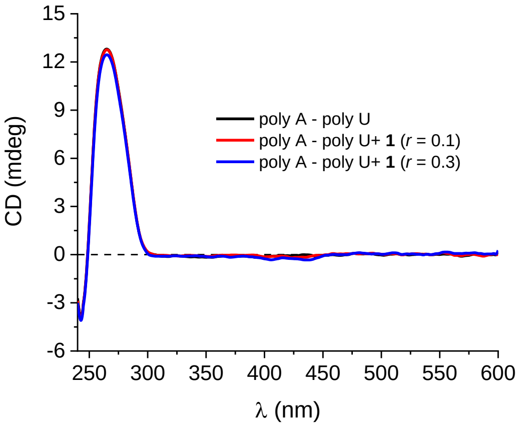
</td></tr>
<tr><td>CD</td><td>2</td>
<td>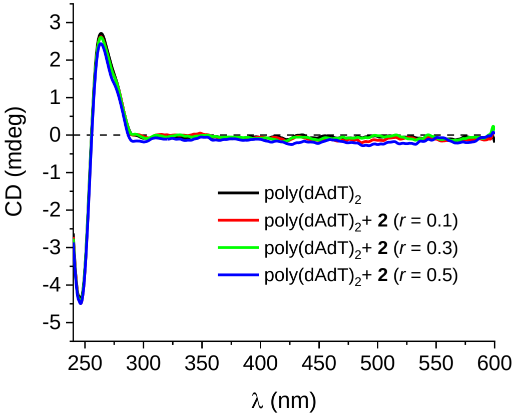
</td>
<td>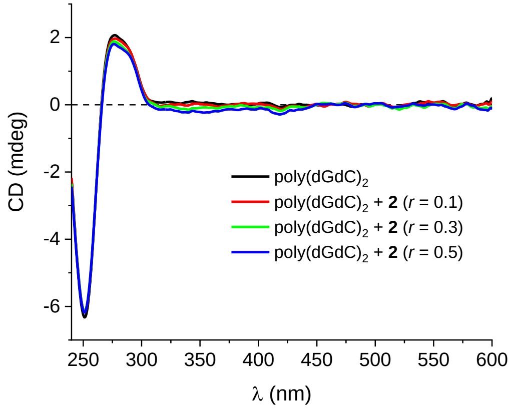
</td>
<td>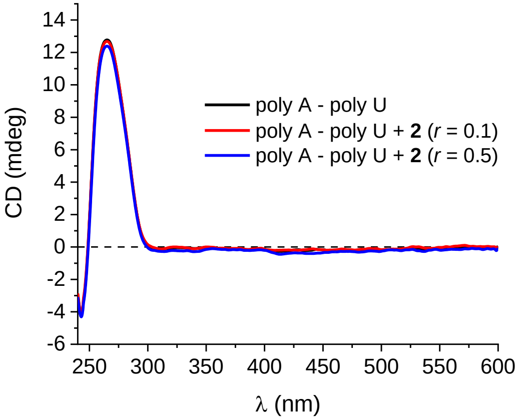
</td></tr>
</table>

a)

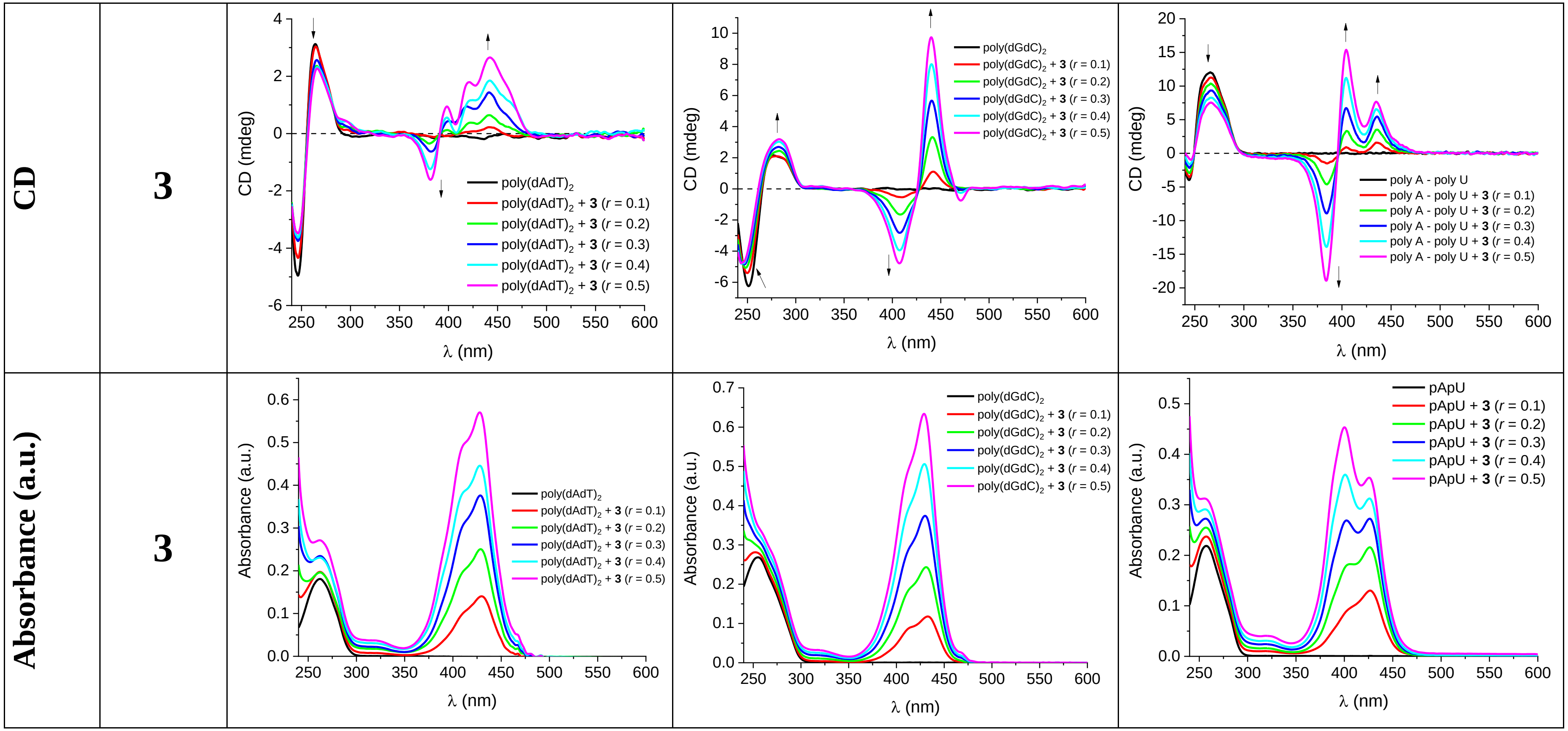
CD
3
Absorbance (a.u.)
3
CD (mdeg)
λ (nm)
Absorbance (a.u.)
poly(dAdT)2
poly(dAdT)2 + 3 (r = 0.1)
poly(dAdT)2 + 3 (r = 0.2)
poly(dAdT)2 + 3 (r = 0.3)
poly(dAdT)2 + 3 (r = 0.4)
poly(dAdT)2 + 3 (r = 0.5)
poly(dGdC)2
poly(dGdC)2 + 3 (r = 0.1)
poly(dGdC)2 + 3 (r = 0.2)
poly(dGdC)2 + 3 (r = 0.3)
poly(dGdC)2 + 3 (r = 0.4)
poly(dGdC)2 + 3 (r = 0.5)
poly A - poly U
poly A - poly U + 3 (r = 0.1)
poly A - poly U + 3 (r = 0.2)
poly A - poly U + 3 (r = 0.3)
poly A - poly U + 3 (r = 0.4)
poly A - poly U + 3 (r = 0.5)
pApU
pApU + 3 (r = 0.1)
pApU + 3 (r = 0.2)
pApU + 3 (r = 0.3)
pApU + 3 (r = 0.4)
pApU + 3 (r = 0.5)

b)

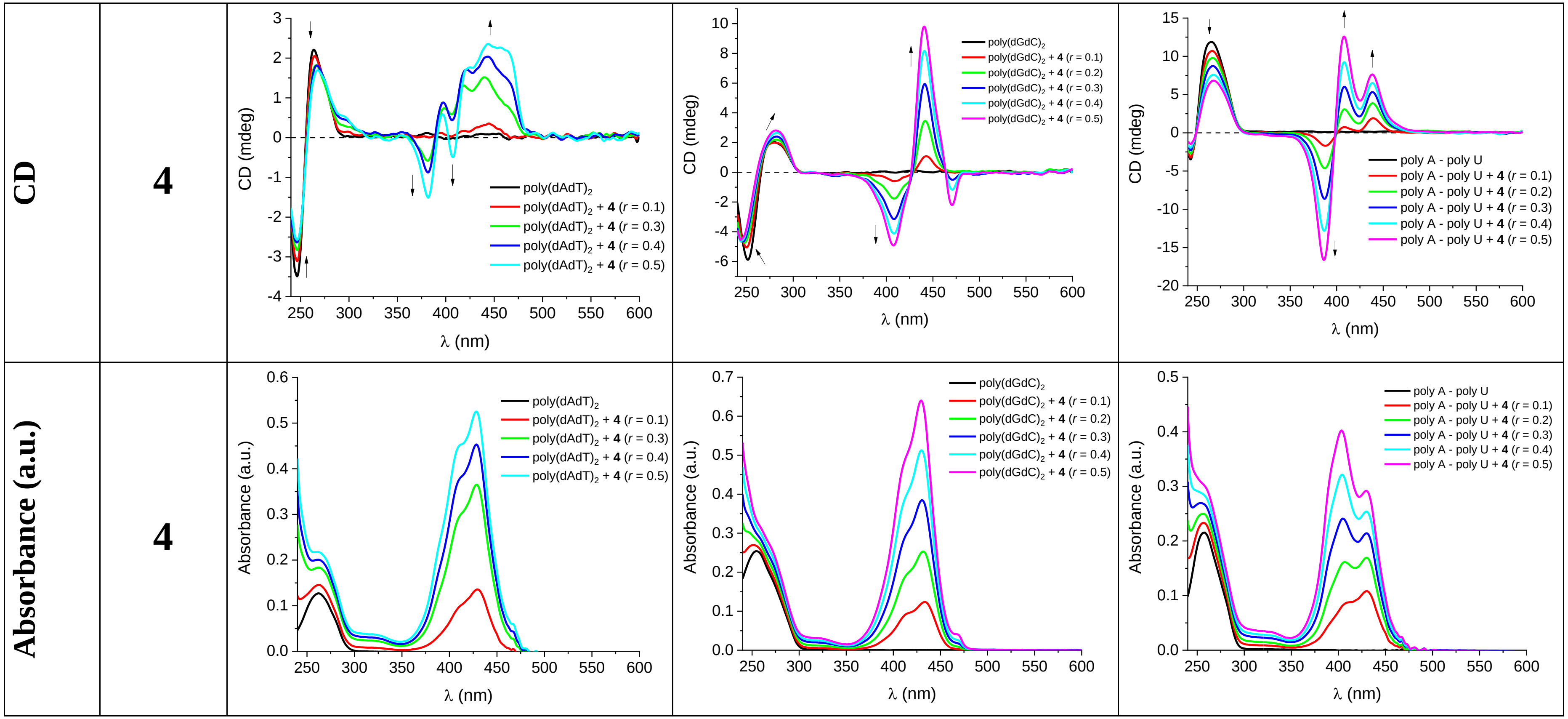
CD
4
CD (mdeg)
λ (nm)
poly(dAdT)$_2$
poly(dAdT)$_2$ + 4 (r = 0.1)
poly(dAdT)$_2$ + 4 (r = 0.3)
poly(dAdT)$_2$ + 4 (r = 0.4)
poly(dAdT)$_2$ + 4 (r = 0.5)
poly(dGdC)$_2$
poly(dGdC)$_2$ + 4 (r = 0.1)
poly(dGdC)$_2$ + 4 (r = 0.2)
poly(dGdC)$_2$ + 4 (r = 0.3)
poly(dGdC)$_2$ + 4 (r = 0.4)
poly(dGdC)$_2$ + 4 (r = 0.5)
poly A - poly U
poly A - poly U + 4 (r = 0.1)
poly A - poly U + 4 (r = 0.2)
poly A - poly U + 4 (r = 0.3)
poly A - poly U + 4 (r = 0.4)
poly A - poly U + 4 (r = 0.5)
Absorbance (a.u.)
4
Absorbance (a.u.)
λ (nm)

c)

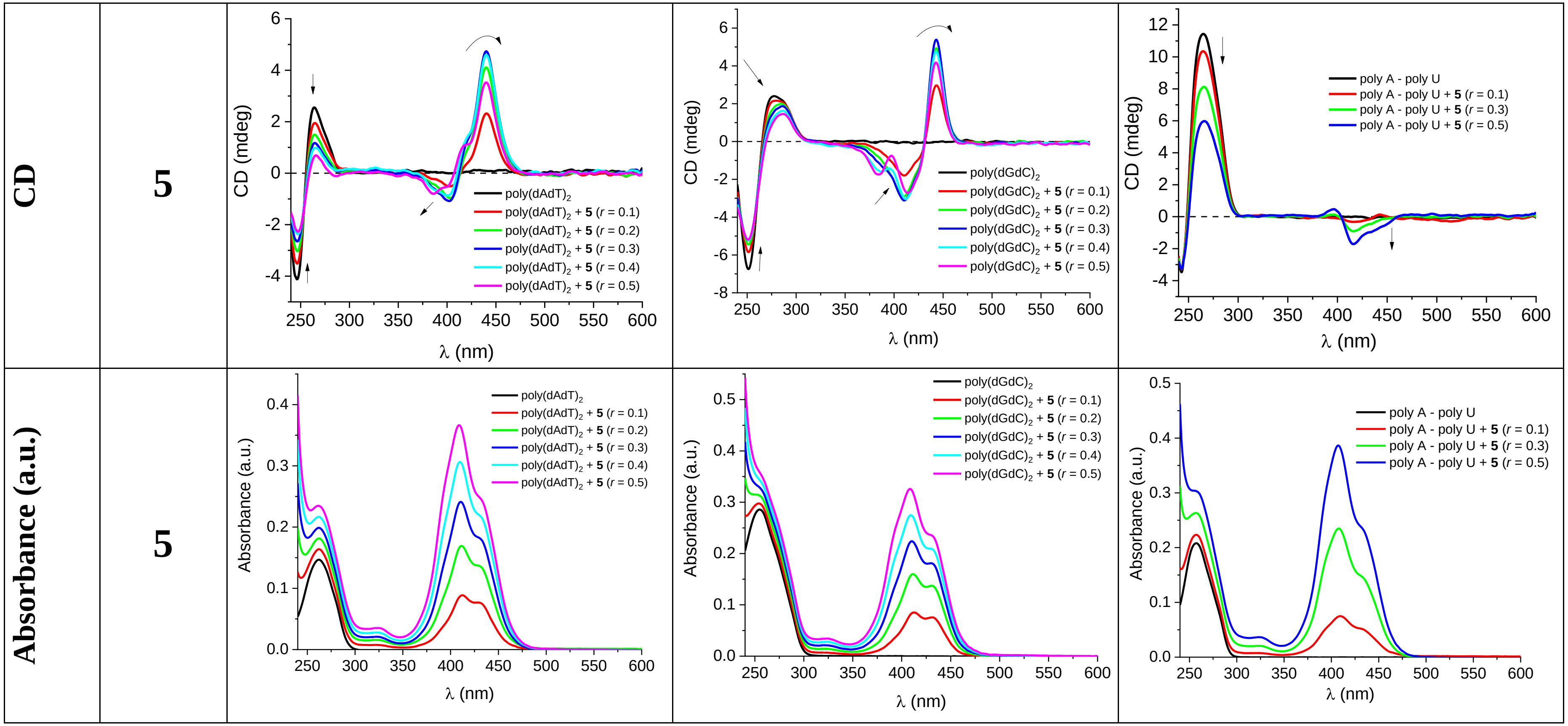
CD
5
Absorbance (a.u.)
5
CD (mdeg)
λ (nm)
poly(dAdT)2
poly(dAdT)2 + 5 (r = 0.1)
poly(dAdT)2 + 5 (r = 0.2)
poly(dAdT)2 + 5 (r = 0.3)
poly(dAdT)2 + 5 (r = 0.4)
poly(dAdT)2 + 5 (r = 0.5)
poly(dGdC)2
poly(dGdC)2 + 5 (r = 0.1)
poly(dGdC)2 + 5 (r = 0.2)
poly(dGdC)2 + 5 (r = 0.3)
poly(dGdC)2 + 5 (r = 0.4)
poly(dGdC)2 + 5 (r = 0.5)
poly A - poly U
poly A - poly U + 5 (r = 0.1)
poly A - poly U + 5 (r = 0.3)
poly A - poly U + 5 (r = 0.5)
Absorbance (a.u.)

d)

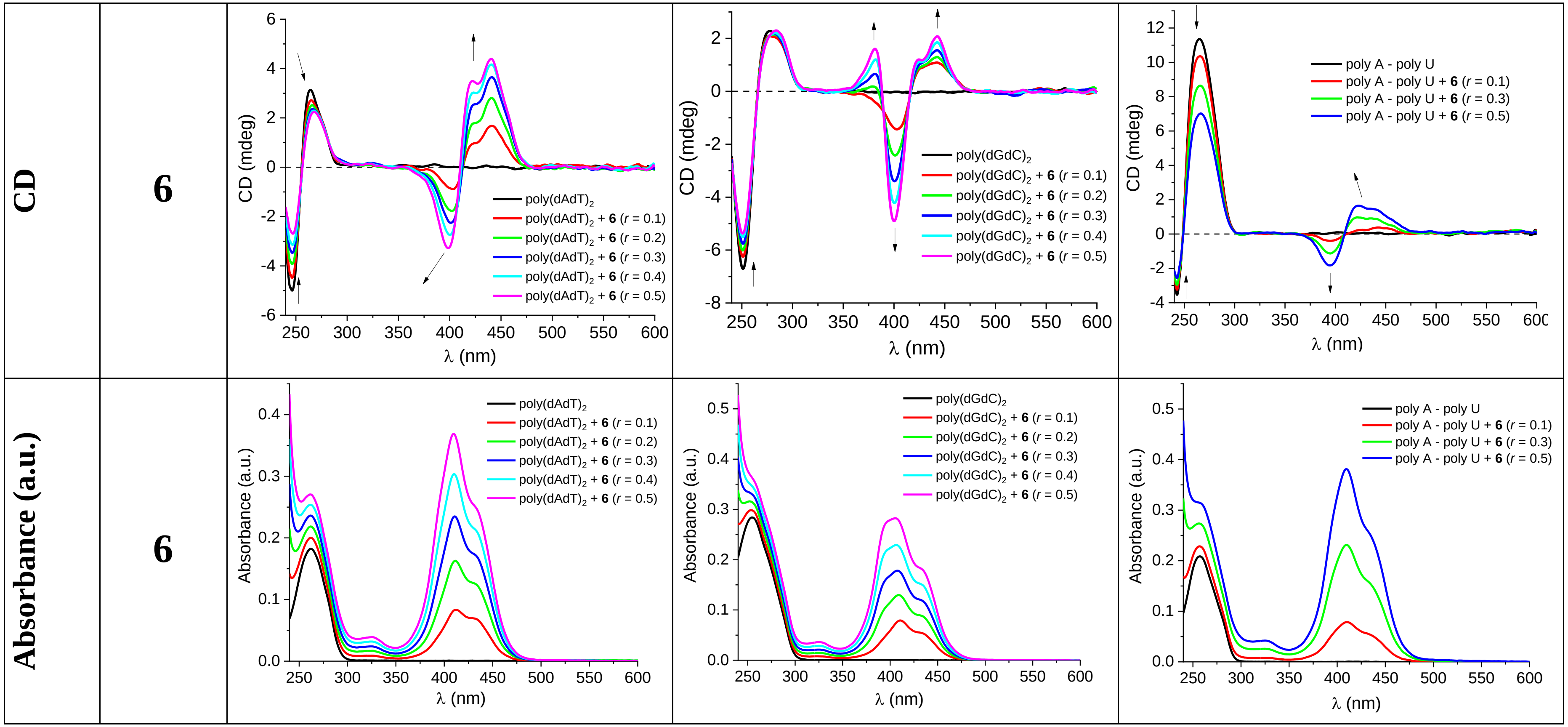

e)

Figure S30. CD titration of poly(dAdT)$_2$, poly(dGdC)$_2$ and poly A-poly U (c = 3.0 × 10^{-5} mol dm^{-3}) with a) **1** and **2**; b) **3** (with corresponding UV spectrum); c) **4** (with corresponding UV spectrum); d) **5** (with corresponding UV spectrum); e) **6** (with corresponding UV spectrum). The experiments were performed in sodium cacodylate buffer, *I* = 0.05 mol dm^{-}3, pH = 7.0, at molar ratios ***r*** = [compound] / [polynucleotide] shown on the graph.

3. Interactions of 1 – 6 compounds with Tel22 in sodium cacodylate buffer (pH=7.0)

3.1. Fluorimetric titrations

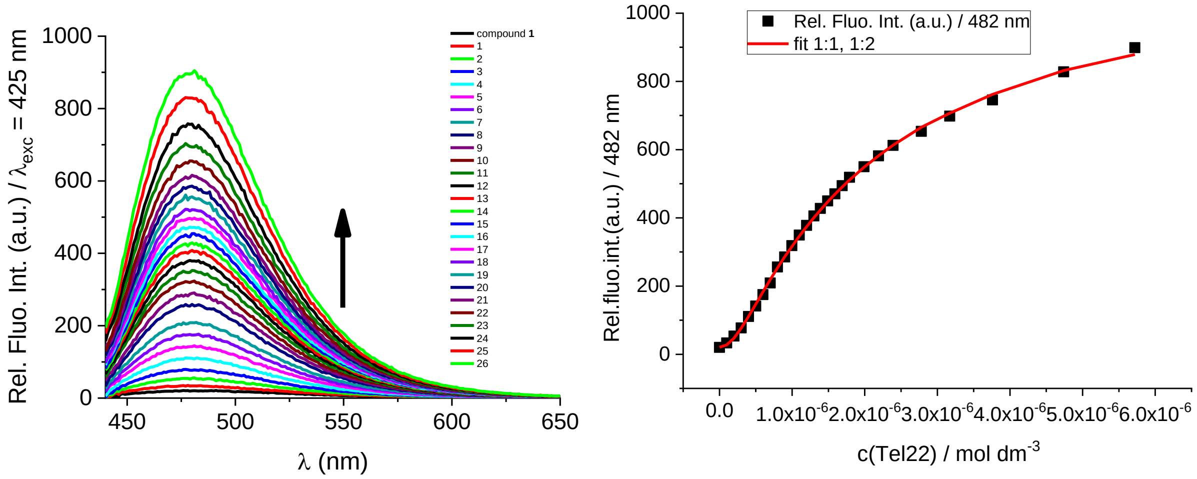

a) b)

Figure S31. a) Changes in fluorescence spectrum of **1** (c= 2.0 × 10^{-7} mol dm^{-3}, λ_{exc}=425 nm) upon titration with Tel22 (c= 1.0 × 10^{-7} – 5.72 × 10^{-6} mol dm^{-3}), S_{exc} = 10, S_{em} = 20; b) Experimental (■) and calculated (—) fluorescence intensities of **1** at λ_{em} = 482 nm upon addition of Tel22, at pH = 7.0, sodium cacodylate buffer, I = 0.1 mol dm^{-3}.

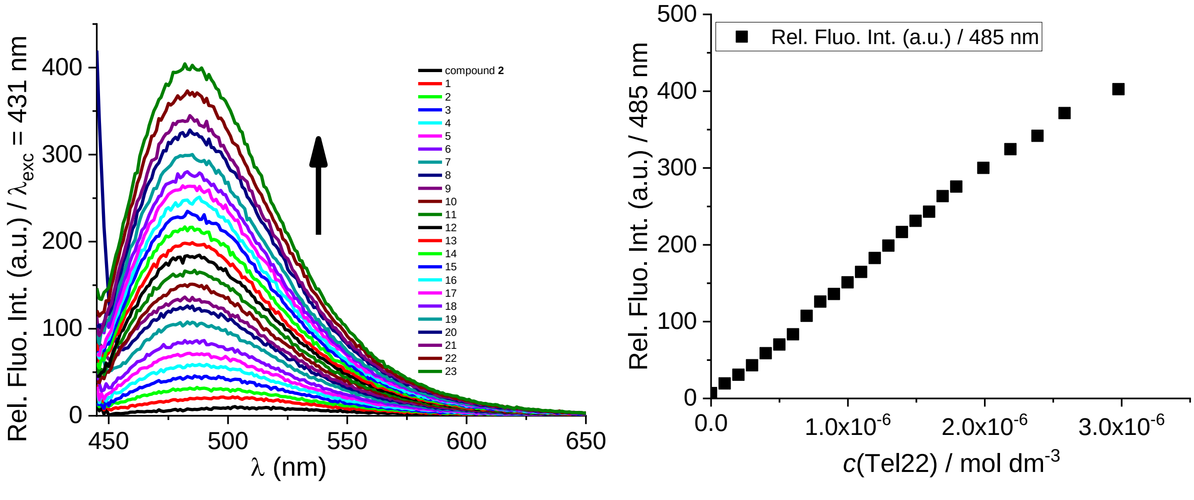

a) b)

Figure S32. a) Changes in fluorescence spectrum of **2** (c= 2.0 × 10^{-7} mol dm^{-3}, λ_{exc}=431 nm) upon titration with Tel22 (c= 1.0 × 10^{-7} – 2.98 × 10^{-6} mol dm^{-3}), S_{exc} = 10, S_{em} = 20; b) Fluorescence intensities of **2** at λ_{max} = 485 nm upon addition of Tel22, at pH=7.0, sodium cacodylate buffer, I = 0.05 mol dm^{-3}.

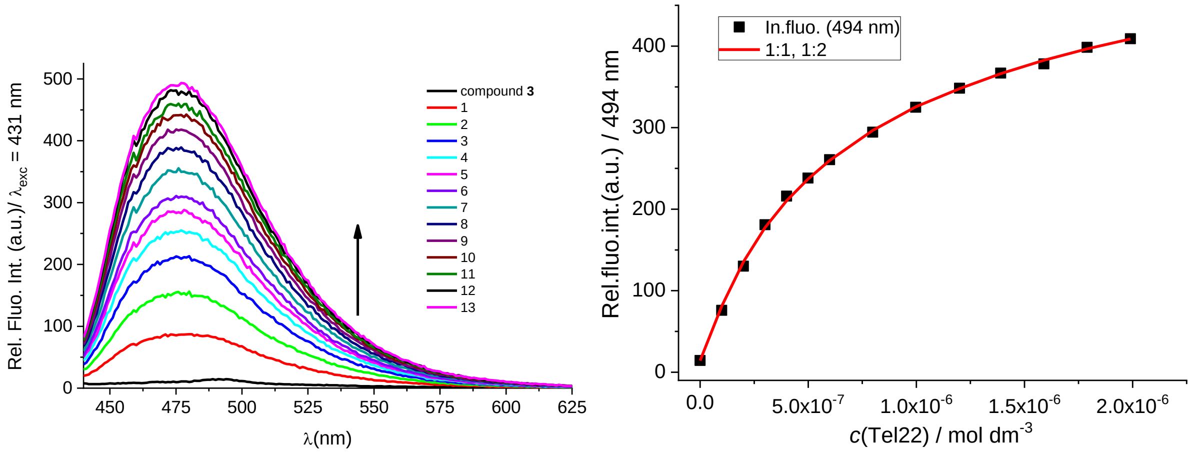

a) b)

Figure S33. Changes in fluorescence spectrum of **3** (c = 2.0 × 10^{-7} mol dm^{-3}, λ_{exc} = 431 nm) upon titration with Tel22 (c = 1.0 × 10^{-7} – 2.98 × 10^{-6} mol dm^{-3}), S_{exc} = 10, S_{em} = 20; b) Experimental (■) and calculated (—) fluorescence intensities of **3** at λ_{em} = 494 nm upon addition of Tel22, at pH = 7.0, sodium cacodylate buffer, I = 0.1 mol dm^{-3}.

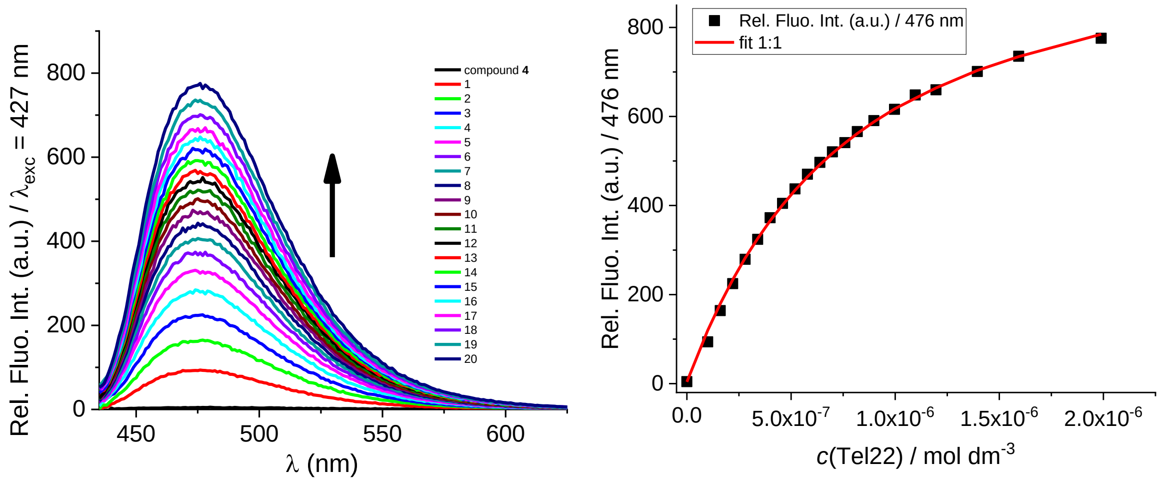

a) b)

Figure S34. a) Changes in fluorescence spectrum of **4** (c= 2.0 × 10^{-7} mol dm^{-3}, λ_{exc}=427 nm) upon titration with Tel22 (c= 1.0 × 10^{-7} – 1.99 × 10^{-6} mol dm^{-3}), S_{exc} = 10, S_{em} = 10; b) Experimental (■) and calculated (—) fluorescence intensities of **4** at λ_{max} = 476 nm upon addition of Tel22, at pH=7, sodium cacodylate buffer, I = 0.05 mol dm^{-3}.

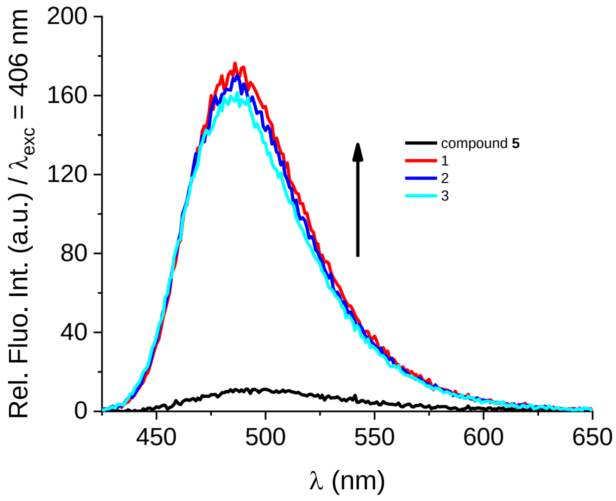

a)

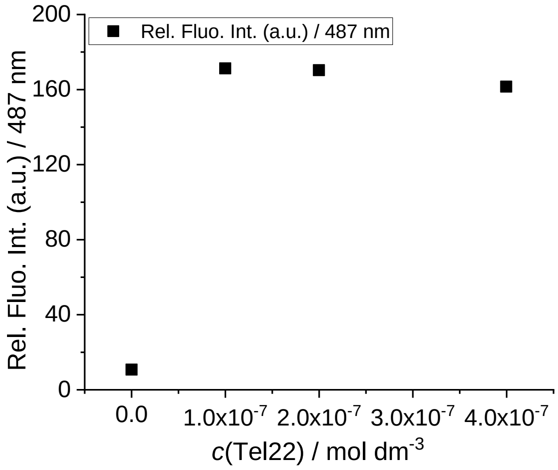

b)

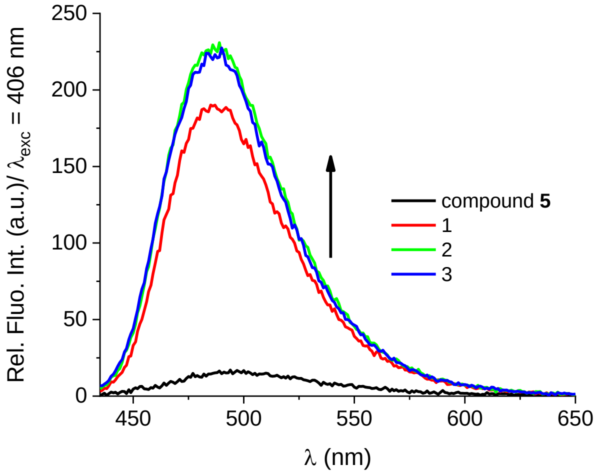

c)

Figure S35. a) Changes in fluorescence spectrum of **5** (c= 2.0 × 10^{-7} mol dm^{-3}, λ_{exc}=406 nm) upon titration with Tel22 (c= 1.0 × 10^{-7} – 4.0 × 10^{-7} mol dm^{-3}), S_{exc} = 10, S_{em} = 10; b) Fluorescence intensities of **5** at λ_{max} = 487 nm upon addition of Tel22, at pH=7.0, sodium cacodylate buffer, I = 0.05 mol dm^{-3}; c) Changes in fluorescence spectrum of **5** (c= 4.0 × 10^{-7} mol dm^{-3}, λ_{exc}=406 nm) upon titration with Tel22 (c= 2.0 × 10^{-8} – 6.0 × 10^{-8} mol dm^{-3}), S_{exc} = 10, S_{em} = 10, sodium cacodylate buffer, I = 0.05 mol dm^{-3}, at pH=7.0.

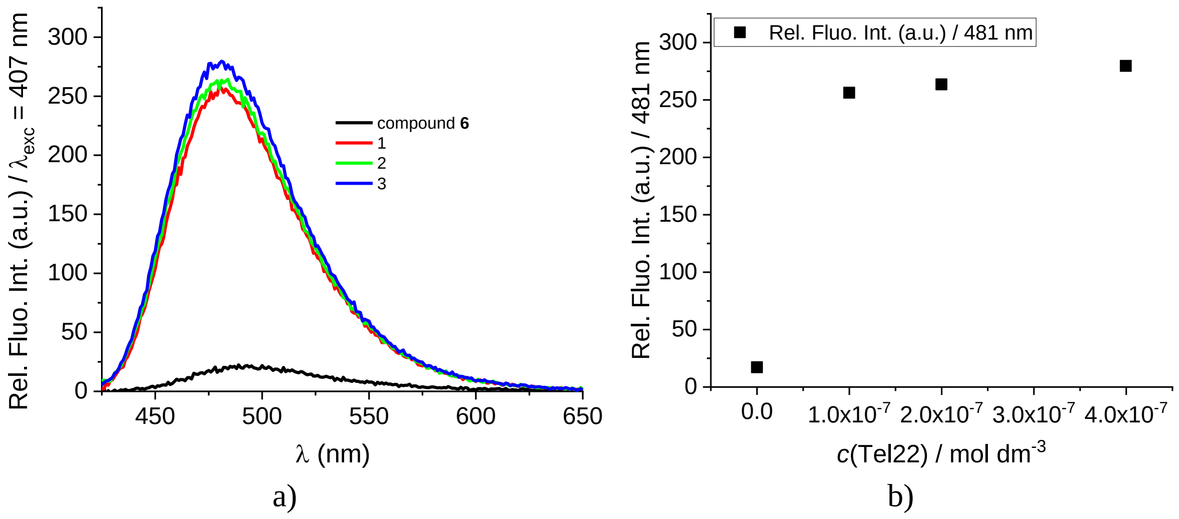

Figure S36. a) Changes in fluorescence spectrum of **6** (c= 2.0 × 10^{-7} mol dm^{-3}, λ_{exc}=407 nm) upon titration with Tel22 (c= 1.0 × 10^{-7} – 4.0 × 10^{-7} mol dm^{-3}), S_{exc} = 10, S_{em} = 20; b) Fluorescence intensities of **5** at λ_{max} = 481 nm upon addition of Tel22, at pH=7.0, sodium cacodylate buffer, I = 0.05 mol dm^{-3}.

.

3.2. Thermal melting experiments

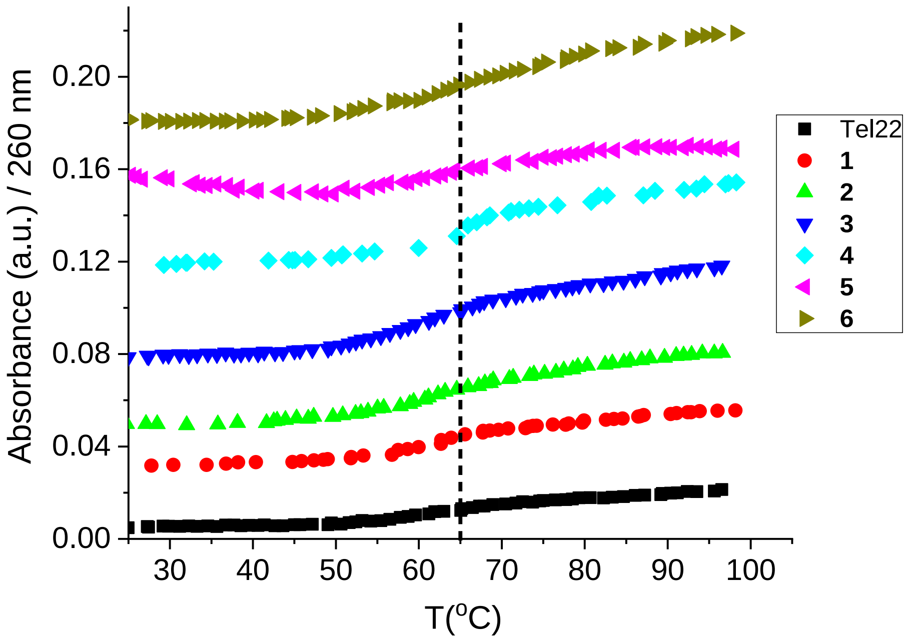

Figure S37. Melting curves of Tel22 upon addition of compounds 1-6; c (Tel22) =2.0 ×10^{-6} M; ratio, ***r*** =[compound] / [oligonucleotide] = 1 (pH = 7.0, sodium cacodylate buffer, I = 0.1 mol dm^{-3}).

3.3. Circular dichroism (CD) titrations

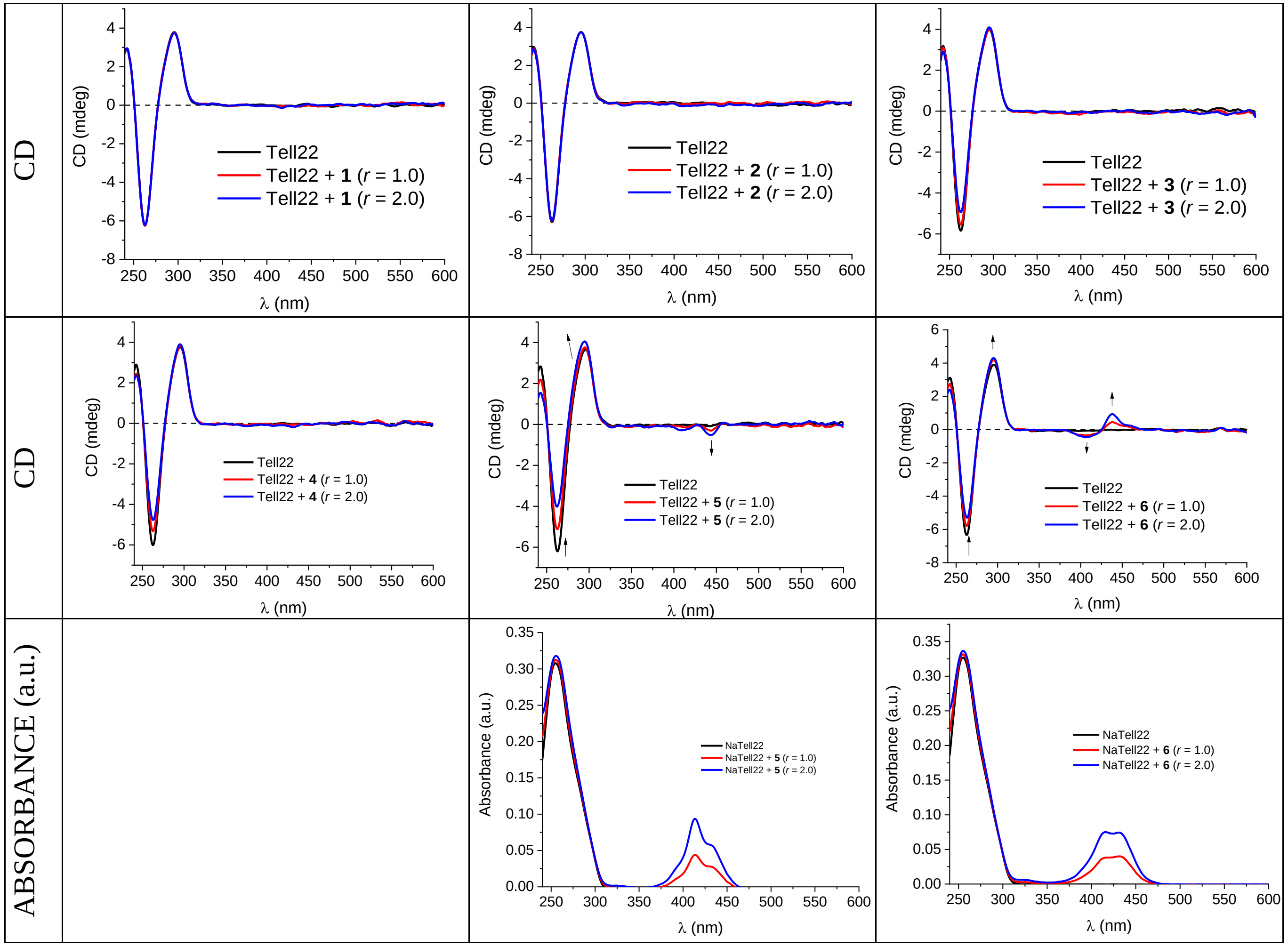

Figure S38. CD titration of Tel22 (c = 1.5 × 10^{-6} mol dm^{-3}) with **1** – **6** at molar ratios ***r*** = [compound] / [polynucleotide] indicated in the graph legend (pH = 7.0, sodium cacodylate buffer, I = 0.1 mol dm^{-3}) and corresponding absorption spectra of Tel22 with **5** and **6** at the same molar ratios**.**

4. Interactions of 1 and 3 to 6 with Tel22 in potassium phosphate buffer (pH=7.0)

4.1. Fluorimetric titrations

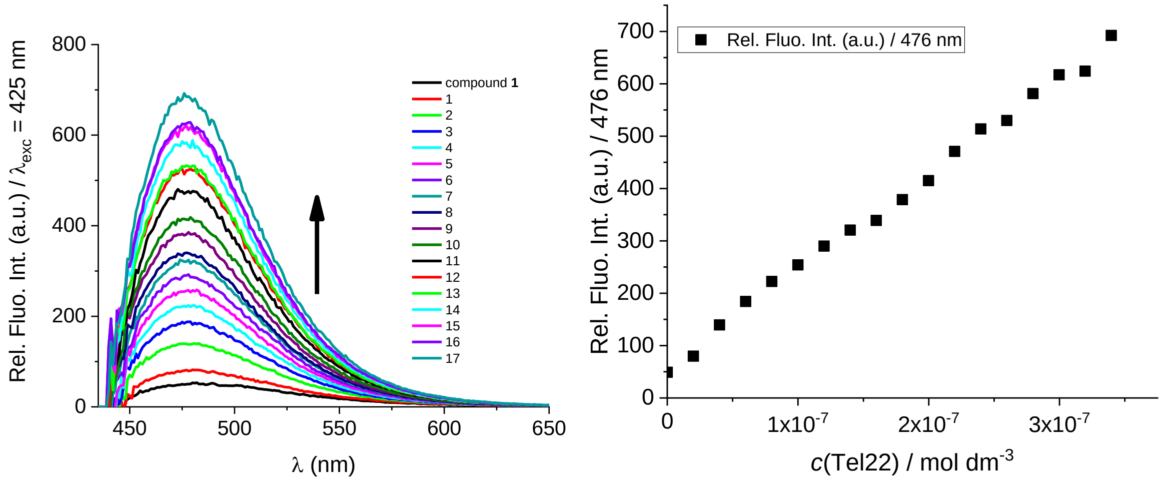

a) b)

Figure S39. a) Changes in fluorescence spectrum of **1** (c= 2.0 × 10^{-7} mol dm^{-3}, λ_{exc}=425 nm) upon titration with Tel22 (c= 2.0 × 10^{-8} – 3.40 × 10^{-7} mol dm^{-3}), S_{exc} = 20, S_{em} = 20; b) Fluorescence intensities of **1** at λ_{max} = 476 nm upon addition of Tel22, at pH=7.0, potassium phosphate buffer, I = 0.05 mol dm^{-3}.

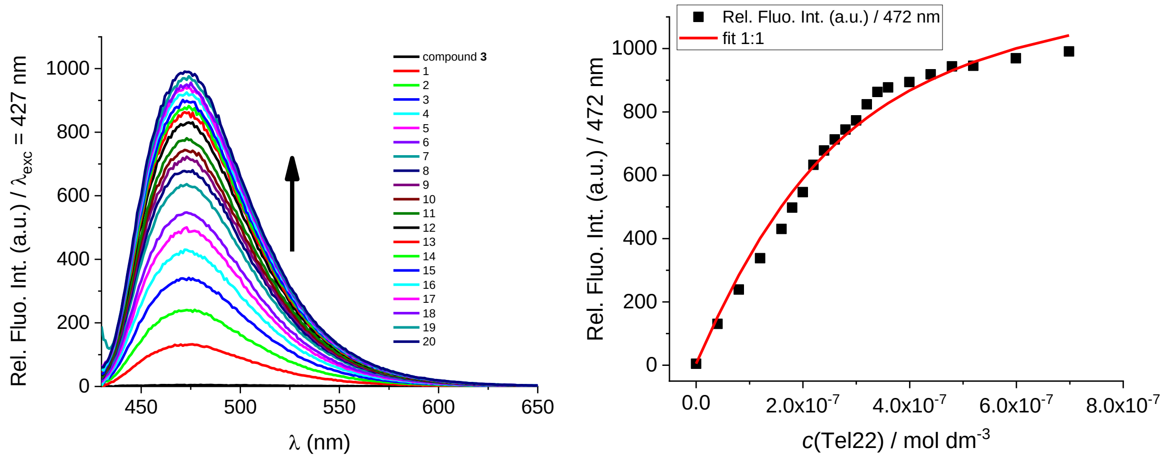

a) b)

Figure S40. a) Changes in fluorescence spectrum of **3** (c= 2.0 × 10^{-7} mol dm^{-3}, λ_{exc}=431 nm) upon titration with Tel22 (c= 4.0 × 10^{-8} – 6.99 × 10^{-7} mol dm^{-3}), S_{exc} = 10, S_{em} = 10; b) Experimental (■) and calculated (—) fluorescence intensities of **3** at λ_{max} = 472 nm upon addition of Tel22, at pH=7.0, potassium phosphate buffer, I = 0.05 mol dm^{-3}.

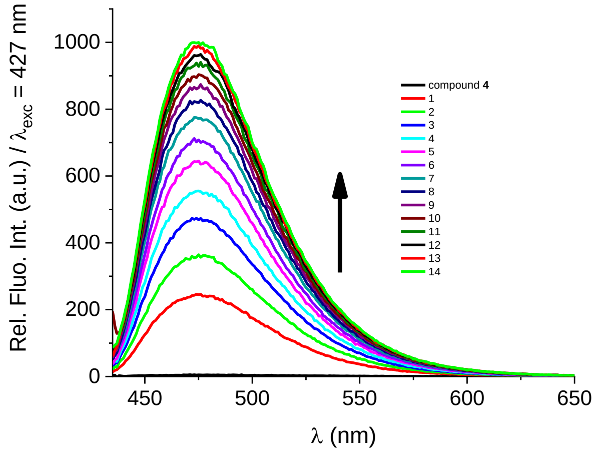

a)

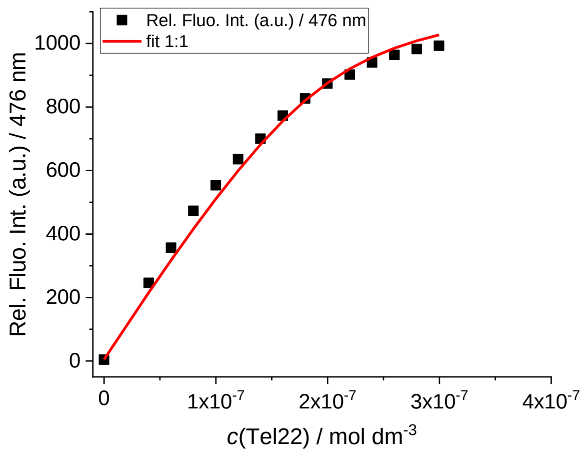

b)

Figure S41. a) Changes in fluorescence spectrum of **4** (c = 2.0 × 10^{-7} mol dm^{-3}, λ_{exc}=427 nm) upon titration with Tel22 (c = 4.0 × 10^{-8} – 3.0 × 10^{-7} mol dm^{-3}), S_{exc} = 10, S_{em} = 10; b) Experimental (■) and calculated (—) fluorescence intensities of **4** at λ_{max} = 476 nm upon addition of Tel22, at pH=7.0, potassium phosphate buffer, I = 0.05 mol dm^{-3}.

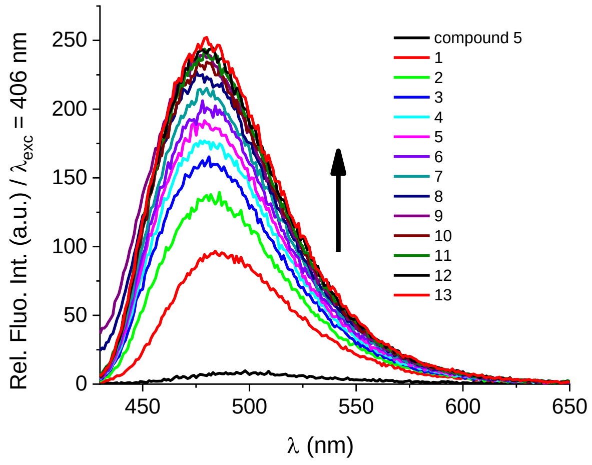

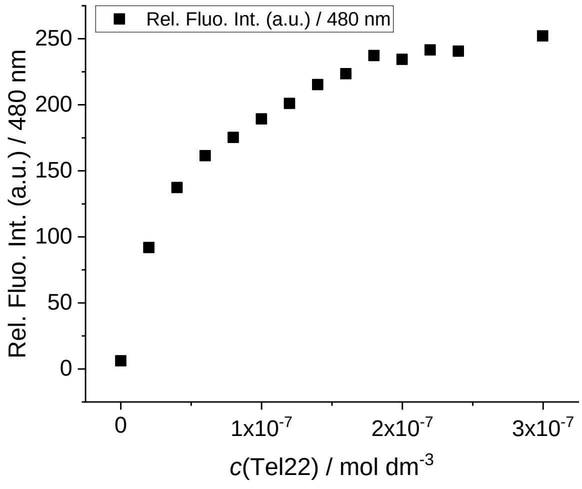

a)

b)

Figure S42. a) Changes in fluorescence spectrum of **5** (c = 2.0 × 10^{-7} mol dm^{-3}, λ_{exc} = 406 nm) upon titration with Tel22 (c= 2.0 × 10^{-8} – 3.0 × 10^{-7} mol dm^{-3}), S_{exc} = 10, S_{em} = 10; b) Fluorescence intensities of **5** at λ_{max} = 480 nm upon addition of Tel22, at pH=7.0, potassium phosphate buffer, I = 0.05 mol dm^{-3}.

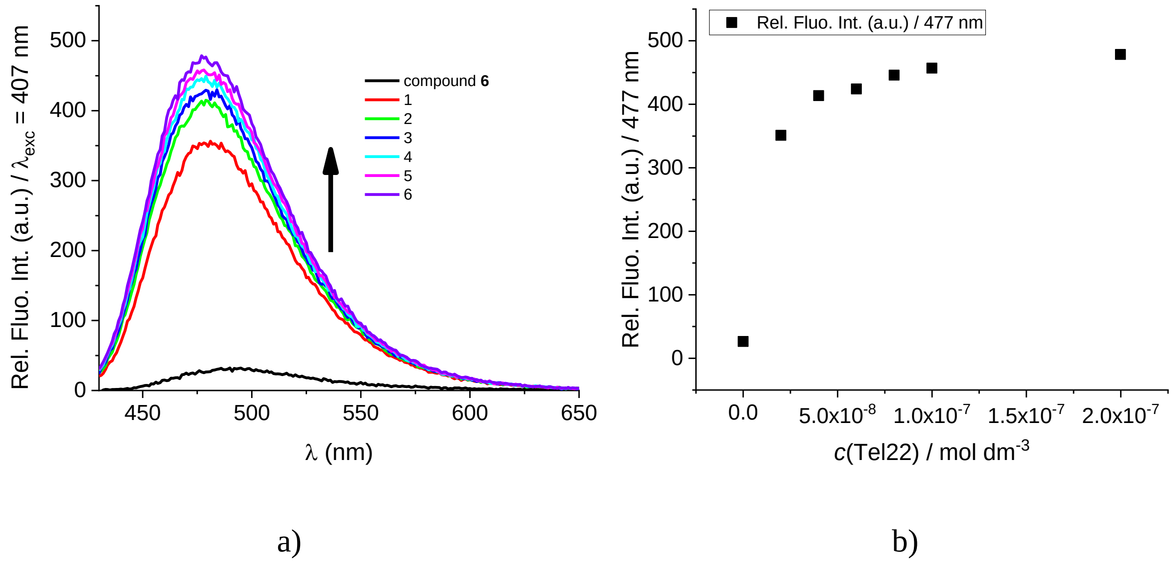

Figure S43. a) Changes in fluorescence spectrum of **6** (c = 2.0 × 10^{-7} mol dm^{-3}, λ_{exc}= 407 nm) upon titration with Tel22 (c = 2.0 × 10^{-8} – 2.0 × 10^{-7} mol dm^{-3}), S_{exc} = 10, S_{em} = 20; b) Fluorescence intensities of **6** at λ_{max} = 477 nm upon addition of Tel22, at pH=7.0, potassium phosphate buffer, I = 0.05 mol dm^{-3}.

4.2. Circular dichroism (CD) titrations

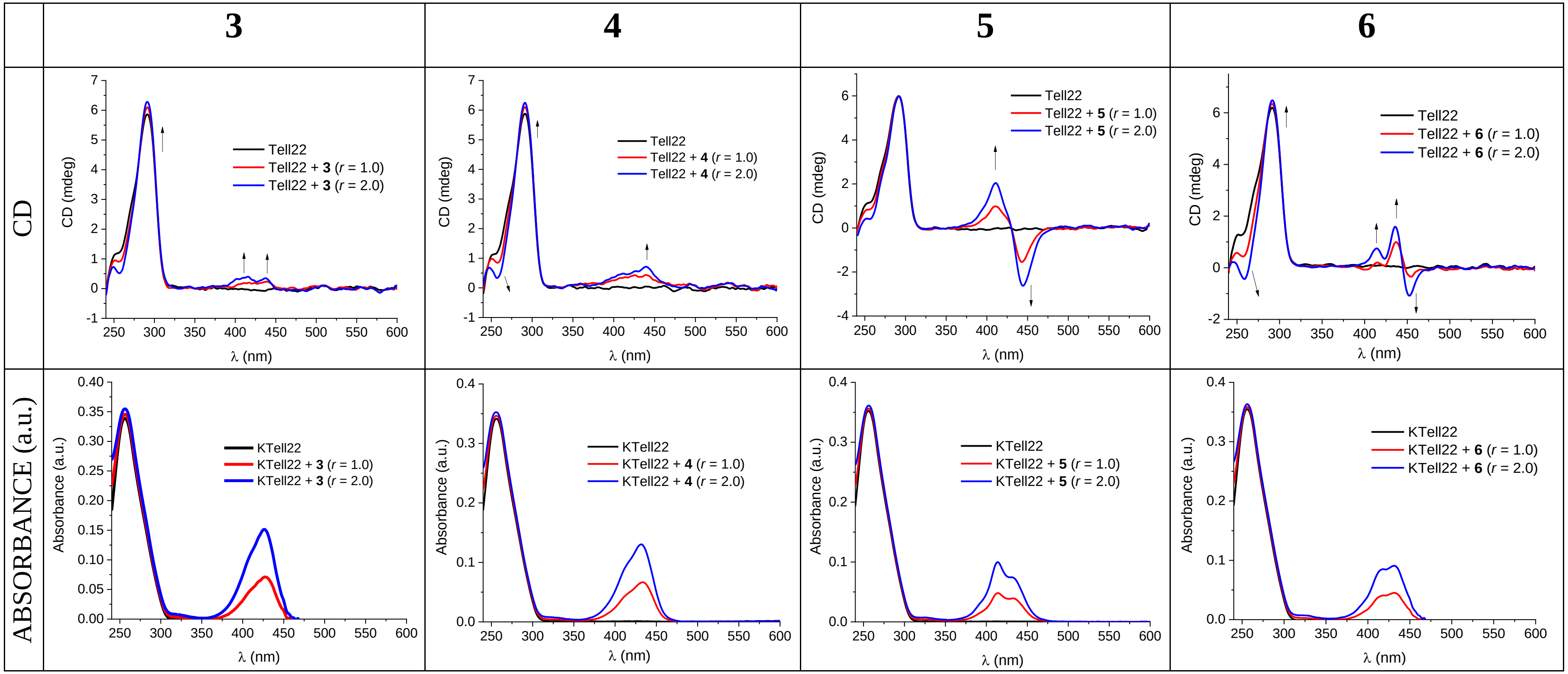

Figure S44. CD titration of Tel22 ($c = 1.5 \times 10^{-6}$ mol dm^{-3}) with **3** – **6** at molar ratios ***r*** = [compound] / [polynucleotide] indicated in the graph legend (pH = 7.0, potassium phosphate buffer, $I = 0.1$ mol dm^{-3}) and corresponding absorption spectra at the same molar ratios.

4.3. Thermal melting experiments

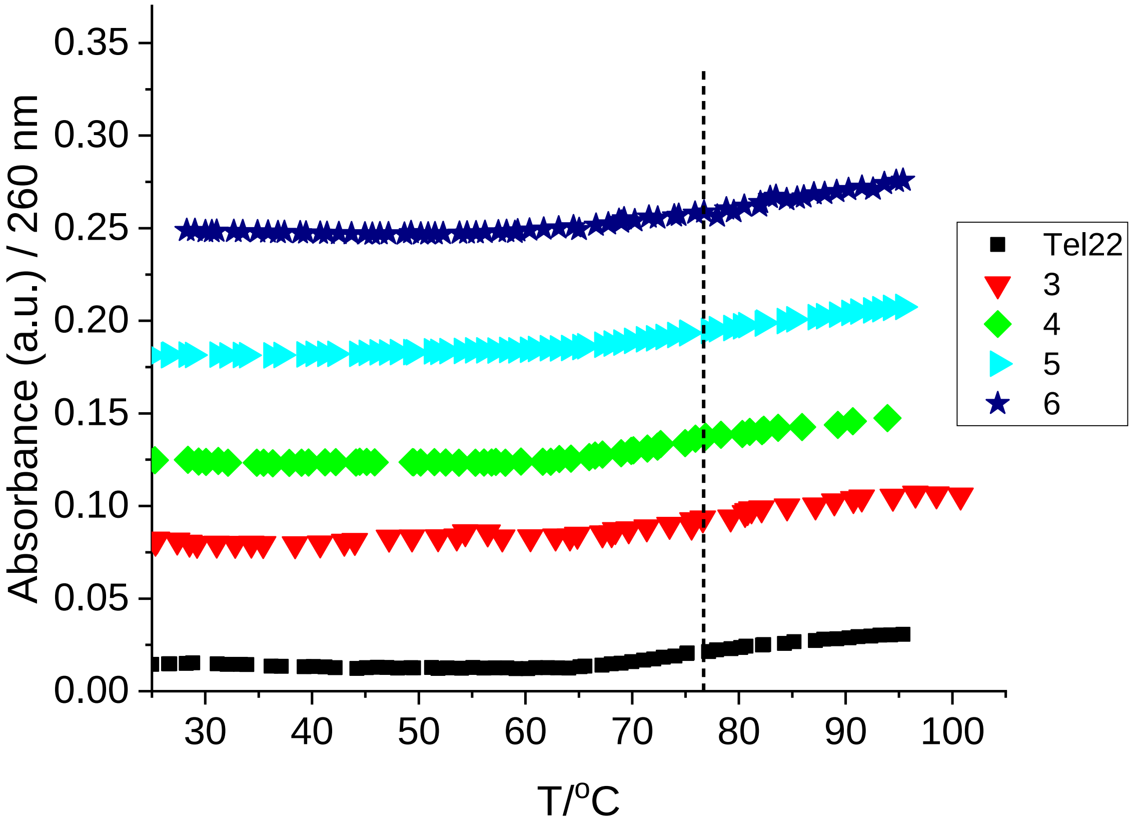

Figure S45. Melting curves of Tel22 upon addition of compounds **3-6**; c (Tel22) =2.0 ×10^{-6} M; ratio, ***r*** =[compound] / [oligonucleotide] = 1 (pH = 7.0, potassium phosphate buffer, *I* = 0.1 mol dm^{-3}).

5. Confocal microscopy

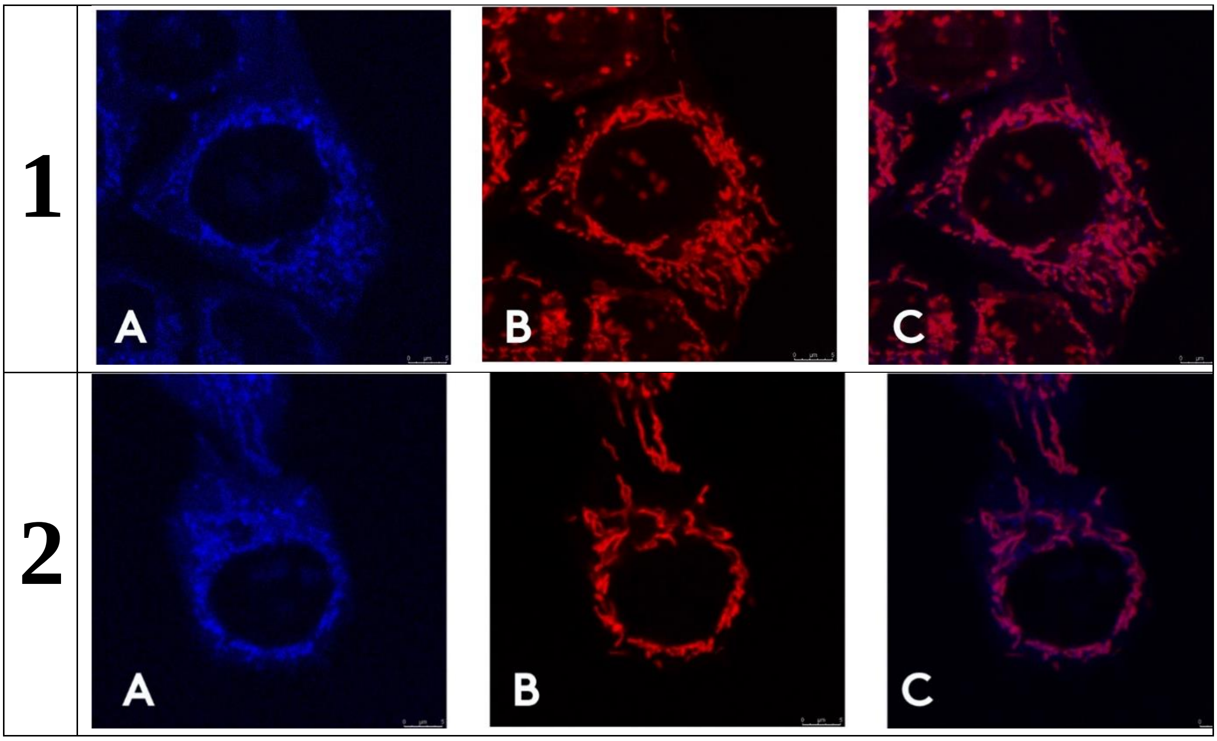

Figure S46. Intracellular distribution of dye **1** and **2** compared to MitoTracker. Confocal microscopy of live HeLa cells taken on Leica SP8 X confocal microscope, stained with 1 μM of compounds **1** or **2**; A) channel showing emission of **1** and **2** (λ_{exc} = 510 nm, λ_{em} = 470-670 nm); B) MitoTracker channel (λ_{exc} = 644 nm, λ_{em} = 665-700nm); C) overlay of the two channels.

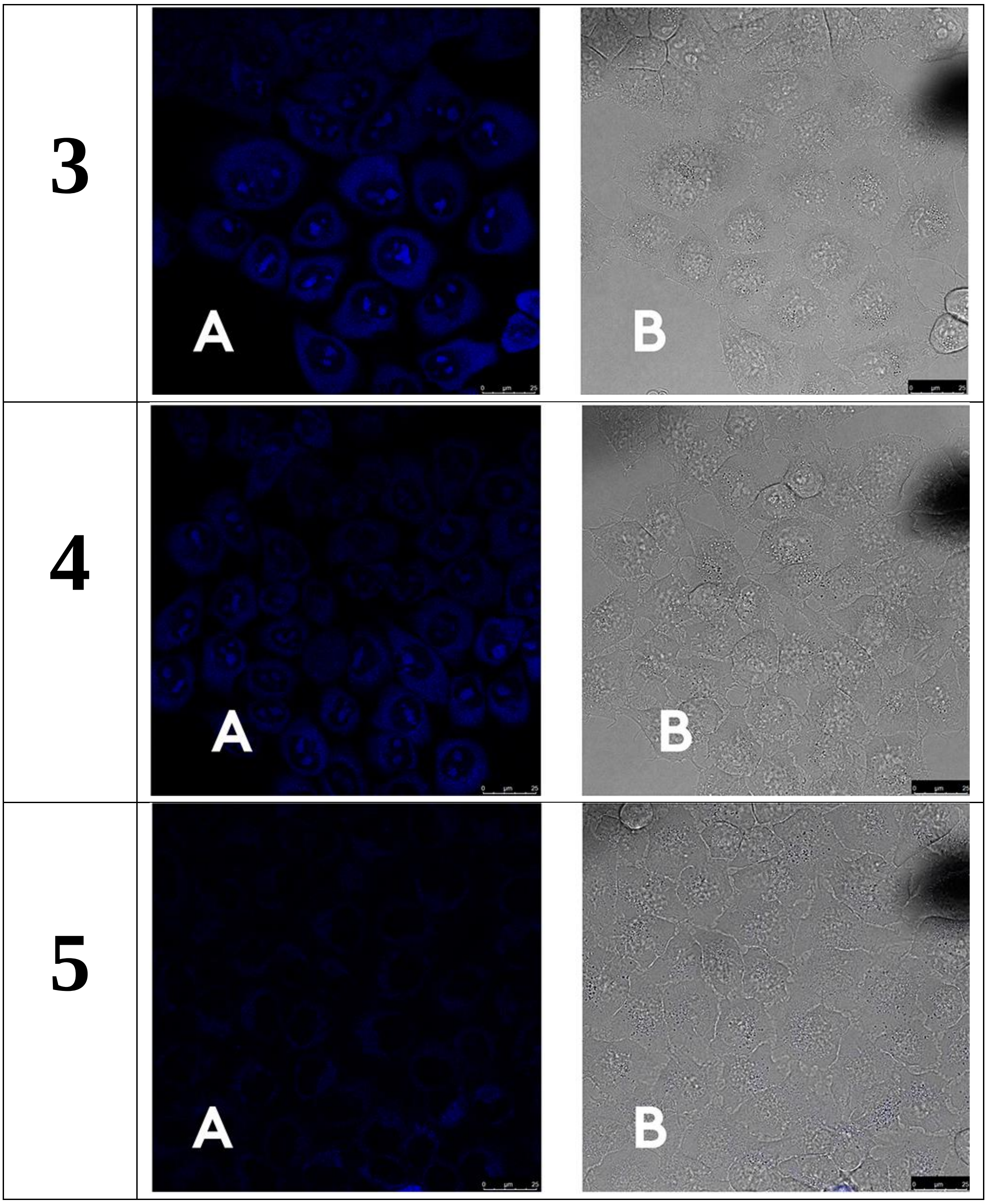

Figure S47. Intracellular distribution of dye **3, 4** and **5**. Confocal microscopy of live HeLa cells taken on Leica SP8 X confocal microscope, stained with 1 μM of compounds **3, 4** or **5** (λ_{exc} = 405 nm, λ_{em} = 485-510 nm).

6. HRMS and NMR spectra

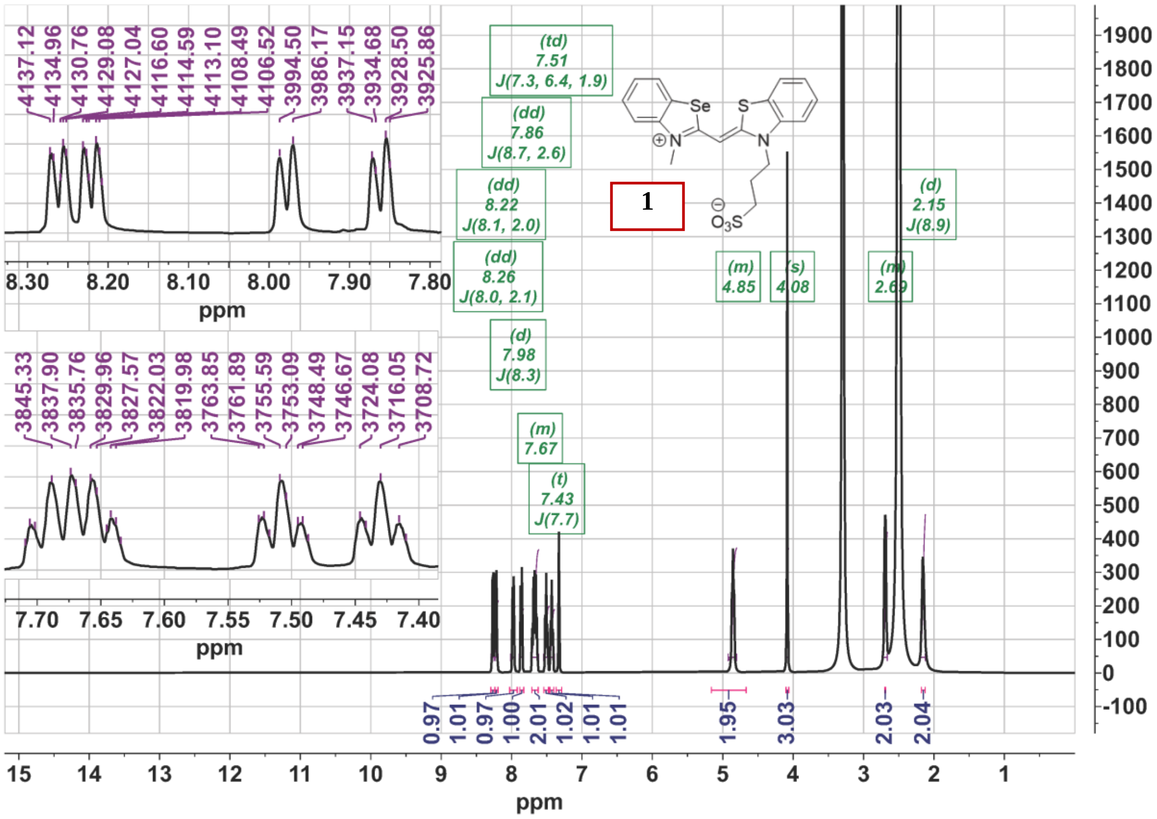

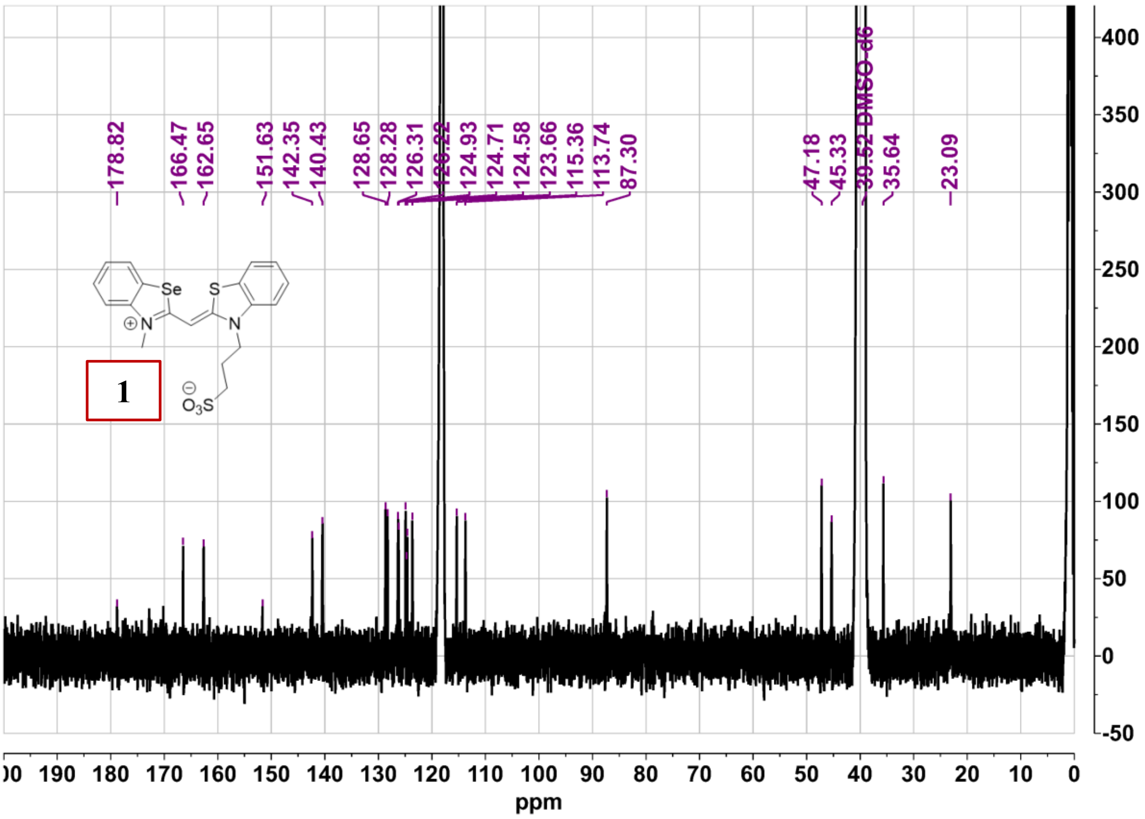

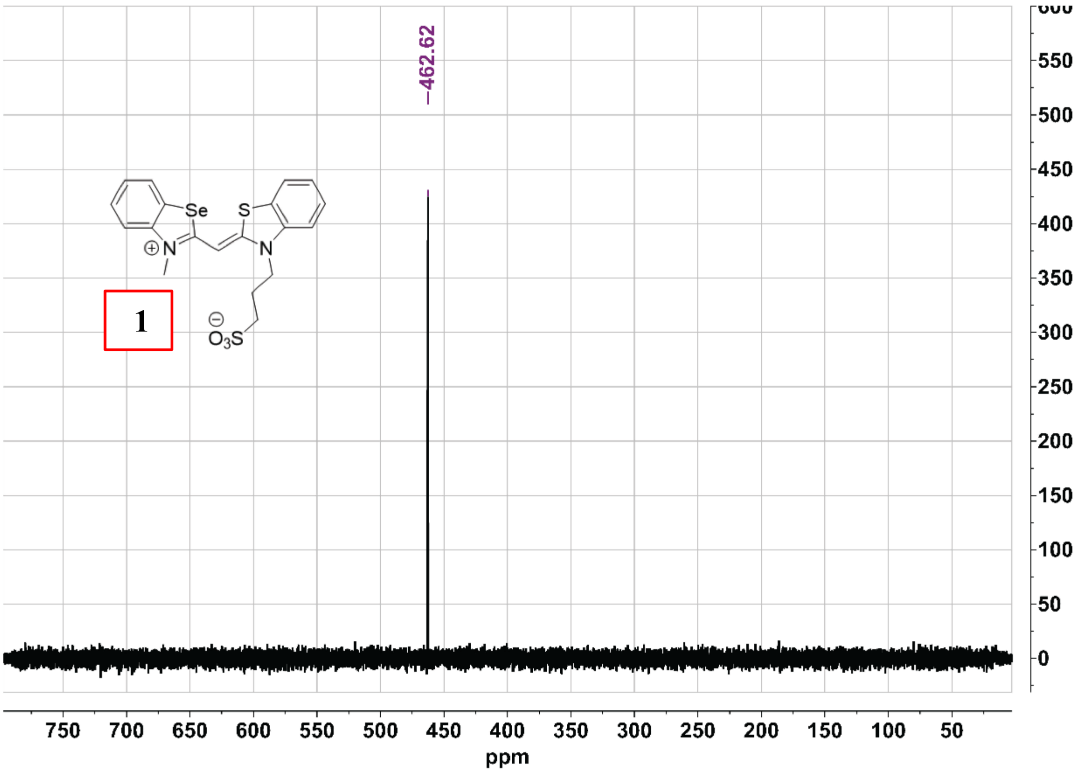

−462.62
Se
S
N
N
1
O3S
750 700 650 600 550 500 450 400 350 300 250 200 150 100 50
ppm
600 550 500 450 400 350 300 250 200 150 100 50 0

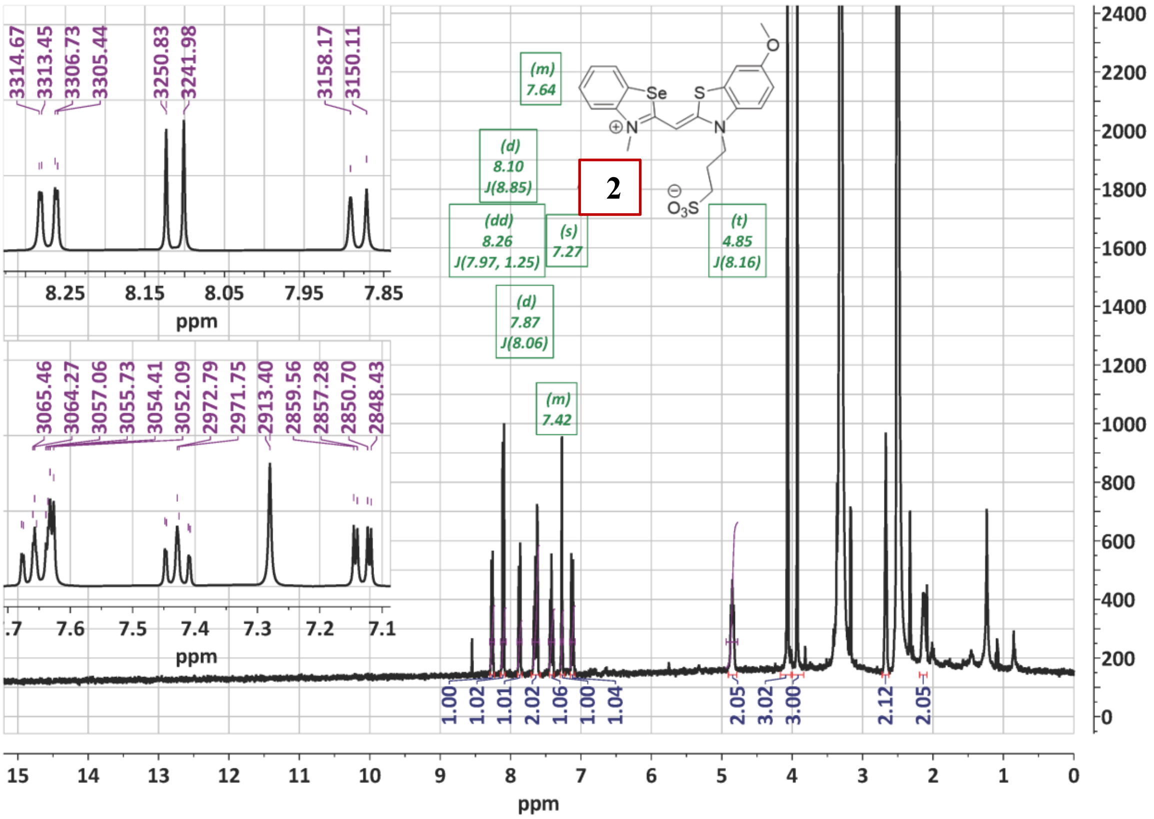

3314.67
3313.45
3306.73
3305.44
3250.83
3241.98
3158.17
3150.11
8.25 8.15 8.05 7.95 7.85
ppm
3065.46
3064.27
3057.06
3055.73
3054.41
3052.09
2972.79
2971.75
2913.40
2859.56
2857.28
2850.70
2848.43
.7 7.6 7.5 7.4 7.3 7.2 7.1
ppm
(m) 7.64
(d) 8.10 J(8.85)
(dd) 8.26 J(7.97, 1.25)
(s) 7.27
(d) 7.87 J(8.06)
(m) 7.42
(t) 4.85 J(8.16)
2
Se
S
N
N
O
O3S
1.00 1.02 1.01 2.02 1.06 1.00 1.04 2.05 3.02 3.00 2.12 2.05
15 14 13 12 11 10 9 8 7 6 5 4 3 2 1 0
ppm
2400 2200 2000 1800 1600 1400 1200 1000 800 600 400 200 0

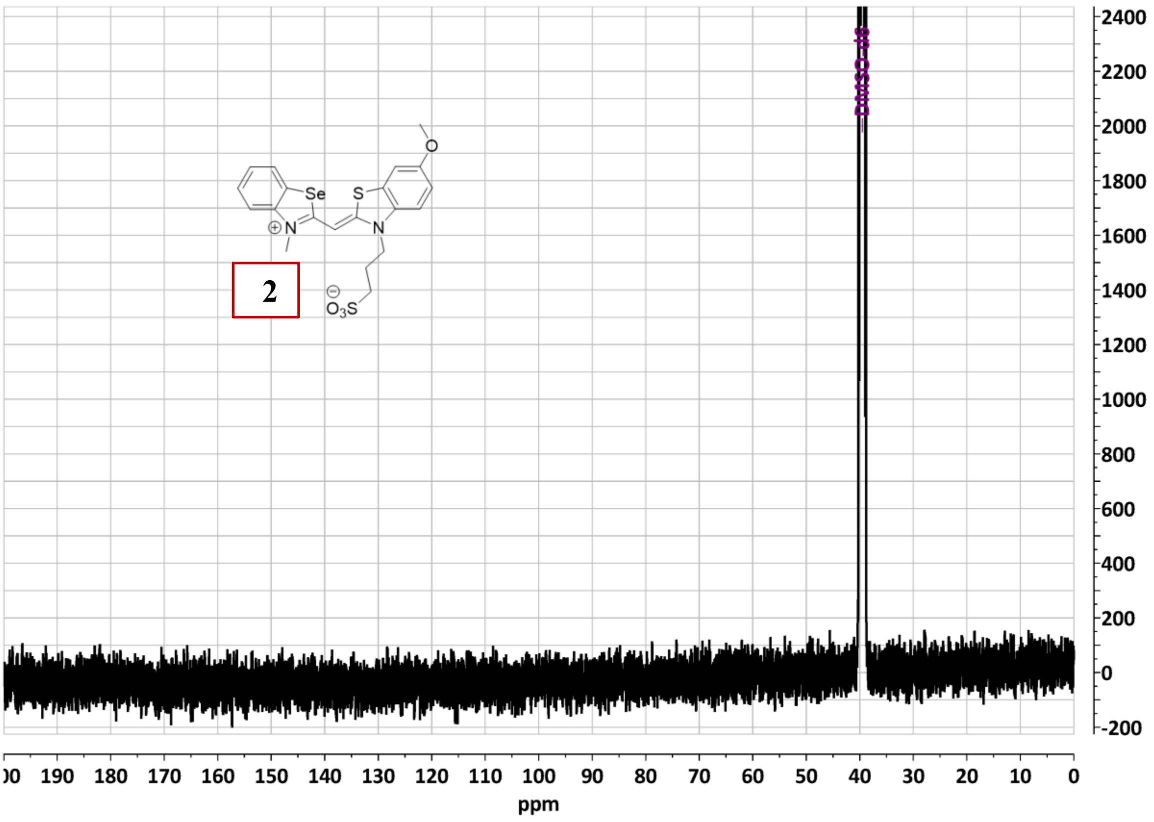
DMSO-d6
2
ppm

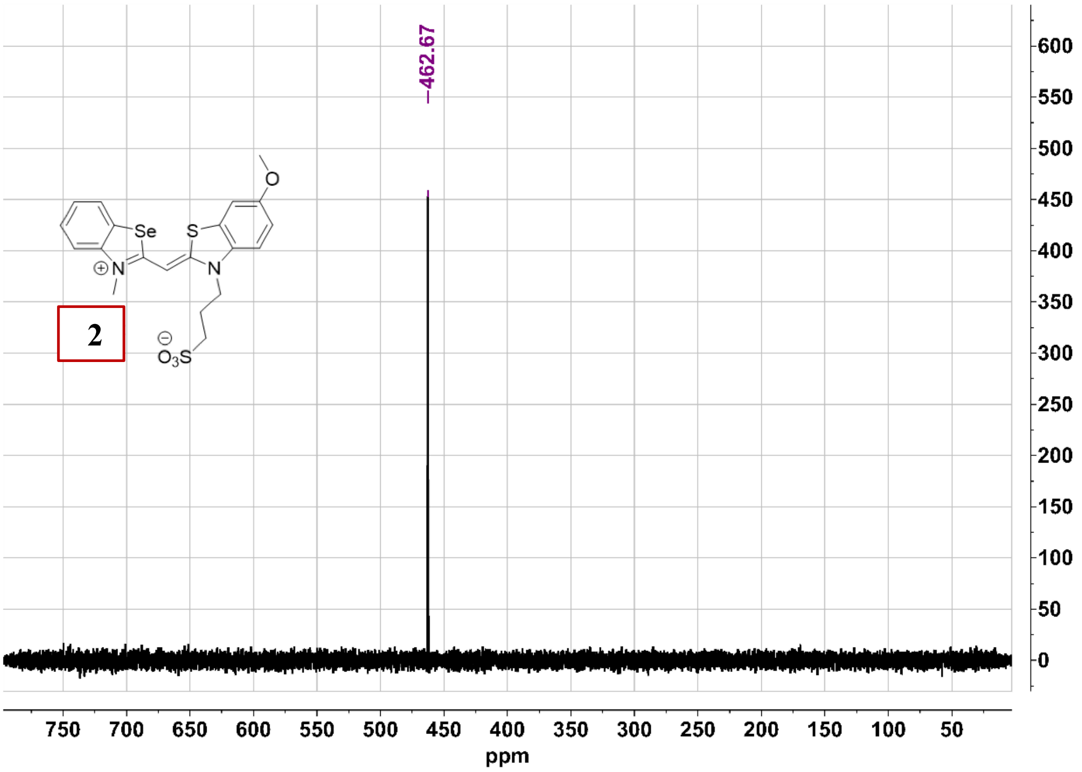
462.67
2
ppm

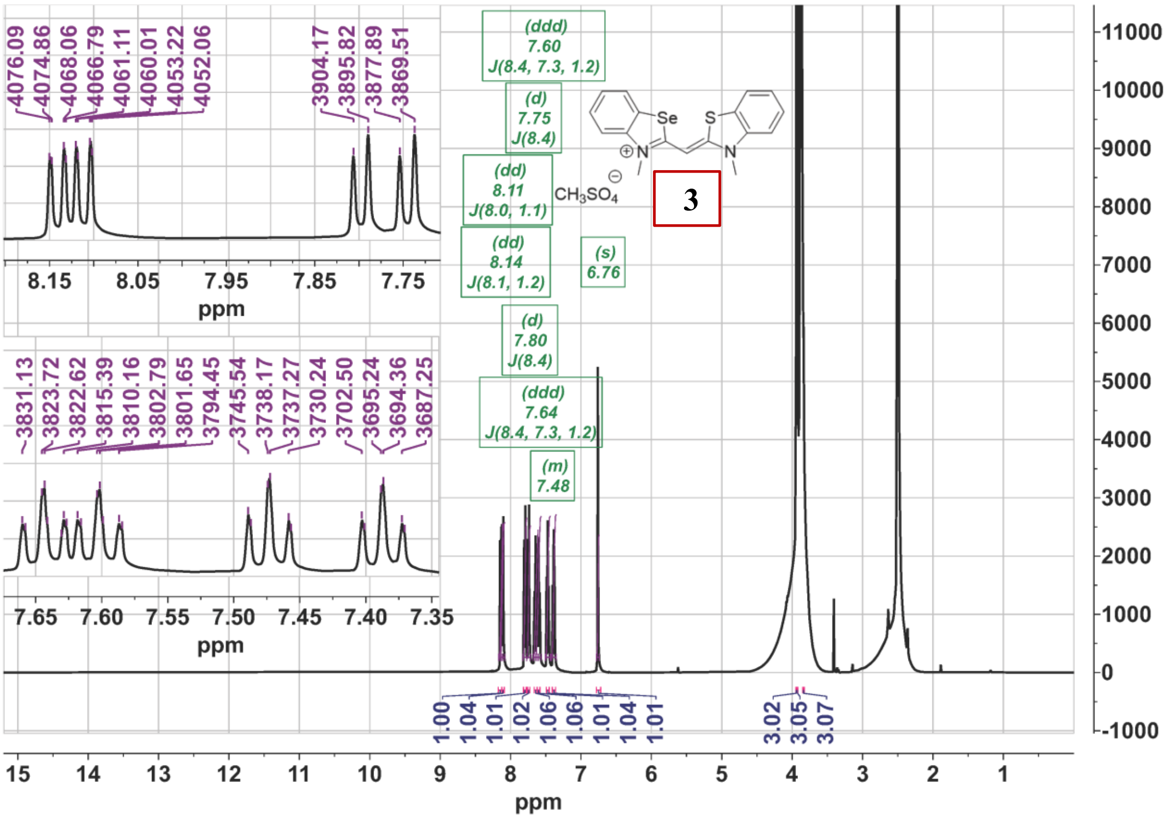
(ddd)
7.60
J(8.4, 7.3, 1.2)
(d)
7.75
J(8.4)
(dd)
8.11
J(8.0, 1.1)
(dd)
8.14
J(8.1, 1.2)
(s)
6.76
(d)
7.80
J(8.4)
(ddd)
7.64
J(8.4, 7.3, 1.2)
(m)
7.48
Se
S
N
N
CH3SO4
3
4076.09
4074.86
4068.06
4066.79
4061.11
4060.01
4053.22
4052.06
3904.17
3895.82
3877.89
3869.51
3831.13
3823.72
3822.62
3815.39
3810.16
3802.79
3801.65
3794.45
3745.54
3738.17
3737.27
3730.24
3702.50
3695.24
3694.36
3687.25
1.00
1.04
1.01
1.02
1.06
1.06
1.01
1.04
1.01
3.02
3.05
3.07
ppm

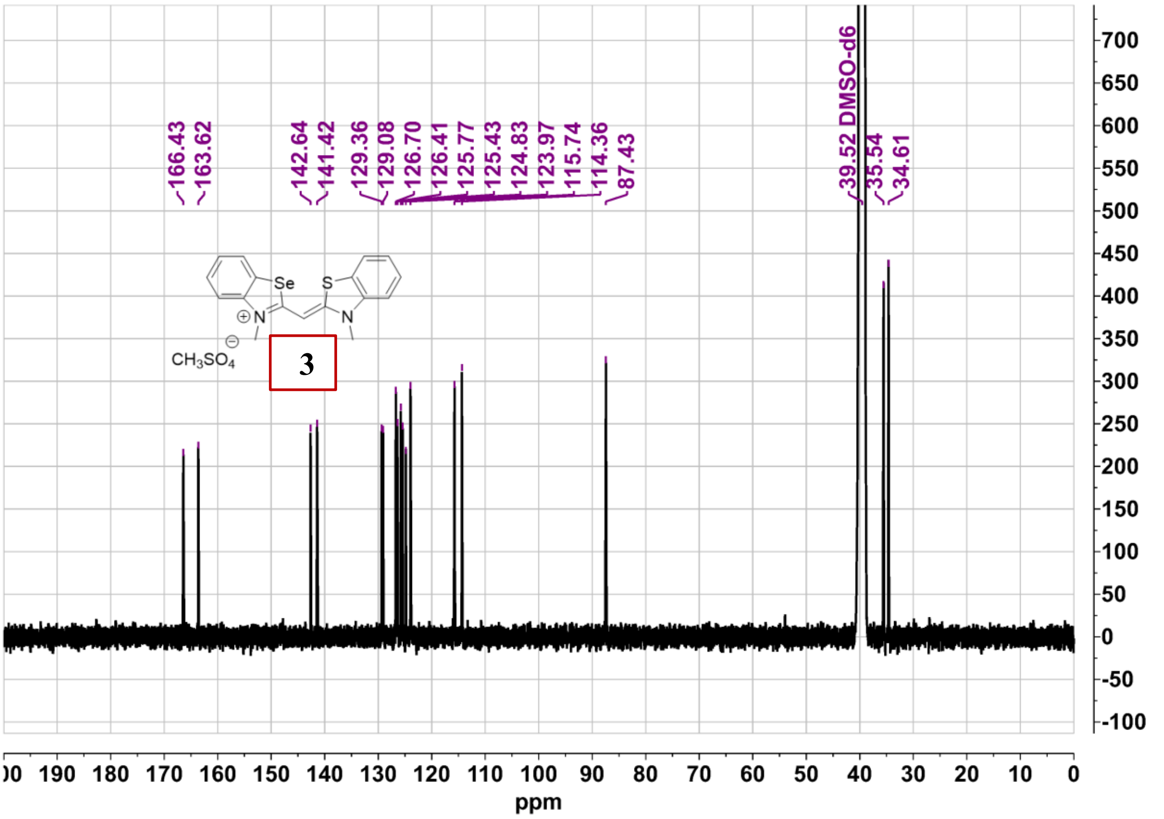
166.43
163.62
142.64
141.42
129.36
129.08
126.70
126.41
125.77
125.43
124.83
123.97
115.74
114.36
87.43
39.52 DMSO-d6
35.54
34.61
Se
S
N
N
CH3SO4
3
ppm

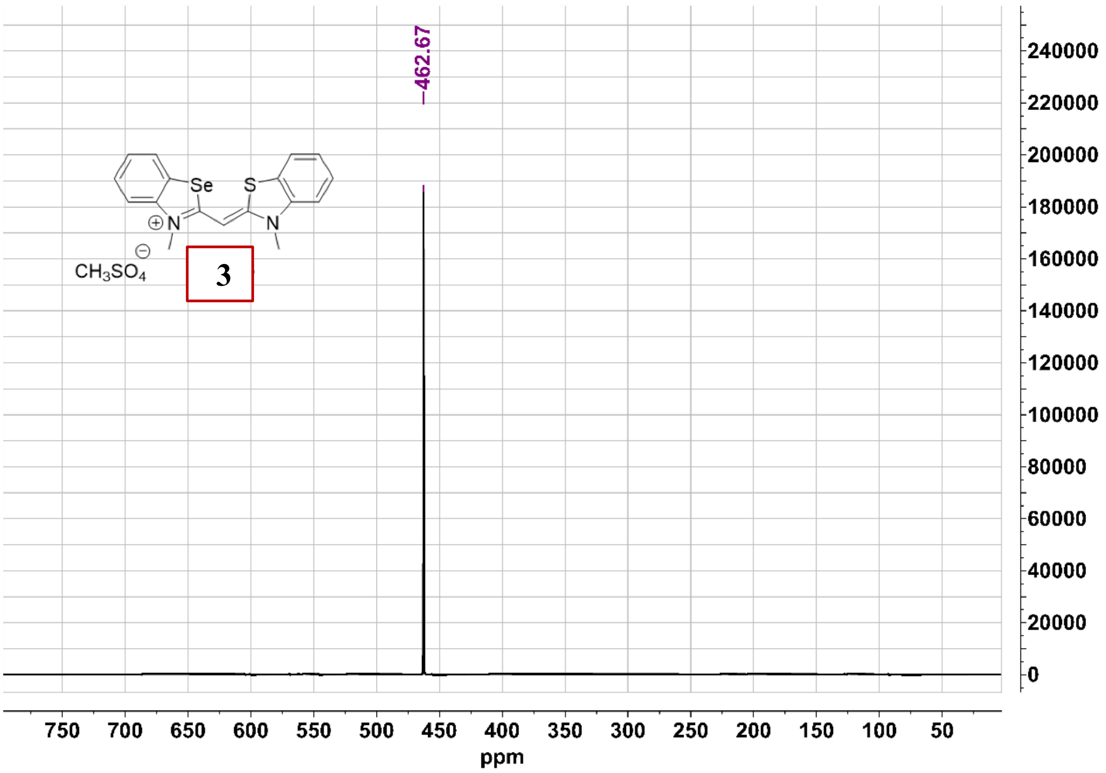

3
CH3SO4
Se
S
N
N
462.67
ppm

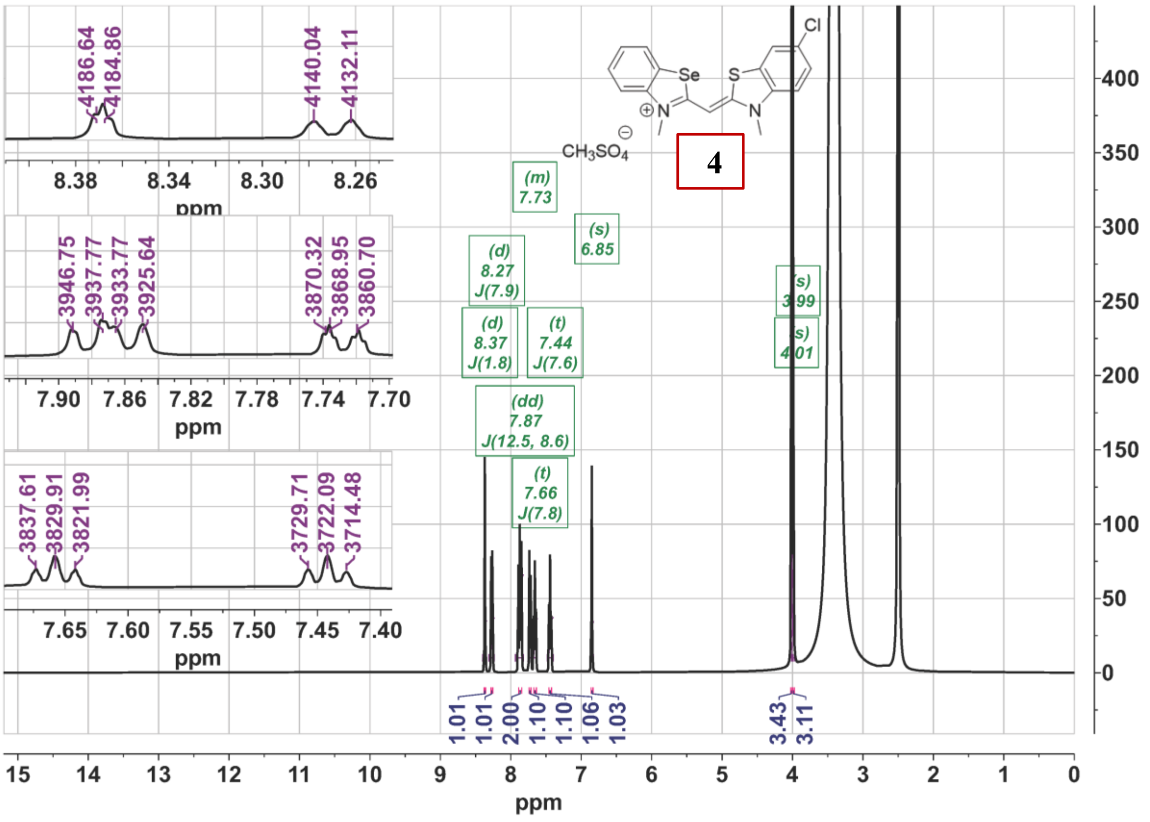

4
CH3SO4
Se
S
N
N
Cl
(d) 8.37 J(1.8)
(d) 8.27 J(7.9)
(dd) 7.87 J(12.5, 8.6)
(m) 7.73
(t) 7.66 J(7.8)
(t) 7.44 J(7.6)
(s) 6.85
(s) 4.01
(s) 3.99
ppm

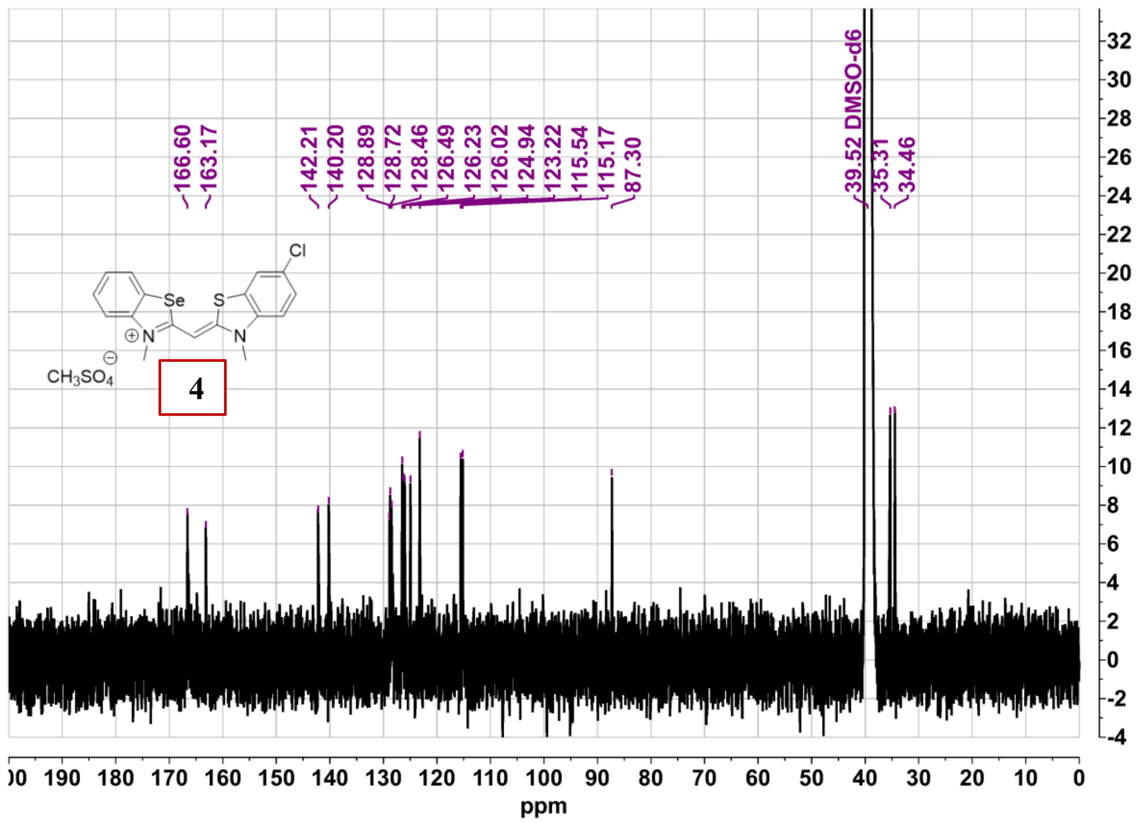
166.60
163.17
142.21
140.20
128.89
128.72
128.46
126.49
126.23
126.02
124.94
123.22
115.54
115.17
87.30
39.52 DMSO-d6
35.31
34.46
Cl
Se
S
N
N
CH_3SO_4
4
ppm

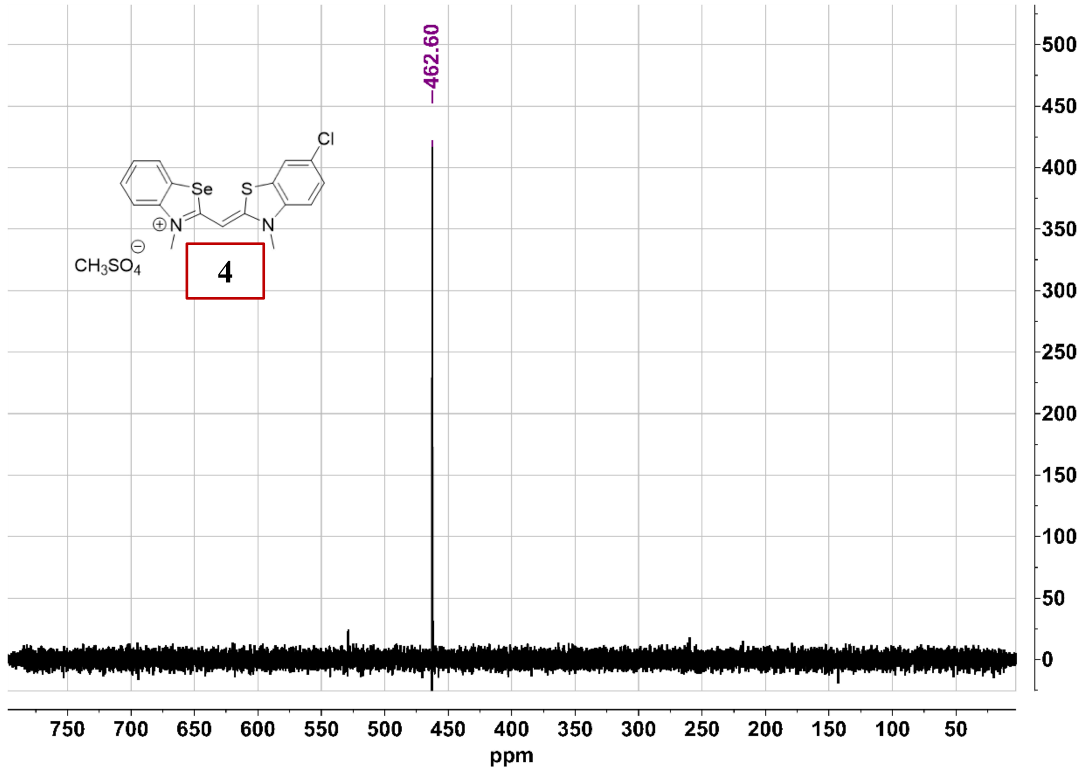
462.60
Cl
Se
S
N
N
CH_3SO_4
4
ppm

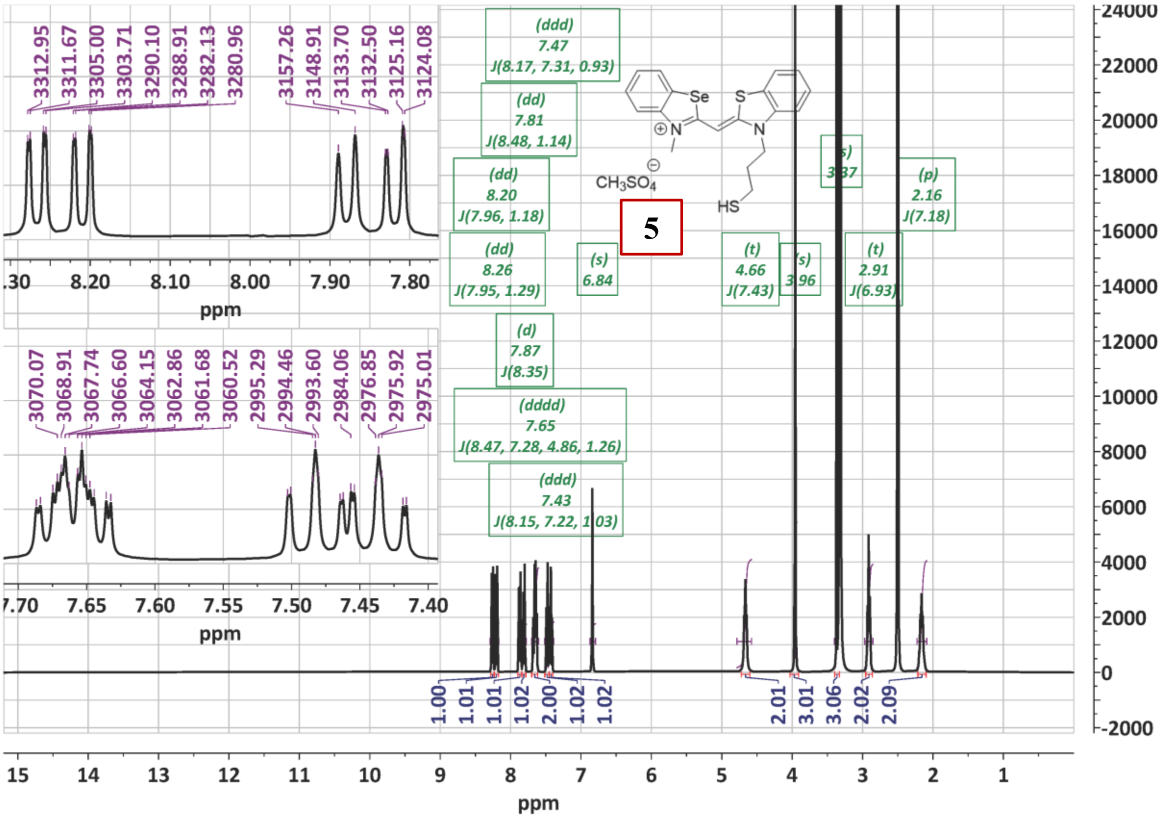

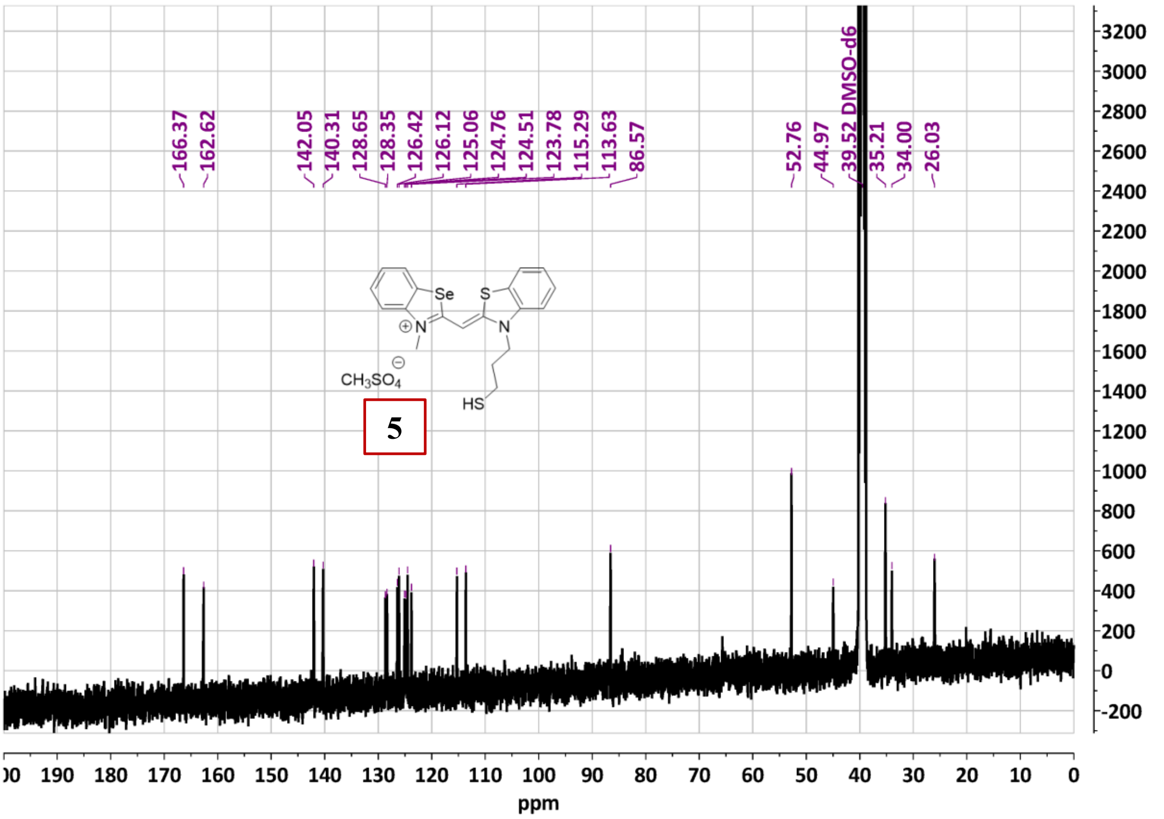

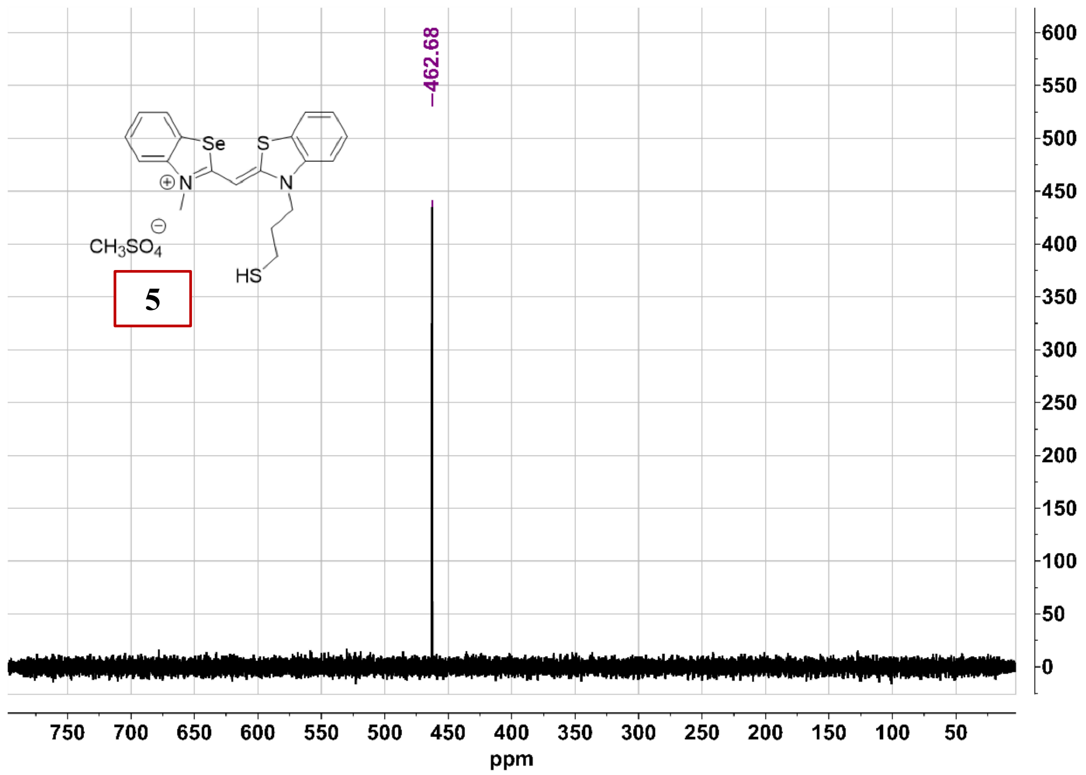

462.68
Se
S
N
N
CH3SO4
HS
5
ppm

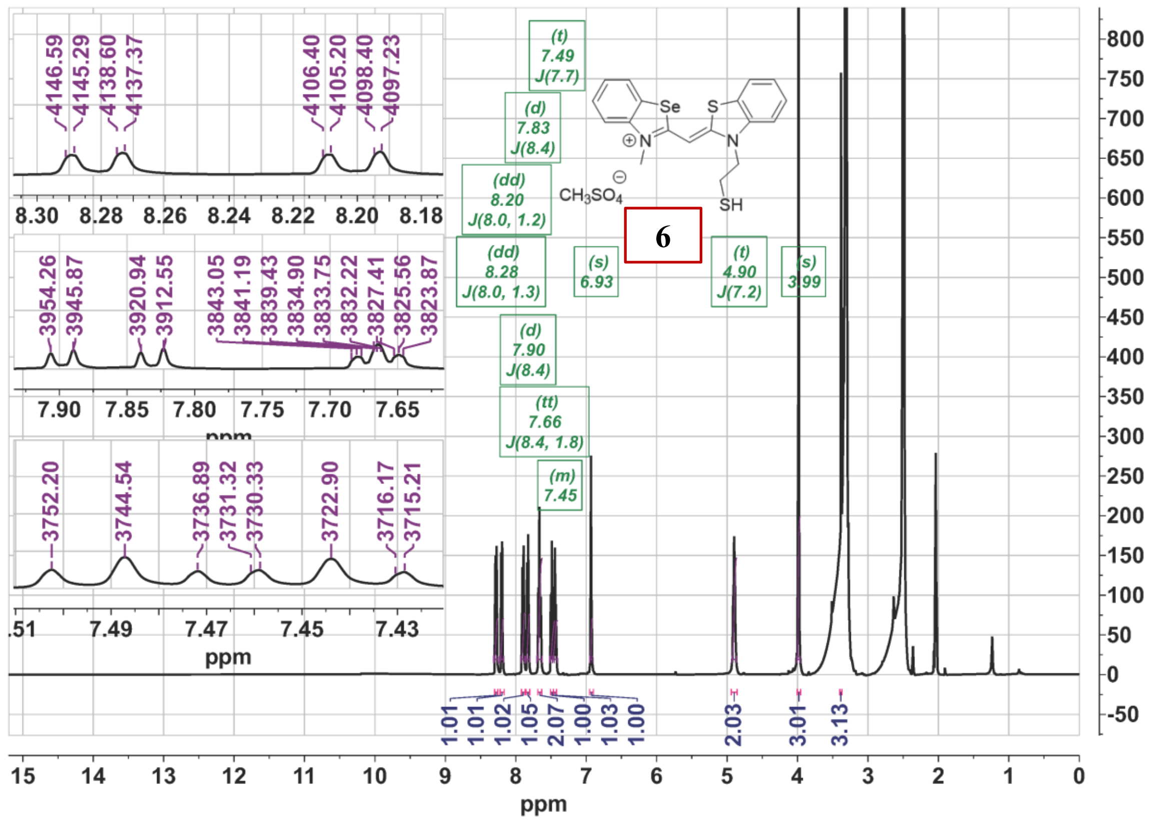

Se
S
N
N
CH3SO4
SH
6
(t) 7.49 J(7.7)
(d) 7.83 J(8.4)
(dd) 8.20 J(8.0, 1.2)
(dd) 8.28 J(8.0, 1.3)
(s) 6.93
(t) 4.90 J(7.2)
(s) 3.99
(d) 7.90 J(8.4)
(tt) 7.66 J(8.4, 1.8)
(m) 7.45
4146.59 4145.29 4138.60 4137.37 4106.40 4105.20 4098.40 4097.23
3954.26 3945.87 3920.94 3912.55 3843.05 3841.19 3839.43 3834.90 3833.75 3832.22 3827.41 3825.56 3823.87
3752.20 3744.54 3736.89 3731.32 3730.33 3722.90 3716.17 3715.21
1.01 1.01 1.02 1.05 2.07 1.00 1.03 1.00 2.03 3.01 3.13
ppm

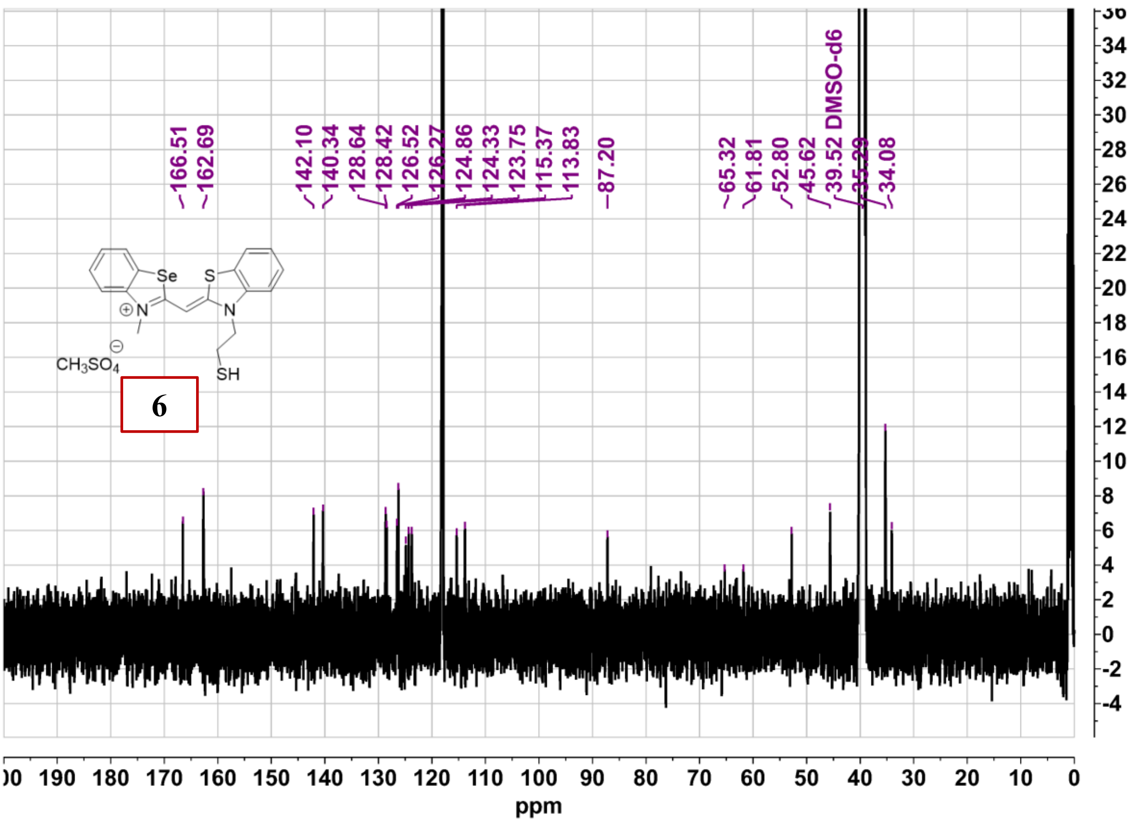
166.51
162.69
142.10
140.34
128.64
128.42
126.52
126.27
124.86
124.33
123.75
115.37
113.83
87.20
65.32
61.81
52.80
45.62
39.52 DMSO-d6
35.29
34.08
Se
S
N
N
CH3SO4
SH
6
ppm

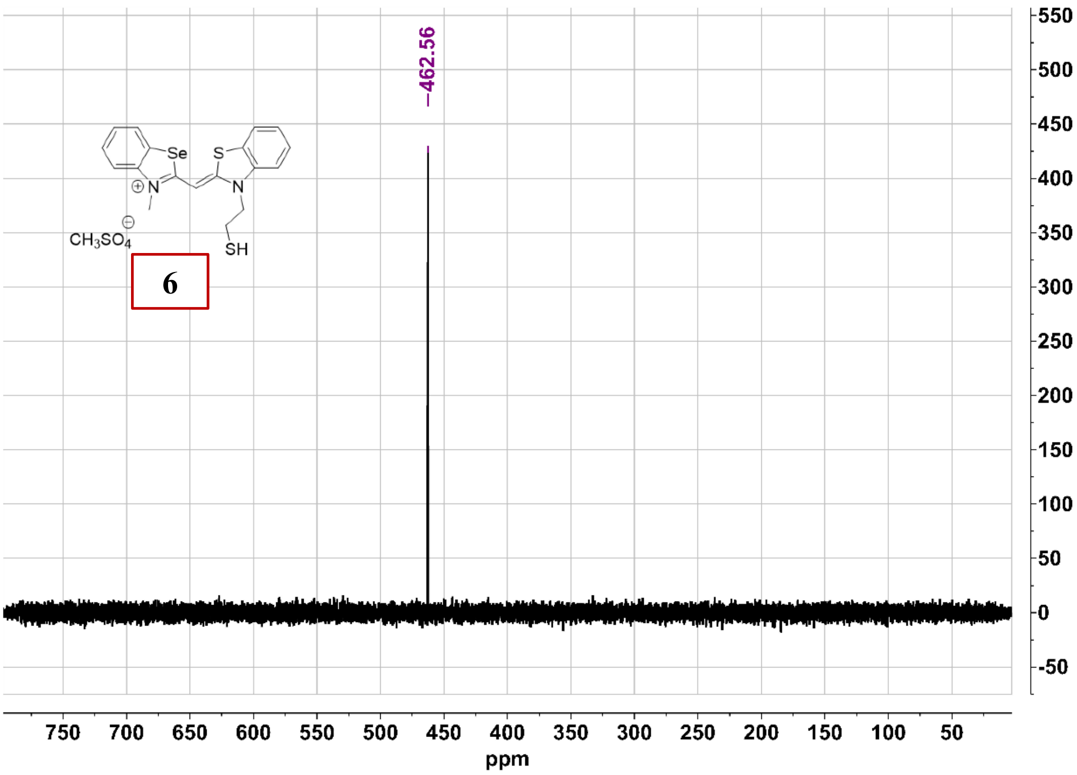
462.56
Se
S
N
N
CH3SO4
SH
6
ppm

Window Display Report

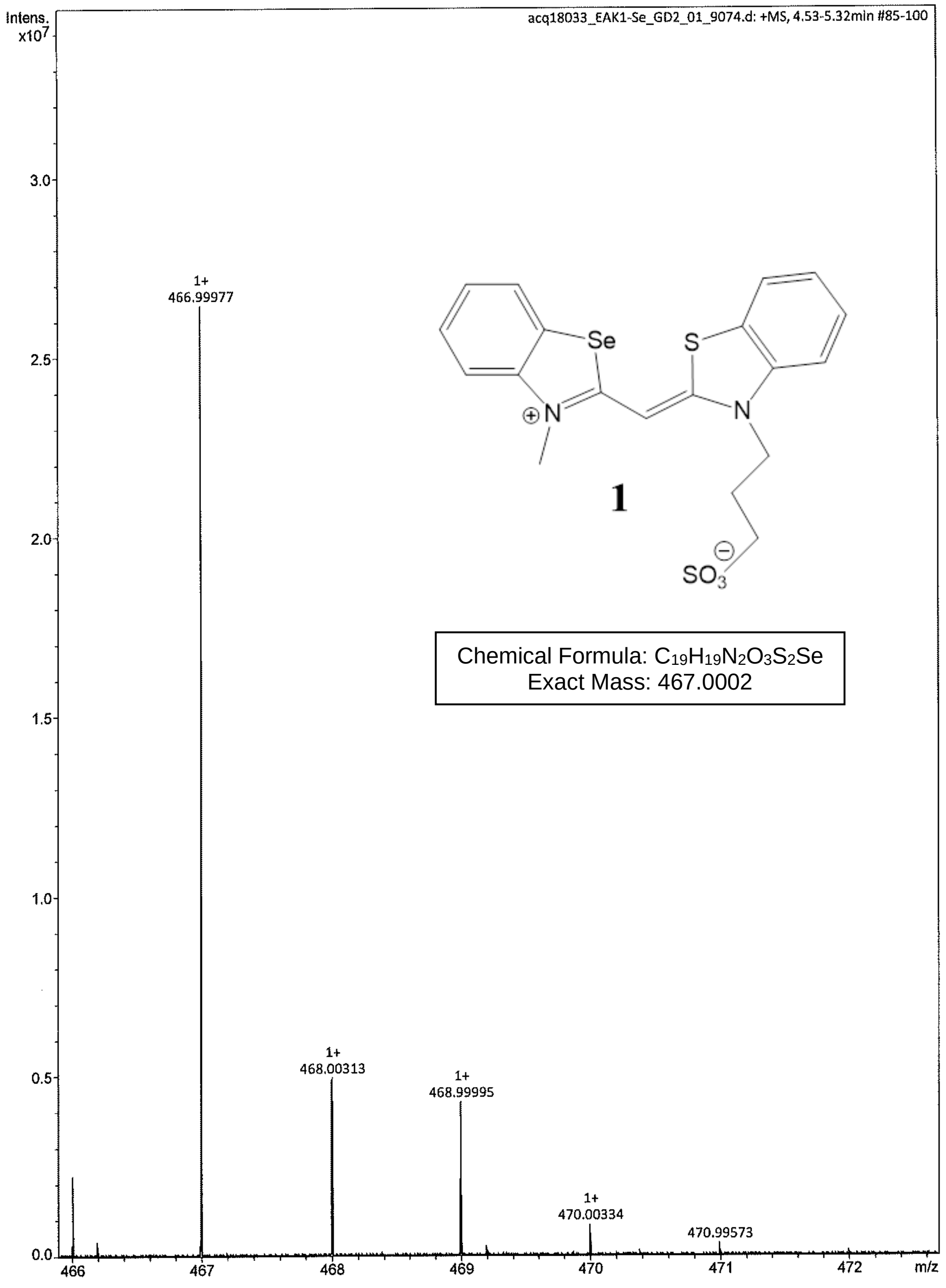

Window Display Report

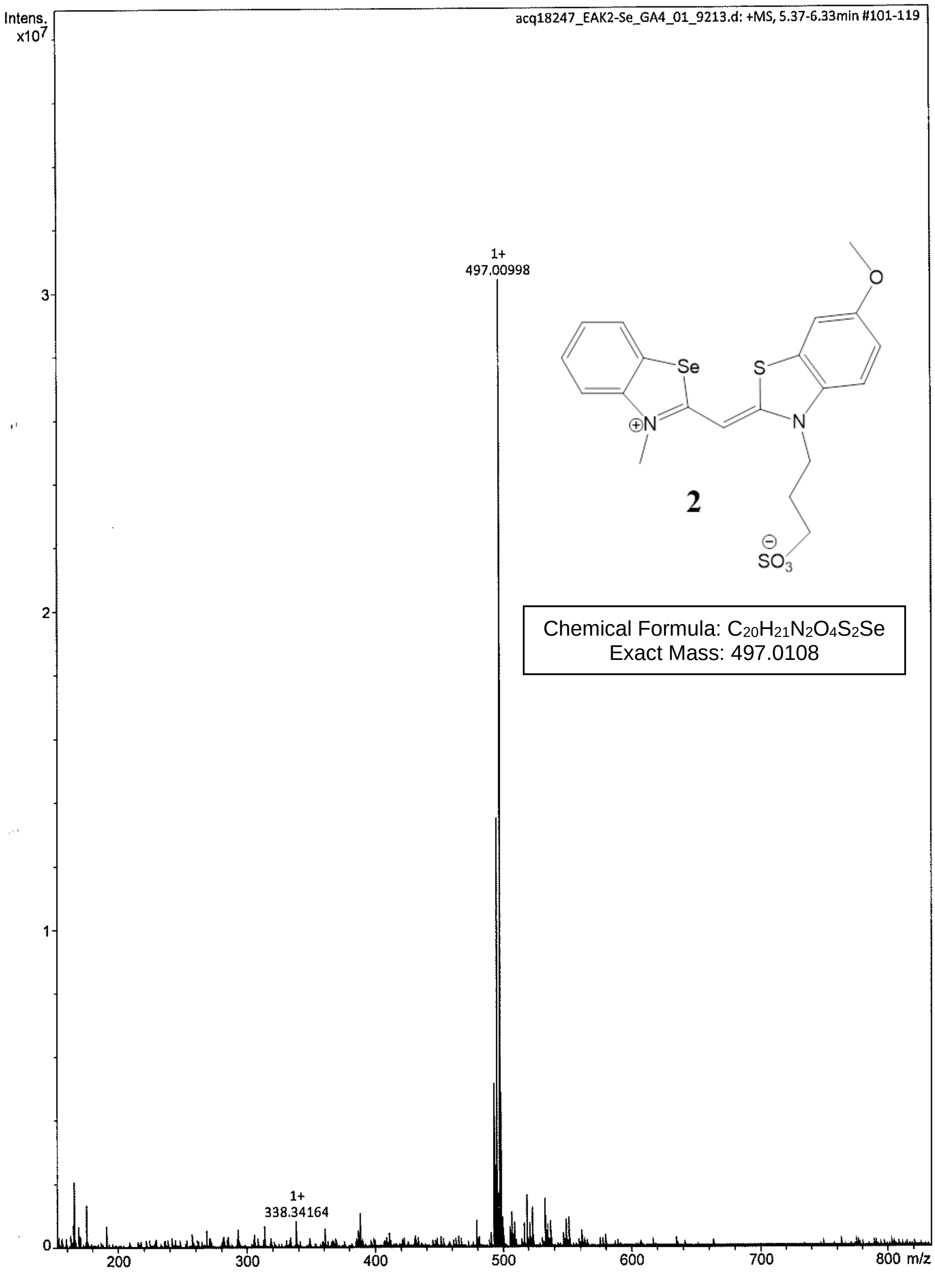

2

Chemical Formula: $C_{20}H_{21}N_2O_4S_2Se$
Exact Mass: 497.0108

Window Display Report

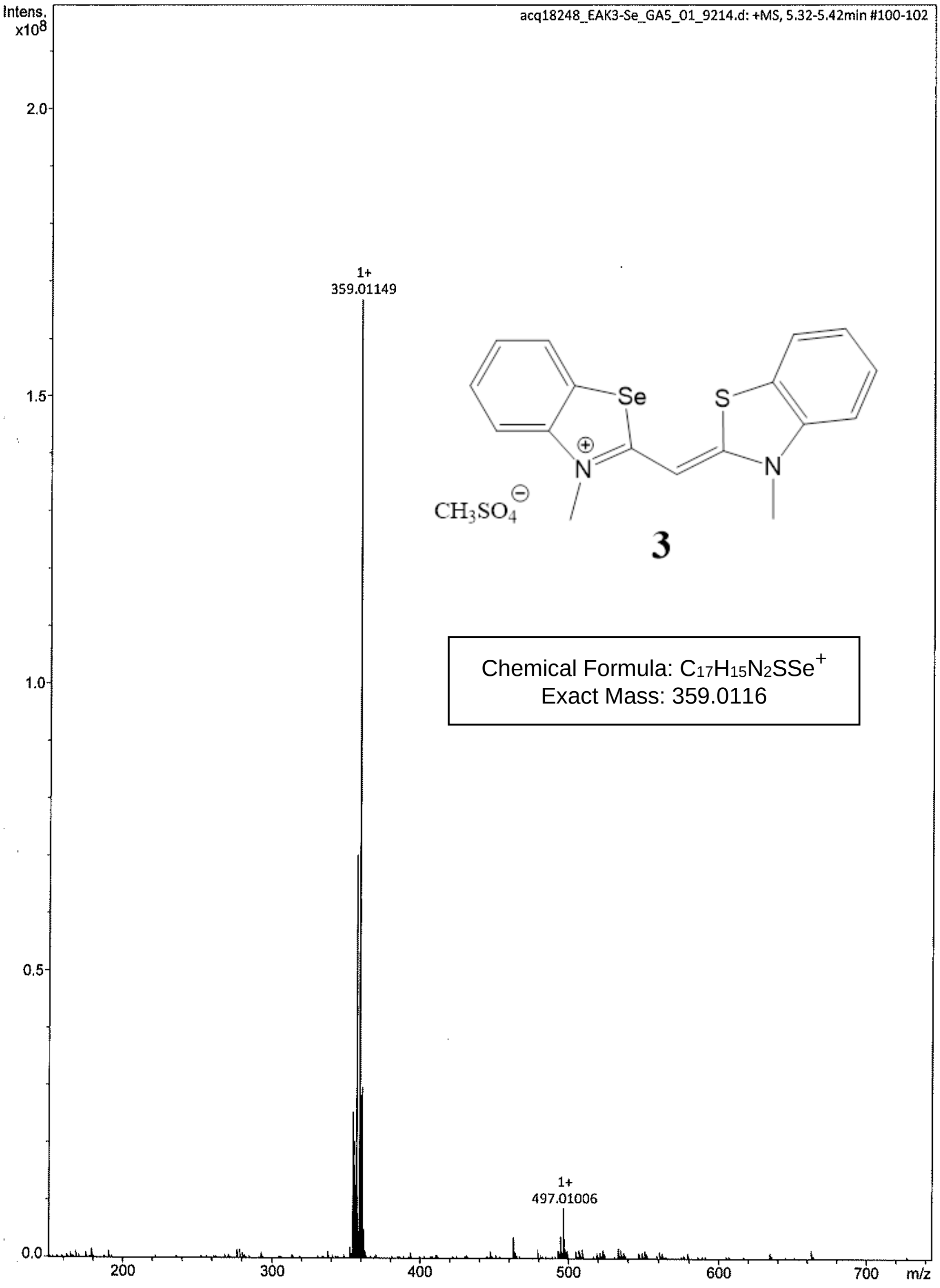

Window Display Report

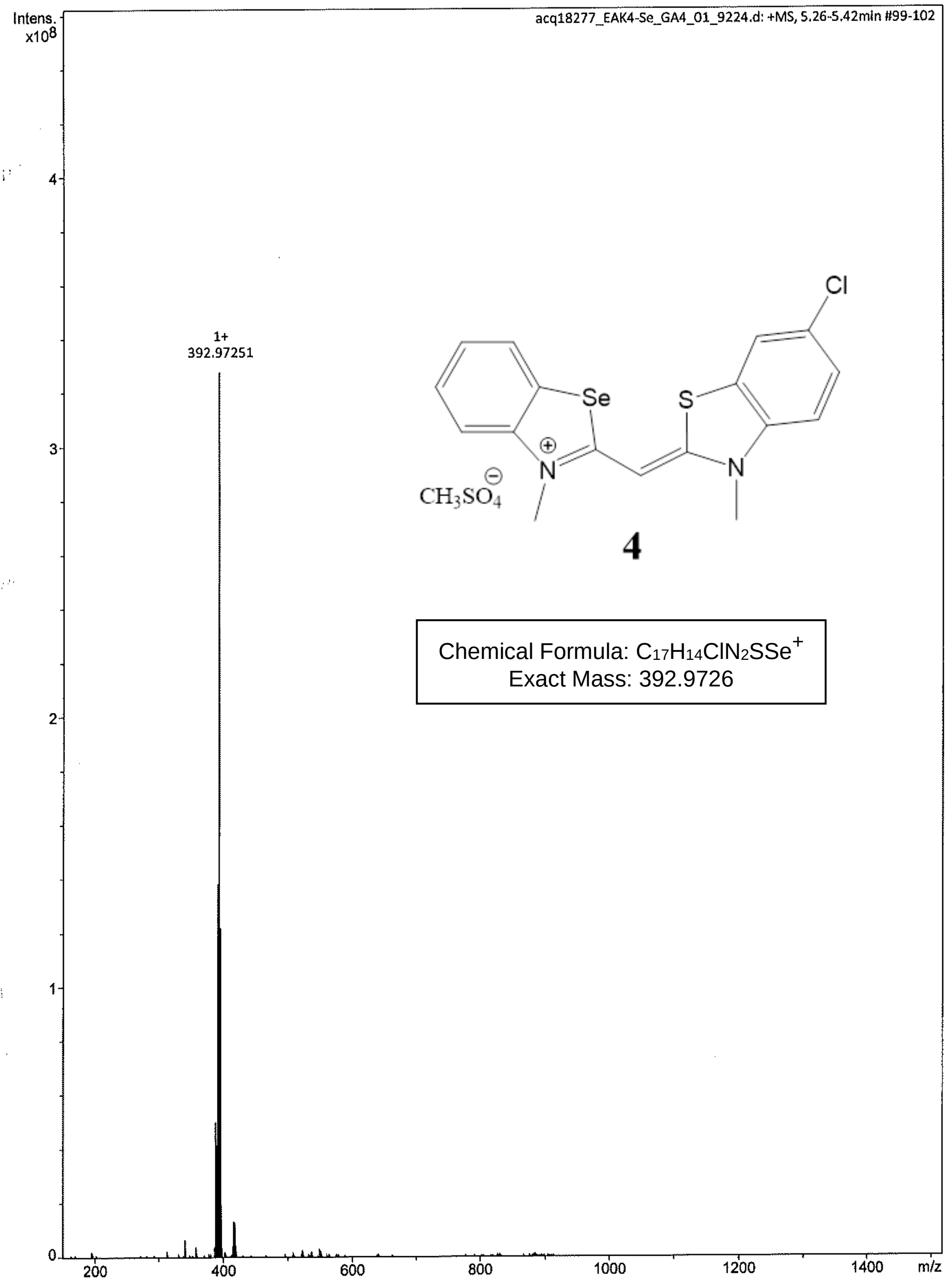

Window Display Report

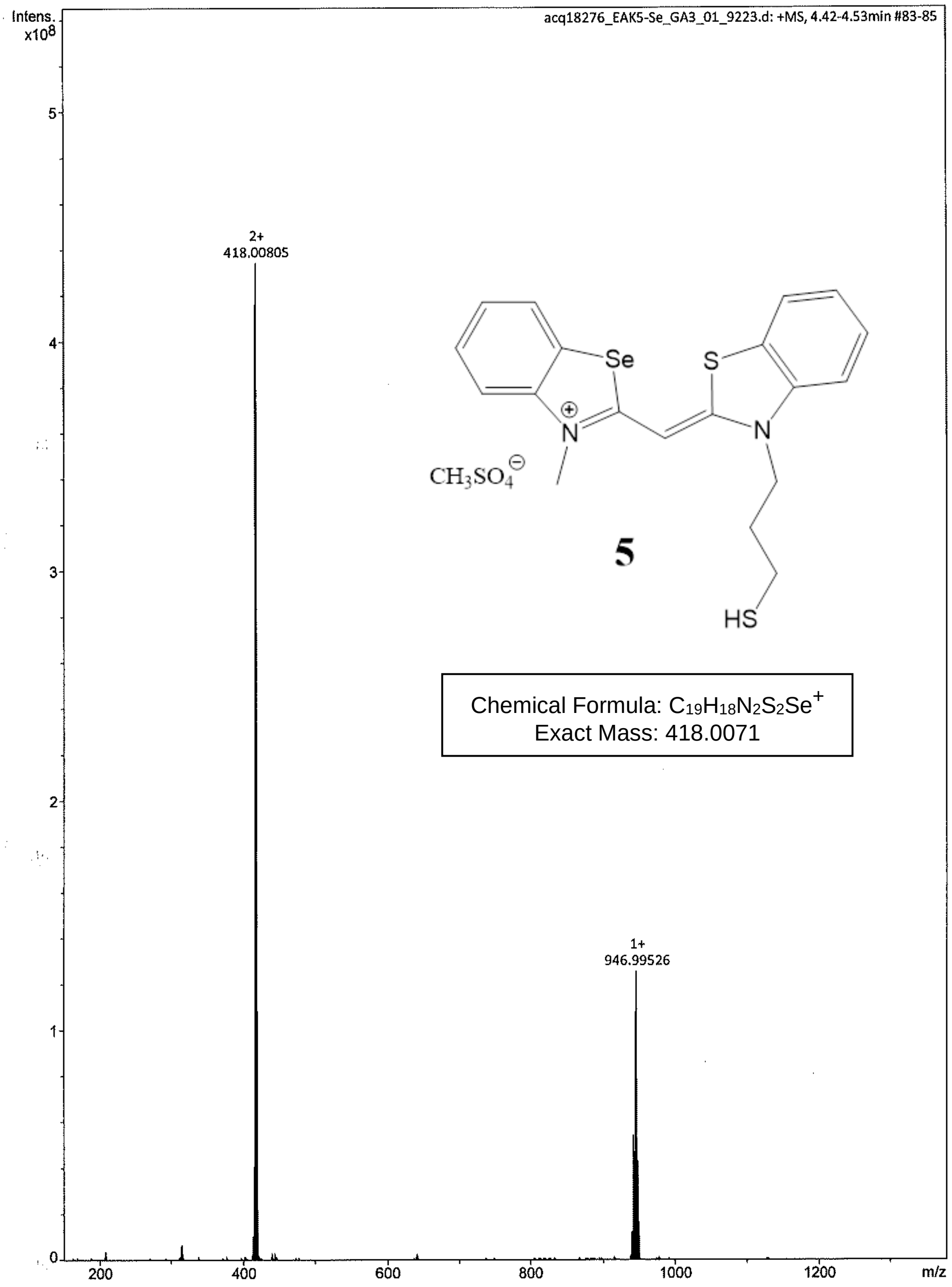

Window Display Report

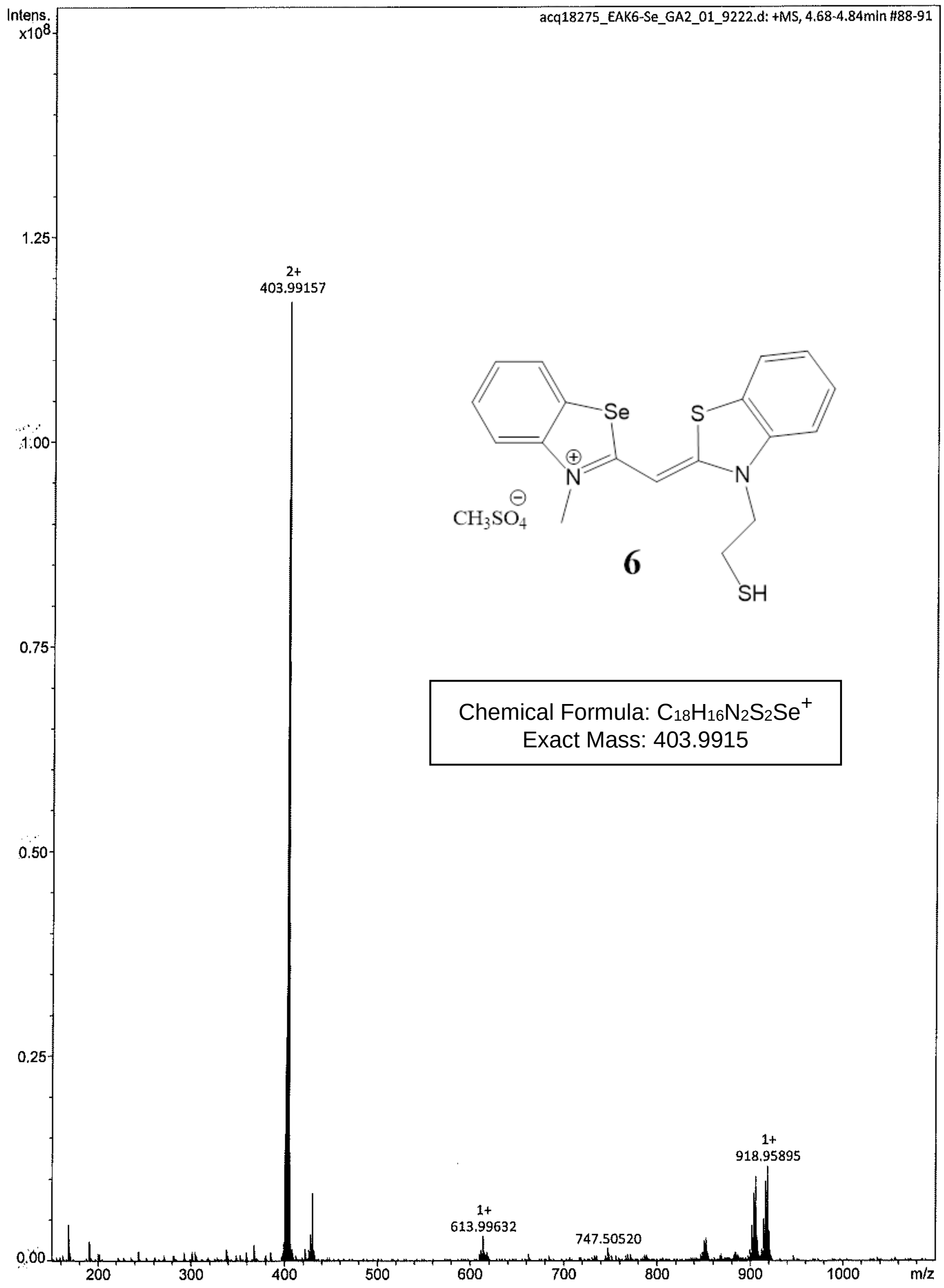

The obtained results form the 1H-NMR, 77Se-NMR and HRMS for dye 2, were found to be in absolute agreement to the expected chemical structure. Due to the relatively poor solubility leading to low concentration and rather diminished sensitivity for attempting to record 13C-NMR and DEPT (even at 600 MHz), the 20 carbon signals belonging to the dye 2 were acquired from 2D-NMR techniques. Namely, the cross peaks were taken from the HSQC and HMBC NMR techniques also recorded in DMSO-d6. All signals were compared to dye 1, which resembles the same chemical structure without a methoxy group on the benzoselenazolium core; δ 23.34, 35.82, 45.41, 47.63, 56.35, 87.66, 99.12, 113.83, 115.55, 116.24, 124.62, 124.96, 126.50, 128.55, 128.67, 142.28, 142.75, 160.82, 161.06, 166.22.

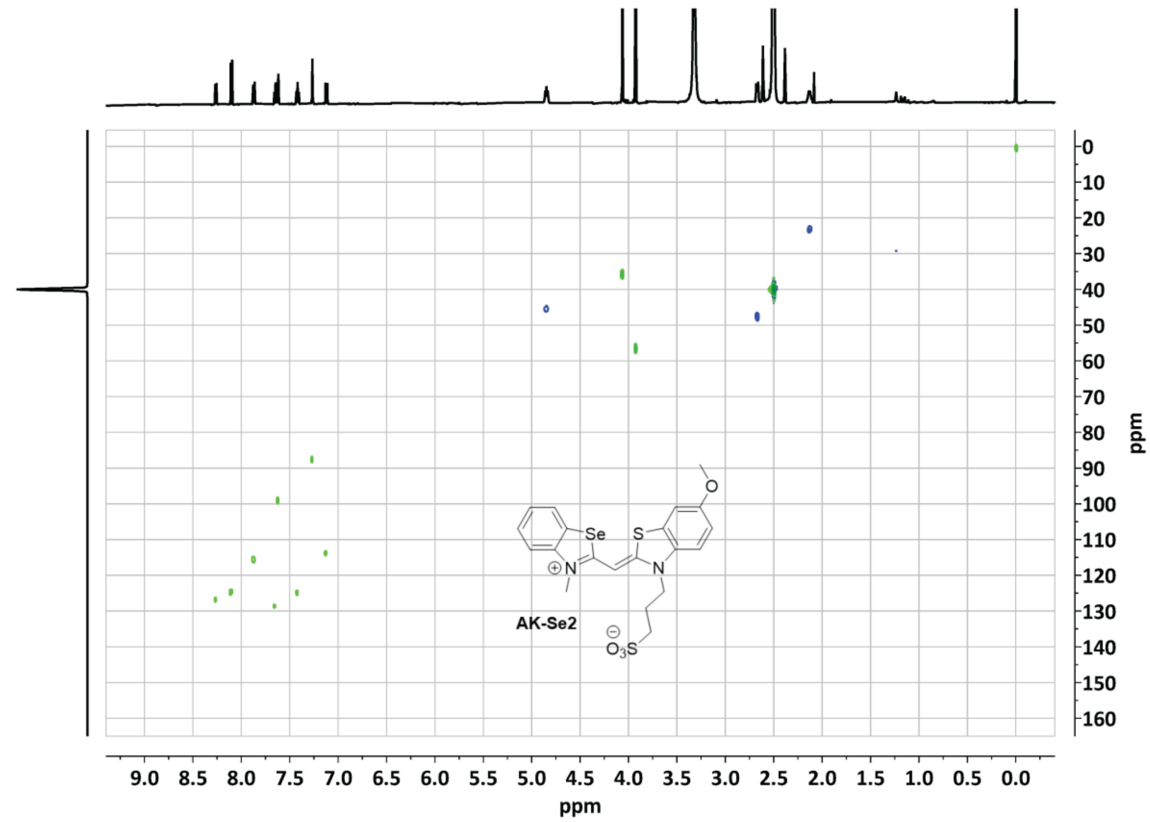

AK-Se2
ppm
ppm

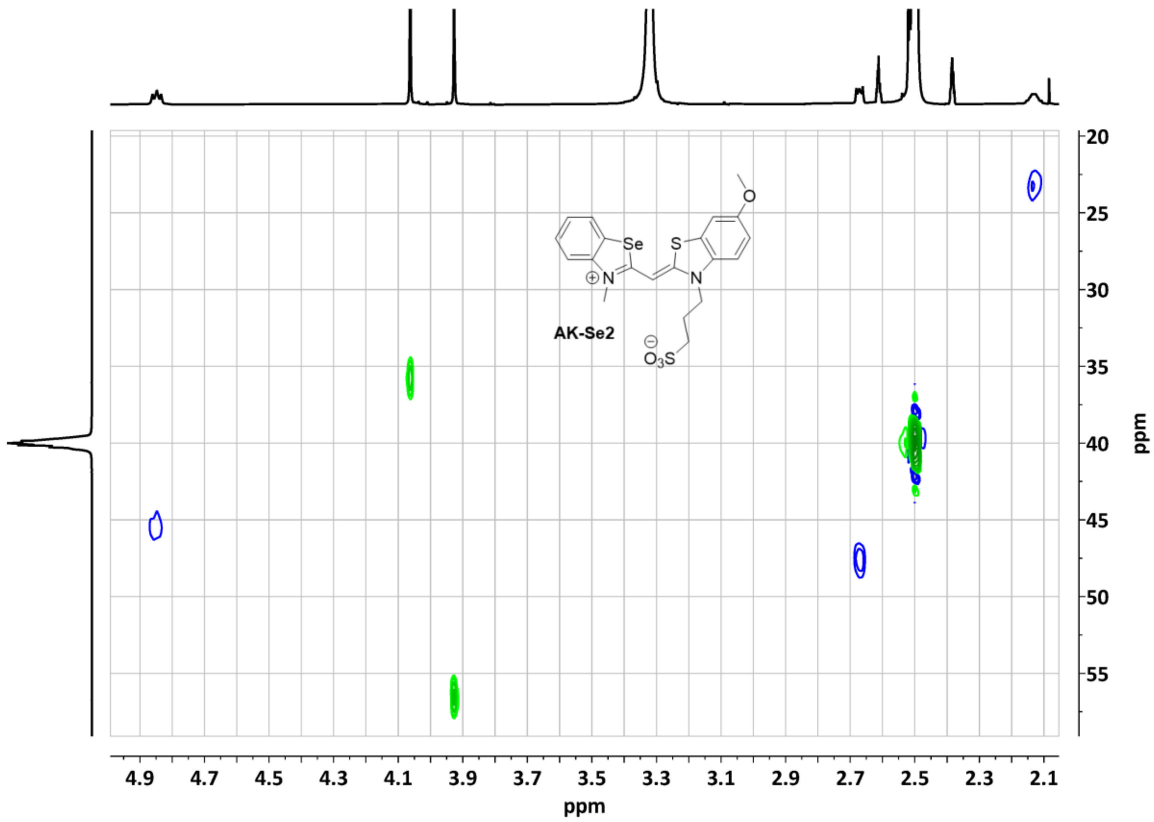

AK-Se2
ppm
ppm

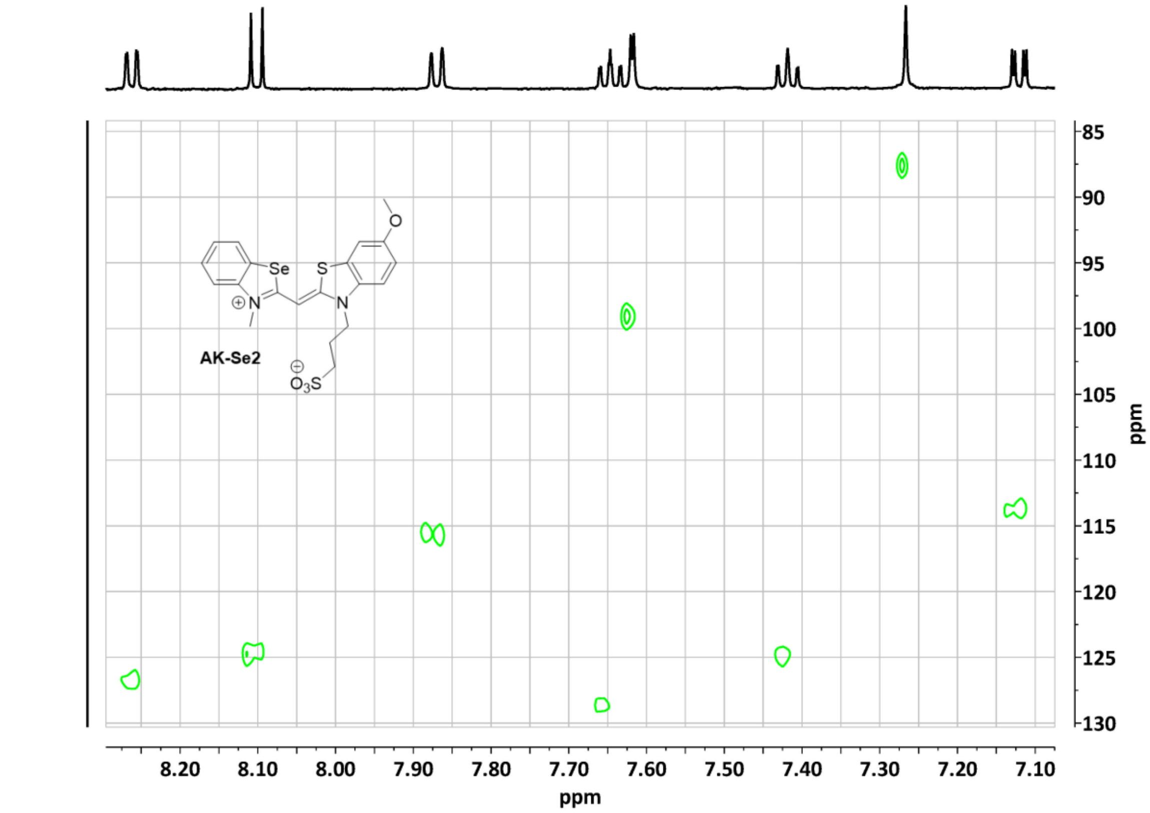
Se
S
N
N
O
O_3S
AK-Se2
ppm
ppm
8.20
8.10
8.00
7.90
7.80
7.70
7.60
7.50
7.40
7.30
7.20
7.10
85
90
95
100
105
110
115
120
125
130

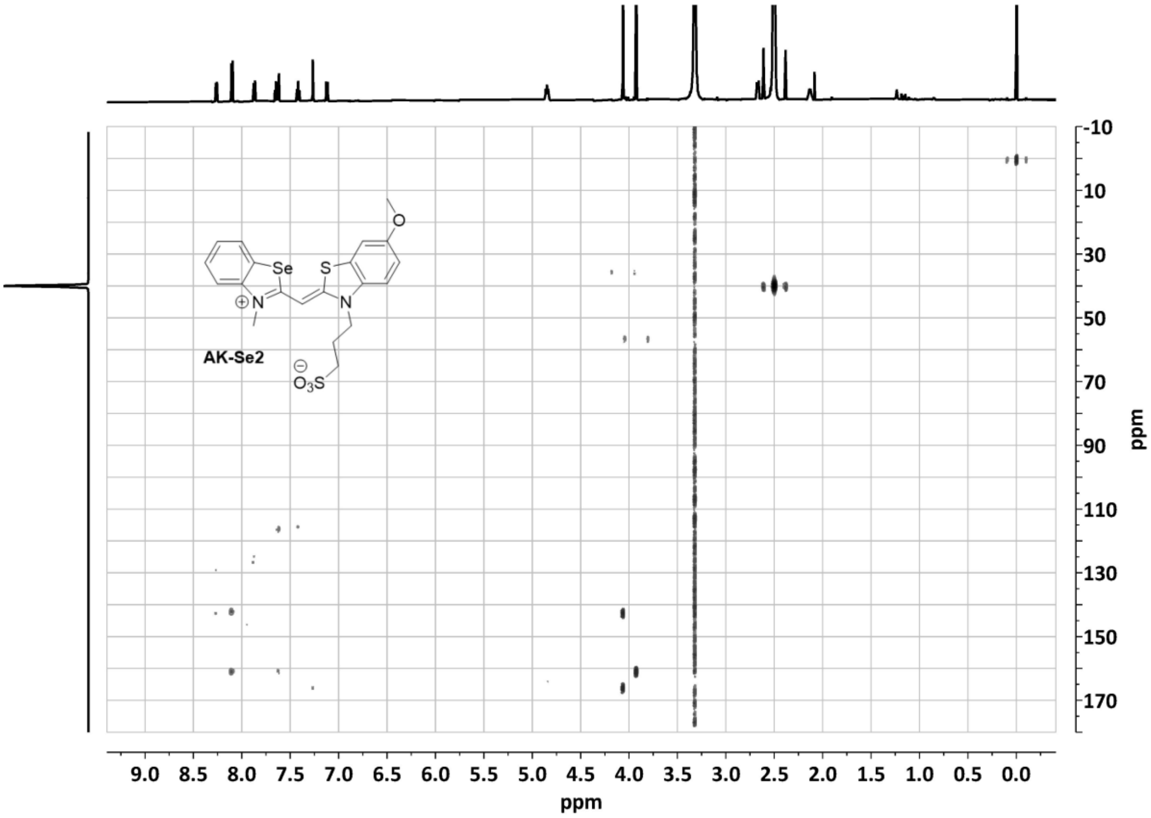
Se
S
N
N
O
O_3S
AK-Se2
ppm
ppm
9.0
8.5
8.0
7.5
7.0
6.5
6.0
5.5
5.0
4.5
4.0
3.5
3.0
2.5
2.0
1.5
1.0
0.5
0.0
-10
10
30
50
70
90
110
130
150
170

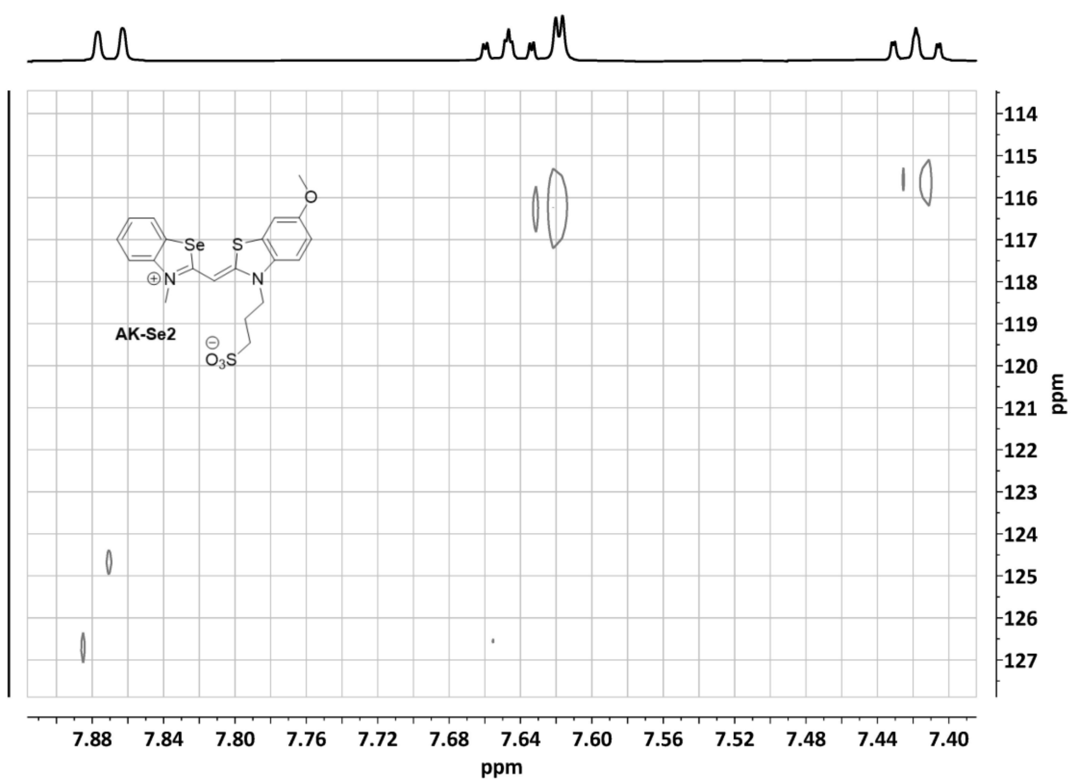
AK-Se2
ppm
ppm

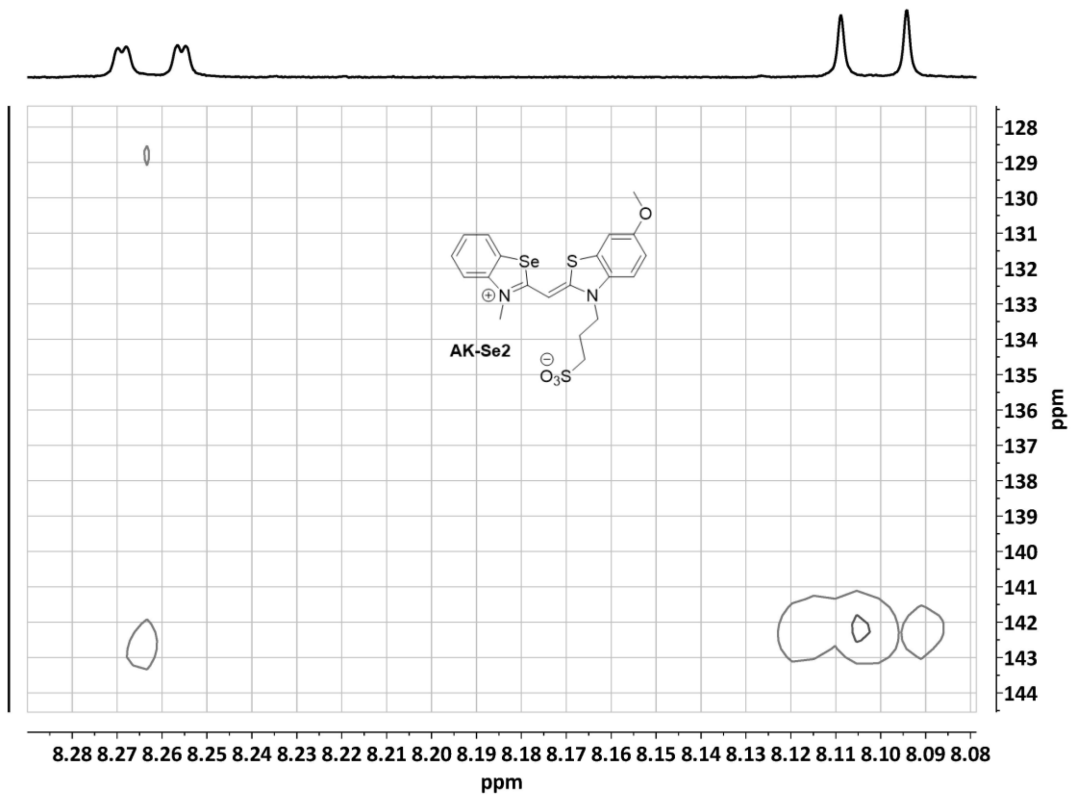
AK-Se2
ppm
ppm

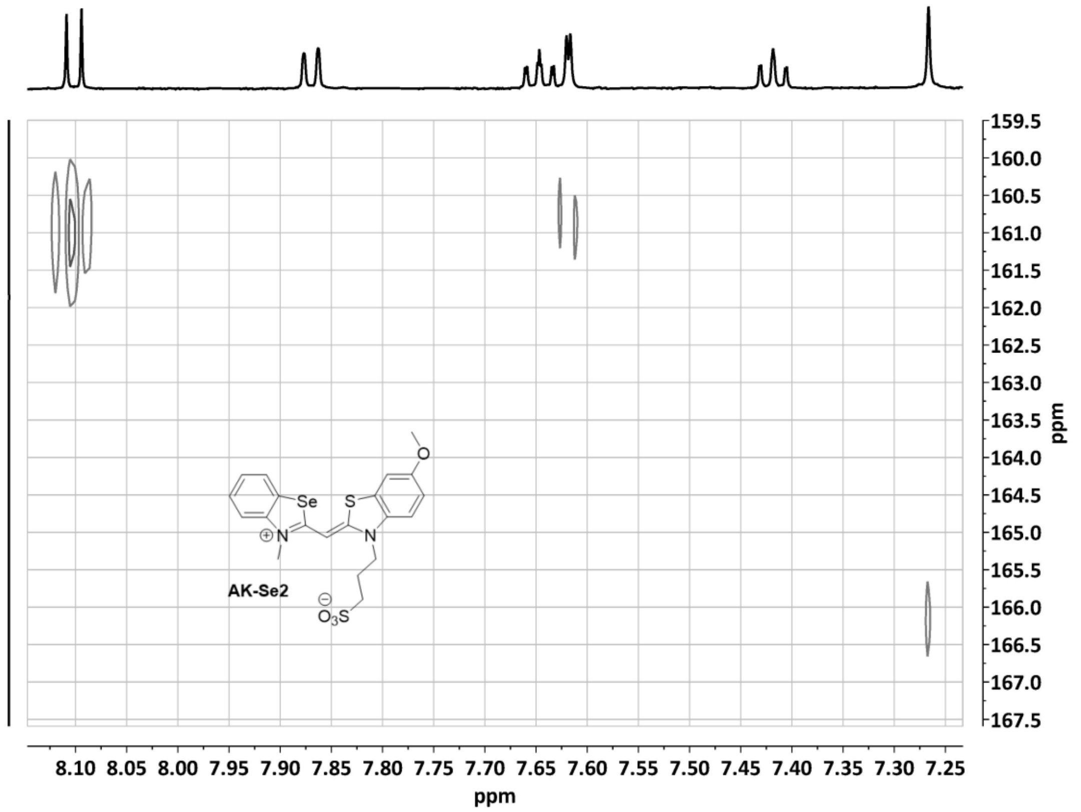
AK-Se2
ppm
ppm

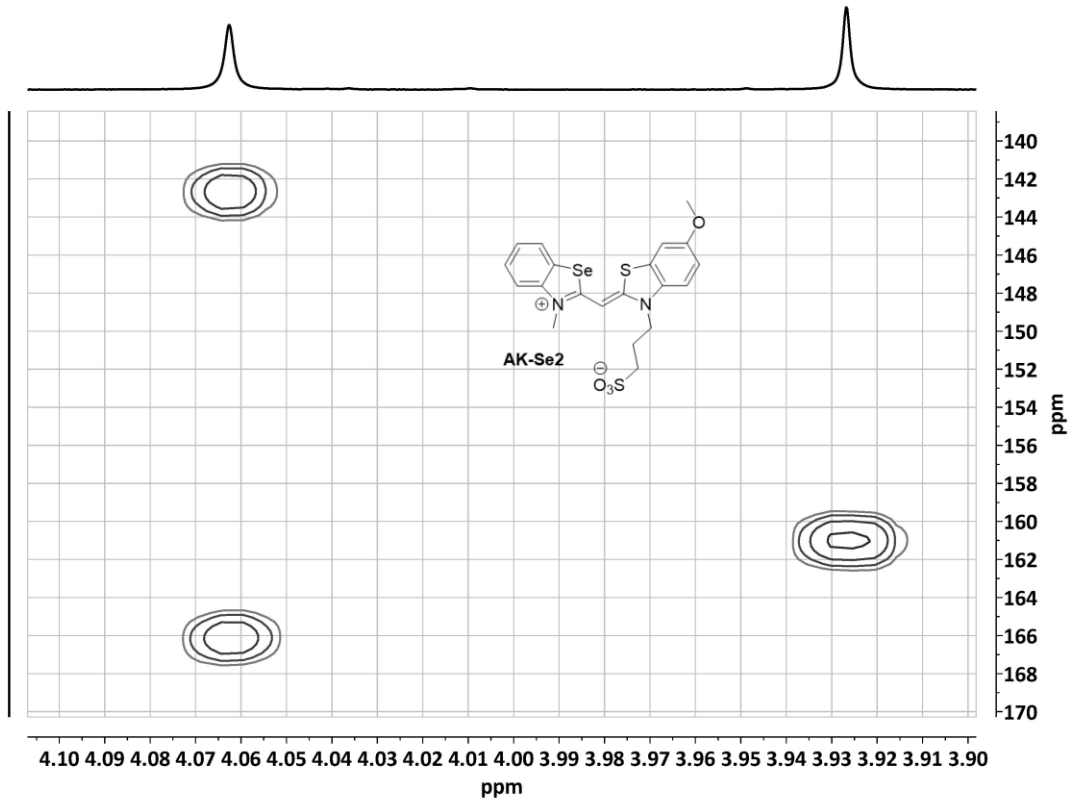
AK-Se2
ppm
ppm
